# Structure of two purple pigments, catechinopyranocyanidins A and B from the seed-coat of the small red bean, *Vigna angularis*

**DOI:** 10.1038/s41598-018-37641-0

**Published:** 2019-02-06

**Authors:** Kumi Yoshida, Nobukazu Nagai, Yoshiki Ichikawa, Miki Goto, Kohei Kazuma, Kin-ichi Oyama, Kazushi Koga, Masaru Hashimoto, Satoru Iuchi, Yoshiaki Takaya, Tadao Kondo

**Affiliations:** 1 Graduate School of Informatics, Nagoya University, Chikusa, Nagoya, 464-8601 Japan; 2 Graduate School of Information Sciences, Nagoya University, Chikusa, Nagoya, 464-8601 Japan; 3 Graduate School of Human Informatics, Nagoya University, Chikusa, Nagoya, 464-8601 Japan; 4 Research Center for Materials Science, Nagoya University, Chikusa, Nagoya, 464-8602 Japan; 5 Graduate School of Bioagricultural Sciences, Nagoya University, Chikusa, Nagoya, 464-8601 Japan; 6 Faculty of Agriculture and Life Science, Hirosaki University, 3 Bunkyo-cho, Hirosaki, 036-8561 Japan; 7 Faculty of Pharmacy, Meijo University, 150 Yagotoyama, Tenpaku, Nagoya, 468-8503 Japan

**Keywords:** Structure elucidation, Natural products

## Abstract

The small red bean, *Vigna angularis*, is primarily used to produce the “an-paste” component of Japanese sweets. Through the manufacturing process, the red seed-coat pigment is transferred to the colorless “an-particles”, imparting a purple color. However, the major pigment in the seed coat has not yet been identified, although it is historically presumed to be an anthocyanin. Here, we report the isolation and structural determination of two hydrophobic purple pigments in the seed coat via instrumental analysis and derivatization. The new pigments, catechinopyranocyanidins A and B, contain a novel pyranoanthocyanidin skeleton condensed with a catechin and cyanidin ring system, and no sugar moieties. Catechinopyranocyanidins A and B are diastereomers with a different configuration at the catechin moiety, and both are purple in color in strongly acidic-to-neutral media. Catechinopyranocyanidins A and B are very stable under dark conditions, but, labile to light and decompose to colorless compounds. Thus, these pigments exhibit quite different chemical properties compared to simple anthocyanidins.

## Introduction

**Figure 1:**
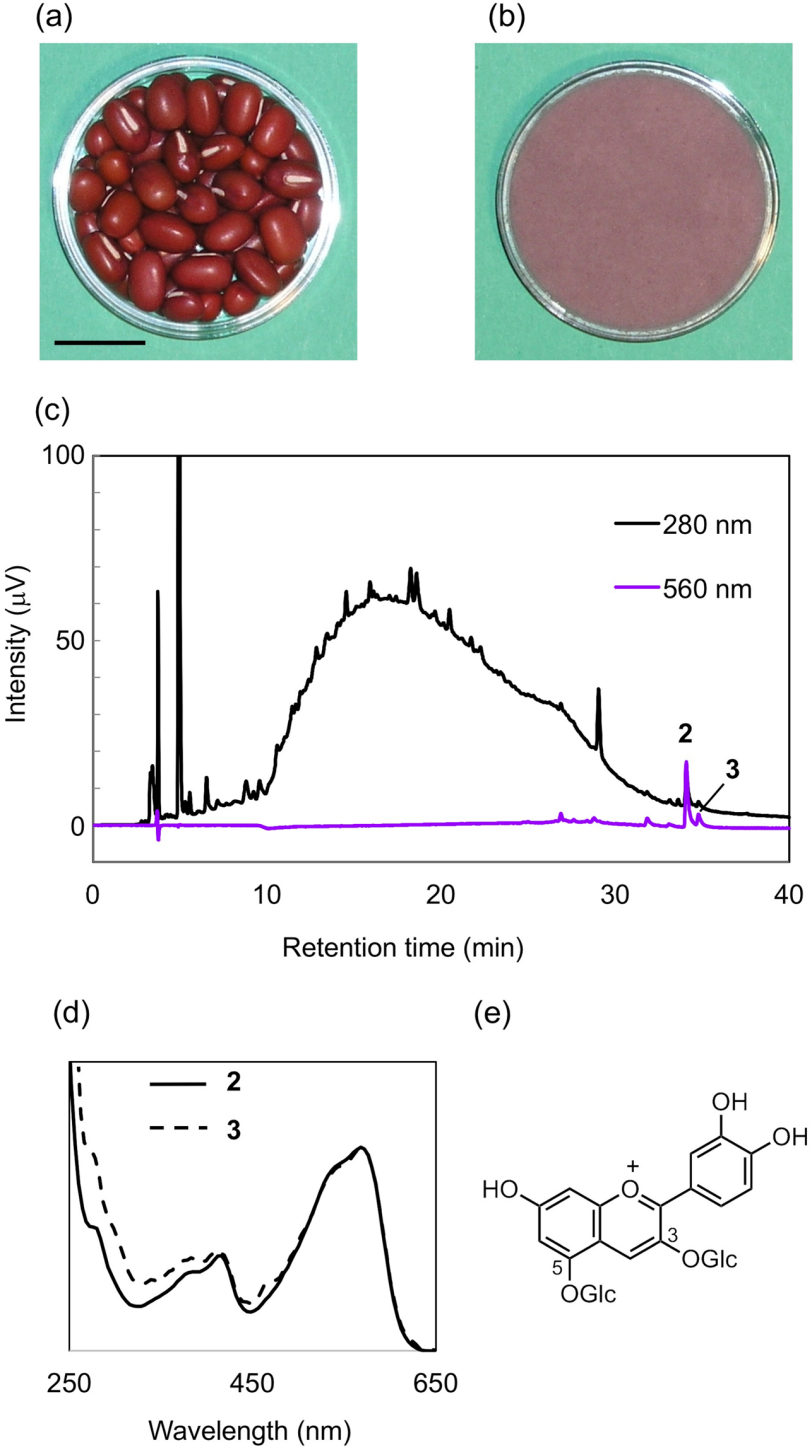
Red adzuki beans and their chromatographic and spectral profiles. (**a**) Photos of red adzuki beans (scale bar: 10 mm) and (**b**) the purple an-paste. (**c**) HPLC chromatogram of the red adzuki bean seed-coat extract. (**d**) UV-Vis spectra of **2** and **3** obtained by 3D-detection. (**e**) The structure of cyanin.

The small red bean, *Vigna angularis*, originated in East Asia but is known worldwide today by its Japanese name, “adzuki”^1,2^. Although the color of the seed coat varies from white, brown, or red to black, the most common seed coat color is red (Fig. 1a)^3^. In Japan, the adzuki bean occupies a very special position among beans in terms of food culture and agricultural history; food products prepared with adzuki beans, including red rice, and Japanese sweets, are eaten at special ceremonial or celebratory occasions such as weddings, the birth of a child, festivals, and New Year’s Day, because the red color of the seed coat is associated with good fortune^4–6^.

Adzuki beans are primarily used to produce “an-paste” (Fig. 1b), which is a conglomeration of “an-particles” derived from each grain cell. The size of an an-particle is approximately 100 μm i.d., with an outer layer composed of the thermally denatured cell wall and cell membrane containing membrane proteins, and an interior filled with gelatinized starch granules^5–9^. The grain cells are colorless at the start of processing, but as the adzuki beans are boiled, the seed-coat pigments are transferred to the outer layer to produce the purple-colored an-paste^6–9^. Thus, the seed-coat color is correlated with the hue of the an-paste and the processing conditions (including both the water composition and cooking pan materials as very important factors)^6–9^. An-pastes are evaluated by their purple coloration (Fig. 1b), and their quality has been judged on the basis of their color^6,8^. However, although many studies on the seed-coat pigments of red adzuki beans have been reported, none have clarified their structures or chemical properties.

In 1934, Kuroda and Wada reported that water-insoluble pigment and brown tannin components co-existed in red adzuki beans, but the pigment was very difficult to purify^10^. In 1966, Sasanuma *et al*. isolated delphinidin 3-*O*-glucoside from the black adzuki bean^11^. Ishikura *et al*. identified 3-*O*-glucosides of cyanidin and delphinidin from the black matpe bean, *V. mungo*^12^. Many subsequent trials to elucidate the seed-coat pigment from red adzuki beans were unsuccessful. However, based on the similarity of the adzuki bean color with that of the red kidney bean (*Phaseolus vulgaris* cv. Kintoki), the pigment in adzuki has been considered to be an anthocyanin.

We have been interested in the seed-coat anthocyanins of various colored beans and tried to isolate anthocyanin from red adzuki beans. By extraction of the peeled seed-coat of the red adzuki bean with a 50% aqueous acetonitrile (CH_3_CN) containing 3% trifluoroacetic acid (TFA) followed by chromatographic purification, we obtained cyanidin 3,5-di-*O*-glucoside (cyanin, **1**, Fig. 1e)^13^. However, the content of **1** was very low, less than 0.01 mg per 1 g dried peel^13^. Furthermore, since this simple anthocyanin is unstable under weakly acidic-to-neutral conditions and is easily degraded by heating, it is unlikely that either the red color of the seed coat or the purple color of the an-paste is due to **1**.

A comprehensive survey of seed-coat components reveals an existence of a large number of procyanidins, a mixture of 3-flavanol polymers, flavonols, and catechin-glucosides^14^. These polyphenols complicate the isolation of pigments. However, with further investigation, we have found two purple pigments that are eluted much later than the usual anthocyanins by reversed-phase HPLC analysis (Fig. 1c). Herein, we describe these purple pigments, catechinopyranocyanidins A and B, from red adzuki beans as well as their chemical structures and physicochemical properties. The pigments are highly oxidized polyphenolic compounds that contain a new pyrano ring-system of catechin and cyanidin, and exhibit unique characteristics. Similar purple pigments were reported recently, but no conclusive evidence in the determination of their condensed ring systems and absolute configuration were reported and the structures differed from our results^15^. We performed not only NMR analysis, but also derivatizations and computer-assisted calculations, for structure determination. Based on the obtained data, we report these new structures with solid chemical supporting evidence.

## Results and Discussion

### Isolation of catechinopyranocyanidins A (2) and B (3)

Small red beans (*V. angularis* cv. Erimoshozu) were soaked in water and their seed-coat were peeled. After freezing, the seed-coats were extracted with 3% TFA in 50% aq. CH_3_CN, and the extracts were analyzed by HPLC using 3D-detection. In the chromatogram of the extract (Fig. 1c), two pigment peaks, **2** and **3**, were detected. The UV-Vis spectra of **2** and **3** obtained with 3D-detection HPLC eluted with a strong acidic condition showed *λ*_vismax_ values around 560 nm, which are clearly different from that of cyanin **1** (*λ*_vismax_: 520 nm) and indicated that, even under strongly acidic conditions, the pigments exhibited a purple color (Fig. 1d). This strongly suggests that **2** and **3** should have a different chromophore and chemical properties compared to those of anthocyanins^16^. In addition to the pigments, the HPLC chromatogram revealed the presence of numerous polyphenolic compounds in the extract, necessitating the removal of the non-pigment components for the isolation of **2** and **3**. As a further complication, we found during preliminary experiments that **2** and **3** were labile to light; even in dim light such as indoor fluorescent light, the pigment solution became colorless within a day. Therefore, all the experiments, including extraction, evaporation, and purification procedures, were performed under dark conditions.

**Figure 2:**
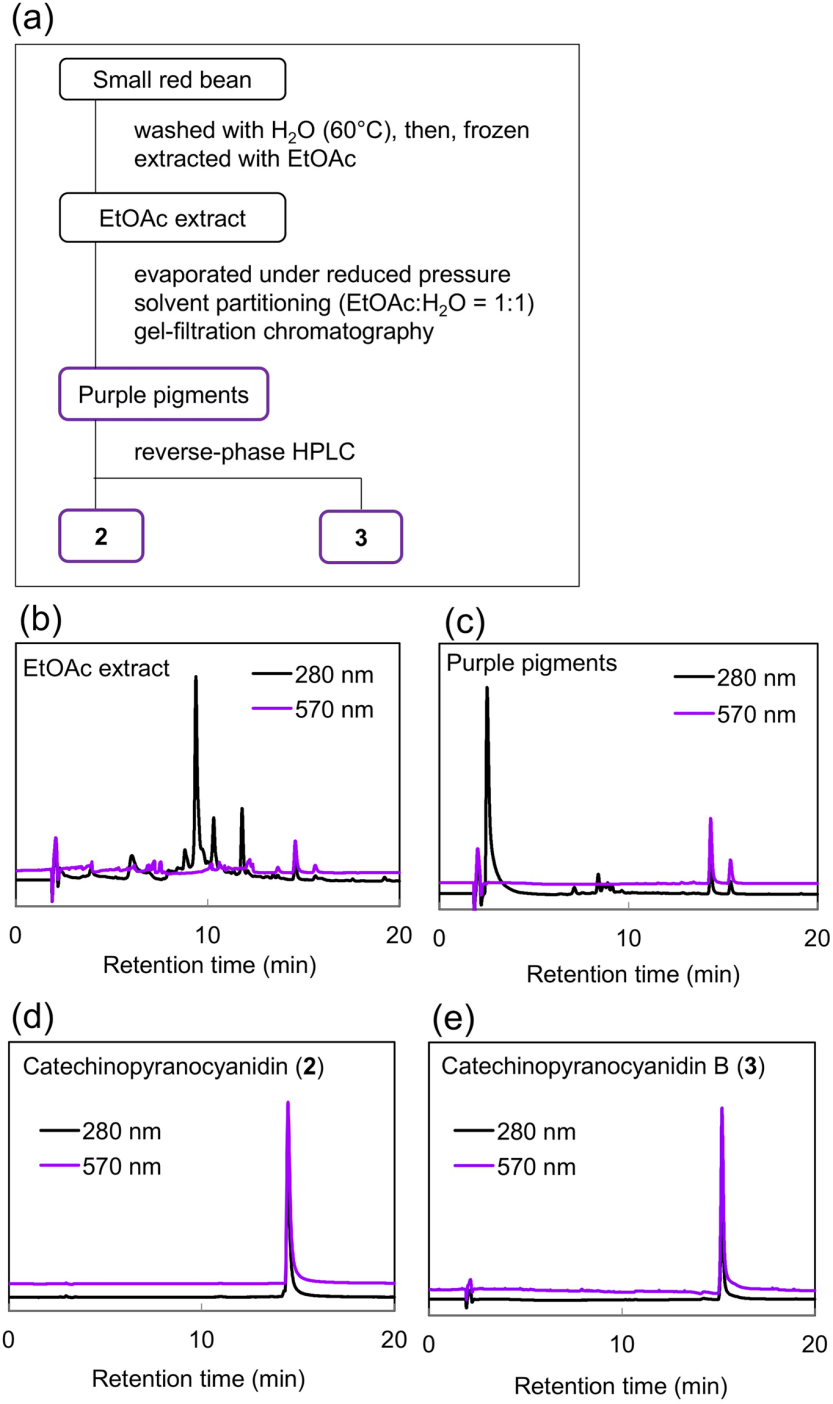
Purification of catechinopyranocyanidins from adzuki beans. (**a**) Purification procedure. (**b**) HPLC chromatograms of the EtOAc extract, (**c**) purple pigments, (**d**) catechinopyranocyanidin A (**2**), and (**e**) catechinopyranocyanidin B (**3**).

**Figure 3:**
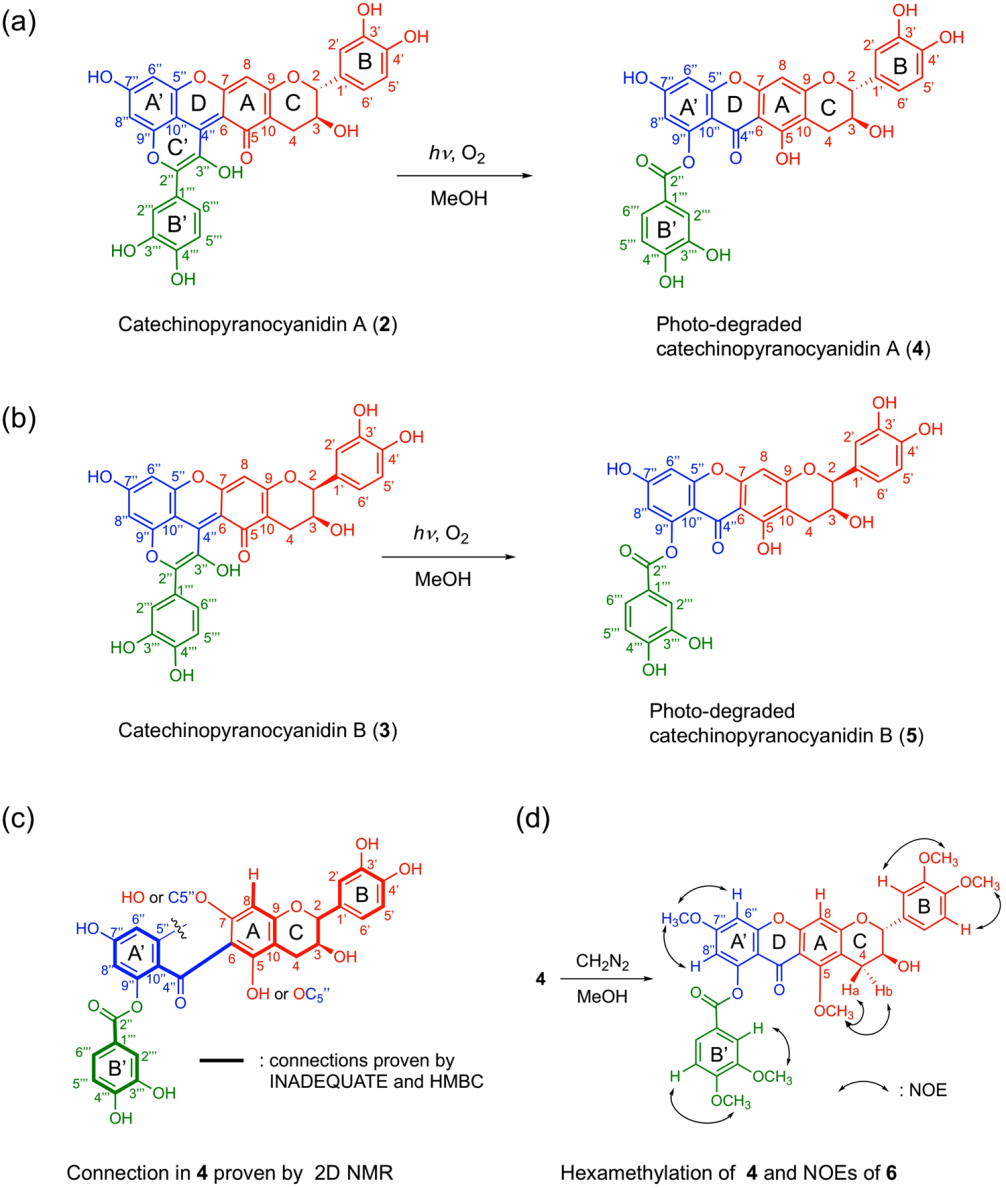
Structures of catechinopyranocyanidins and their reaction products. (**a**) Structure of catechinopyranocyanidin A (**2**) and its colorless photo-degradation product (**4**). (**b**) Structure of catechinopyranocyanidin B (**3**) and its colorless photo-degradation product (**5**). (**c**) INADEQUATE and HMBC correlations obtained by 2D-NMR experiments of **4**. (**d**) Hexamethylation of **4** to give **6** and resultant NOEs.

Preparatively, compounds **2** and **3** were purified according to the procedure shown in Fig. 2a. Red adzuki beans (*V. angularis* cv. Erimoshozu) were washed with hot water repeatedly and frozen at −30 °C overnight. The frozen beans were extracted with ethyl acetate (EtOAc) for 24 h, then, the solvent was evaporated under reduced pressure. To the concentrated extract, the same volume of water was added, mixed vigorously, and then partitioned. The EtOAc layer was concentrated to give the crude pigment (Fig. 2b), which was purified by gel-filtration column chromatography (Fig. 2c). Repeated reversed-phase HPLC afforded pure **2** (Fig. 2d) and **3** (Fig. 2e). From 20 Kg of beans 23 mg of **2** and 2.3 mg of **3** were obtained, respectively.

### Molecular formulas and partial structure of catechinopyranocyanidins A (2) and B (3)

Electrospray-ionization time-of-flight mass spectrometry (ESI-TOF MS) analysis with positive-mode detection of **2** gave a protonated molecular ion peak at *m/z* = 557 [M + H]^+^ and a sodium adduct ion at *m/z* = 579 [M + Na]^+^, indicating that the molecular weight of **2** is 556. Mass analysis of **3** gave the same ions observed for **2** at *m/z* = 557 [M + H]^+^ and 579 [M + Na]^+^, also indicating a molecular weight for **3** of 556. By high-resolution mass spectrometry (HR-MS) with positive-mode detection (found: 557.1071, calcd. for C_30_H_21_O_11_: 557.1078, Fig. S1-1), the molecular formula of **2** was determined as C_30_H_20_O_11_. Compound **3** has the same formula as **2** ([M + H]^+^ found: 557.1073, Fig. S9-1), indicating that **2** and **3** are structural isomers.

Next, various 1D and 2D NMR measurements of **2** were carried out (Fig. S3-8-4). From the ^1^H NMR (Fig. S3) and correlation spectroscopy (COSY) analyses (Fig. S5-1,2), signals attributable to the B-ring of a cyanidin moiety and the B-C ring of a catechin residue were observed (Table 1). The stereostructure of the catechin part (C2 and C3 in ring C) was determined to be 2,3-*trans* by the *J* value (8.5 Hz). However, because **2** has 18 quaternary carbons and the ring system is highly oxidized, Heteronuclear multiple-bond correlation spectroscopy (HMBC) measurements could not unambiguously determine the connectivity and structure of **2**. The ^1^H NMR signals at lower field in **3** are similar to those of **2**, but the signals at higher field are obviously different (Fig. S11). From the very small *J* values for 2 H and 3 H (Table 1), it was suggested that the configuration of the catechin part of **3** likely adopted a 2,3-*cis* relationship, although the chromophore was as the same as that in **2** (Fig. 1d).

**Table 1:** ^1^H- and ^13^C -NMR assignment of catechinopyranocyanidins A (**2**) and B (**3**).

	2 (CD_3_OD)				2 (DMSO-*d*_6_)				3 (CD_3_OD)			
	^1^H			^13^C	^1^H			^13^C	^1^H			^13^C
	* δ* (ppm)	multiplicity, *J* (Hz)		* δ* (ppm)	* δ* (ppm)		multiplicity, *J* (Hz)	* δ* (ppm)	* δ* (ppm)		multiplicity, *J* (Hz)	* δ* (ppm)
2	4.78	d	7.5	83.6	4.82	d	6.5	81.8	4.78	brs		80.8
3	4.07	ddd	8.0, 7.5, 5.0	68.4	3.99	ddd	8.0, 7.5, 6.5	65.8	4.23	brt	4.0	67.1
4a(ax.)	2.57	dd	16.0, 8.0	29.8	2.48	dd	17.0, 8.0	27.3	2.79	dd	16.0, 4.0	30.1
4b(eq.)	2.87	dd	16.0, 5.0		2.64	dd	17.0, 6.0		2.84	dd	16.0, 4.0	
5				168.3				167.5				168.6
6				104.9				103.4				105.0
7				155.8				153.7				155.5
8	6.14	s		93.2	6.17	s		92.8	6.10	d	2.0	93.5
9				164.0				161.9				164.5
10				107.8				106.3				107.0
1′				131.3				129.6				131.6
2′	6.87	d	2.0	115.1	6.73	d	2.0	115.3	6.99	d	1.5	115.3
3′				146.5				145.0				146.0
4′				146.4				145.1				146.0
5′	6.81	d	8.5	116.2	6.72	d	8.5	114.1	6.79	d	8.0	115.9
6′	6.76	dd	8.5, 2.0	120.0	6.61	dd	8.0, 2.0	118.0	6.81	dd	8.0, 1.5	119.5
2″				150.7				145.1				154.5
3″				143.0				144.1				142.9
4″				144.5				141.3				144.5
5″				152.1				150.2				152.0
6″	6.77	d	2.0	99.7	6.71	d	2.0	98.6	6.49	d	2.0	99.63
7″				164.0				162.2				163.9
8″	6.58	d	2.0	97.4	6.87	d	2.0	96.4	6.68	d	2.0	97.4
9″				152.8				150.9				152.7
10″				106.9				105.2				106.8
1″′				124.8				123.0				124.83
2″′	8.09	d	2.0	117.5	8.16	d	2.0	116.5	8.03	d	2.0	117.5
3″′				146.2				149.5				146.1
4″′				154.8				152.1				150.7
5″′	6.90	d	8.5	116.3	6.92	d	8.5	115.8	6.89	d	8.5	116.3
6″′	8.02	dd	8.5, 2.0	124.5	8.03	dd	8.5, 2.0	122.6	7.99	dd	8.5, 2.0	124.5

### Photo-degradation of catechinopyranocyanidins A (2) and B (3)

The various 1D and 2D NMR experiments did not enable the complete structural determination of **2**; therefore, we changed our strategy by preparing its photo-degradation product to obtain structural information on the skeleton of the purple pigment. Since we clarified that purple pigments **2** and **3** were labile to light early in this study, a methanol (MeOH) solution of **2** was exposed to ambient light in an incubator. A solution of **2** (0.05 mM in MeOH) was poured into a quartz cuvette and stored in an incubator at 25 °C. The solution in the cuvette was irradiated by fluorescent light and the degradation reaction was analyzed by HPLC (Fig. S17). After 1 h, a new colorless peak appeared, and after 8 h, **2** was completely consumed. In the HPLC chromatogram, no other major peaks except **4** were observed; therefore, we conclude that **2** was converted to **4** almost quantitatively (Fig. 3a). Using the same conditions, **3** was converted to colorless **5** (Fig. 3b). In a large-scale experiment for structural analysis, a mixture of **2** and **3** in MeOH was irradiated to give a mixture of **4** and **5**; then, each compound was purified by HPLC.

At present, the mechanism of the photo-degradation is not clear, but, it was clarified that both O_2_ and light were essential. The decomposition does not occur in dark condition. Under argon atmosphere the pigments were stable even in light condition.

### Structure determination of photo-degraded catechinopyranocyanidin A (4)

The positive-mode HR-ESI-TOF MS analysis of **4** gave a protonated molecular ion peak at *m/z* = 561.1026 [M + H]^+^, (calcd for C_29_H_21_O_12_ [M + H]^+^: 561.1028, Fig. S18) indicating that the molecular formula of **4** is C_29_H_20_O_12_. During MS analysis, a fragment ion peak was also detected at *m/z* = 425.0868, attributable to the loss of a 3,4-dihydroxybenzoic acid unit. HR-MS analysis of **5** with positive-mode detection gave a molecular ion peak at *m/z* = 561.1025 [M + H]^+^; therefore, **5** has the same molecular formula as **4** (Fig. S28).

Using 10 mg of **4** in CD_3_OD, we measured and analyzed its ^1^H- and ^13^C-NMR, COSY, nuclear Overhauser effect spectroscopy (NOESY), heteronuclear single-quantum correlation spectroscopy (HSQC), HMBC, and incredible natural-abundance double-quantum transfer experiment (INADEQUATE) spectra (Table 2, Fig. S20-26-3). In the ^1^H-NMR spectrum, the signals attributable to the B-C rings of the catechin residue (indicated in red in Fig. 3c) remained. In addition, signals attributable to a 1,2,3,5-tetrasubstituted benzene ring (6.56 ppm, d, 2.0 Hz; 6.71 ppm, d, 2.0 Hz; indicated in blue in Fig. 3c) and signals to a 1,3,4-trisubstituted benzene ring (6.92 ppm, d, 8.5 Hz; 7.63 ppm, d, 2.0 Hz; 7.65 ppm, dd, 8.5, 2.0 Hz, which was suggested by the mass spectrum and is indicated in green in Fig. 3c) were observed. The signal attributable to H6 of A-ring in **4** was not observed indicating that 6 position of catechin parts (red part in Fig. 3c) should connect to other part. Analysis of the HMBC (Fig. S25-1-4) and INADEQUATE correlations (Fig. S26-1-3) suggested that C5″ is likely connected to the oxygen atoms at C5 or C7 of ring A in the catechin part (Fig. 3c). However, the connection could not be determined unambiguously, because no HMBC correlation was observed to C4″ carbonyl carbon (Fig. S25-4). Combining these results, we proposed two candidate structures in which a new pyrano ring-system (D-ring) connects at the 5,6- or 6,7-position of the catechin unit (Fig. S27).

**Table 2:** ^1^H- and ^13^C-NMR assignment of photo-degraded catechipyranocyanidins A (**4**) and B (**5**).

	4 (CD_3_OD)				5 (CD_3_OD)			
	^1^H			^13^C	^1^H			^13^C
	*δ* (ppm)	multiplicity, *J* (Hz)		*δ* (ppm)	*δ* (ppm)	multiplicity, *J* (Hz)		*δ* (ppm)
2	4.83	d	6.5	83.6	5.02	brs	4.0	81.1
3	4.08	ddd	8.0, 6.5, 5.0	67.6	4.26	brt	4.0, 4.0, 4.0	66.4
4a (ax.)	2.61	dd	16.0, 8.0	27.0	2.87	dd	16.0, 4.0	28.5
4b (eq.)	2.87	dd	16.0, 5.0		2.92	dd	16.0, 4.0	
5				161.8				162.3
6				103.7				104.1
7				156.3				156.3
8	6.37	s		94.8	6.43	d		95.0
9				162.2				163.0
10				104.6				103.9
1′				131.1				131.2
2′	6.90	d	2.0	115.0	6.98	d	1.5	115.3
3′				146.3				146.1
4′				146.5				146.3
5′	6.83	d	8.5,	116.2	6.78	d	8.0	116.0
6′	6.73	dd	8.5, 2.0	120.0	6.79	dd	8.0, 1.5	119.5
2″				167.0				167.0
3″								
4″				180.4				180.6
5″				153.2				153.3
6″	6.56	d	2.0	109.2	6.49	d	2.0	101.6
7″				165.1				165.2
8″	6.71	d	2.0	101.6	6.73	d	2.0	109.2
9″				160.1				160.2
10″				108.1				108.2
$1\prime\prime\prime$				122.2				122.2
$2\prime\prime\prime$	7.63	d	2.0	118.2	7.61	d	2.0	118.3
$3\prime\prime\prime$				146.2				146.1
$4\prime\prime\prime$				152.3				152.3
$5\prime\prime\prime$	6.92	d	8.5	116.0	6.91	d	8.5	116.0
$6\prime\prime\prime$	7.65	dd	8.5, 2.0	124.8	7.63	dd	8.5, 2.0	124.8

For further structural determination studies, **4** was transformed to hexamethylated compound **6** by treatment with diazomethane (Fig. 3d); then, **6** was subjected to HR-MS and various NMR experiments (Table S1, Fig. S36-38-2). NOESY studies to determine nuclear Overhauser effects (NOEs) between the newly installed methyl groups and molecular framework protons were carried out, with the results shown in Table S1 and Fig. 3d. NOEs were observed between the methylene protons at C4 (4 Ha and 4Hb) and the methyl signal at 3.82 ppm in **6** (Fig. 3d, Table S1, Fig. S38-2), but no NOE was observed between methyl protons and the signal of H8 of the A-ring of the catechin part. Other NOEs were observed between methyl protons and protons in the B-, A′-, and B′-rings (Fig. 3d). Combining these results, the structure of **6** was determined unambiguously as shown in Fig. 3d. Thus, it was confirmed that the pyrano ring-system (D-ring) in **4** exists at the 6,7-position of the catechin segment. At this stage, the absolute configurations of C2 and C3 in **2** were not clarified, but their relative configuration was assigned as *trans*. By similar MS and NMR experiments, the structure of **5** was determined with a relative *cis*-configuration at C2 and C3 (Table 2, Fig. S28-35-2).

### Structure of catechinopyranocyanidins A (2) and B (3)

Using the structural information for **4** and **5**, further structural analyses of **2** and **3** were undertaken. MS/MS analysis of the molecular ion at *m/z* = 557.11 of **2** gave a fragment ion peak at *m/z* = 405.06, which is identified as an ion derived from the retro-Diels-Alder reaction of **2** (Fig. S1-2). MS/MS of **3** (from *m/z* = 557.11) also gave the same fragment ion peak (*m/z* = 405.06, Fig. S9-2); these results confirm the presence of the catechin unit in **2** and **3**.

Next, we re-examined all the NMR spectral data of **2** for the confirmation of the structure including the position of pyrano ring-system as 6,7-position of the ring-A of catechin segment. Analysis of the HSQC (Fig. S7-1-3) and HMBC data for **2** (Fig. S8-1-4) indicated the presence of the same partial structures (catechin part indicated in red in Fig. 3c) as observed in **4**. By NOEs observed between H8″ (6.58 ppm, d, 2.0 Hz) and H2‴ (8.09 ppm, d, 2.0 Hz), and H8″ and H6‴ (8.02 ppm, dd, 8.5, 2.0 Hz) (Fig. S6-1,2), and HMBC correlations (Fig. S8-4), the existence of cyanidin part in **2** was clarified. From the structure of **4** and **6**, pyran-ring in **2** exists between C6-C7 of ring-A. Therefore, the connectivity of the catechin and cyanidin in **2** was clarified unambiguously (Fig. 3a).

From H4b (H4eq.), HMBC correlations are observed to C5 (168.3 ppm in CD_3_OD, Fig. S8-2-4), whose chemical shift seems very low and an unusual value for a phenolic carbon atom, instead, is presumed to be a carbonyl carbon. Since the molecular formula, IR spectrum (peak at 1681 cm^−1^, Fig. S2) and ^13^C NMR (168.3 ppm, Table 1, Fig. S4) of **2** indicated the existence of carbonyl group, we tried to determine the position of carbonyl residue. In general, quinonoidal base structure of the anthocyanidin chromophore exhibits a purple color^16^, thus, we first considered that the 7″- or 4‴-position of cyanidin part might be the carbonyl carbon. However, from the ^13^C-NMR assignments (Table 1), the chemical shifts of the signals for C-7″ and C-4‴ in CD_3_OD are 164.0 and 154.8 ppm, respectively, making it difficult to assign the carbonyl carbon. Compound **2** has an extended conjugated system compared with that of an isolated cyanidin, therefore, one more tautomeric structure can be achieved with a carbonyl at the 5-position. As described previously, the chemical shift of C5 is 168.3 ppm (Table 1), indicating strongly that C5 should be a carbonyl carbon.

To give a solid proof for this assignment, we calculated the ^13^C NMR chemical shifts using density functional theory (DFT), employing Spartan’16 software^17–19^. Three tautomeric structures of **2**—the 5-keto, 7″-keto, and 4‴-keto forms—were subjected to the calculations (Fig. S39). After the conformational search was performed, the obtained conformers were refined and optimized with ωB97X-D/6-31 G* model. The ^13^C chemical shifts of each elemental conformer were calculated with the same model, and those were corrected on the Boltzmann weighting calculated with a single point energy calculation employing ωB97X-V/6-311 + G(2df, 2p)[6-311 + G*]//ωB97X-D/6-31 G* model to give theoretical shifts. As shown in Table S2, the obtained chemical shift values of C5, C7″, and C4‴ in the 5-keto form agree with the experimental data, whereas the other forms show considerable divergence. The 5-keto form affords the smallest root mean square deviation (RMS), 2.98 ppm (Table S2), which is around the accuracy level in this method^18^. In contrast, the RMS values of C7″-keto and C4‴-keto forms are 6.06 and 8.16 ppm, respectively, (Table S2), which were considerably above the allowable level. Thus, we concluded the structure of **2** as the 5-keto tautomer (Fig. 3a).

Since the NMR and MS spectral profiles of **3** resemble those of **2**, except for the *J* values of H2 and H3, it is presumed that **3** is a stereoisomer of **2**, wherein the stereostructure at the 2,3-position of **3** has the *cis* configuration. The coupling constants, *J*_2,3_, *J*_3,4a_ and *J*_3,4b_, in Table 1 have a good similarity of those of (−)-epicatechin and (+)-epicatechin^20,21^. Using the same calculation procedure, the ^13^C chemical shifts of **3** were calculated to reveal that **3** has the same planar structure except for the stereochemistry at C2 and C3 (Table S3). From these considerations, the relative structures of **2–5** were successfully determined (Fig. 3a,b).

### Absolute configuration of catechinopyranocyanidin A (2) and B (3)

Recently, the remarkable progress in theoretical calculations has enabled the use of computed electronic circular dichroism (ECD) in the determination of the absolute configuration of natural products^18,19,22^. We applied this methodology to determine the absolute configurations of C2 and C3 in **2** and **3**. ECD curves were computed using time-dependent density functional theory (TD-DFT) calculations (the computational details are summarized in the Methods and Supporting Information) and compared with the experimental data. A simple strategy may be to compute ECD curves by considering a Boltzmann-weighted series of conformers^17–19^. However, many local minima were expected for **2** and **3** based on the previous conformational search experiments for ^13^C chemical shift estimation. Therefore, this simple strategy may not be suitable.

**Figure 4:**
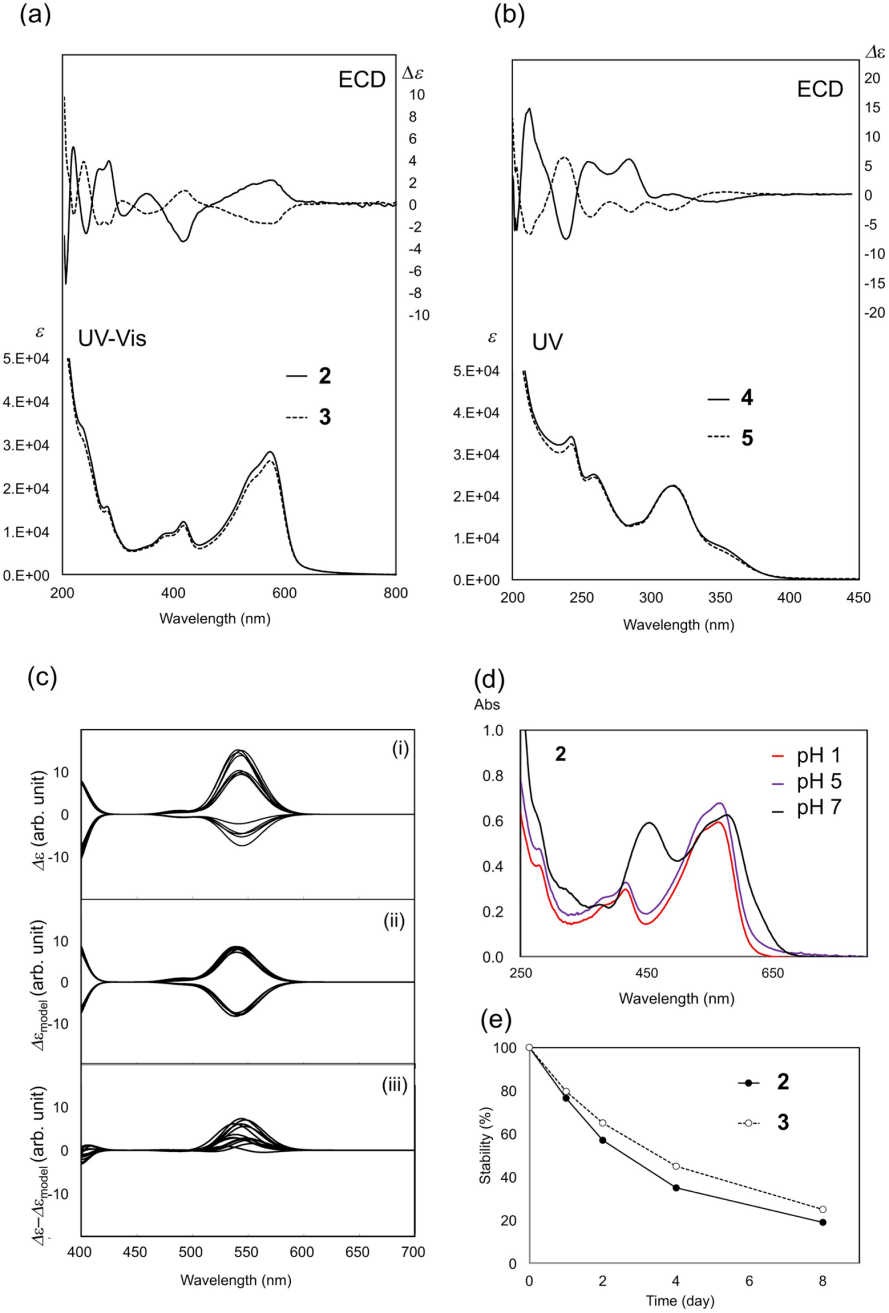
Spectroscopic characterization of catechinopyranocyanidins A (**2**) and B (**3**). (**a**) UV-Vis and ECD spectra of **2** and **3**. (**b**) UV and ECD spectra of **4** and **5**. (**c**) Computed ECD curves for 13 conformers of **2** (Δ*ε*, (i)), the corresponding model (Δ*ε*_model_, (ii)), and their differences (Δ*ε*–*ε*_model_, (iii)). (**d**) Vis spectra of **2** at various pH buffer solutions. (**e**) Stability of **2** and **3** at pH 5.

It is expected that the Cotton effect in the visible region is due to the conjugated chromophore in **2** and **3**. To clarify this, we calculated the ECDs of a non-chiral model compound in which both the 3,4-dihydroxyphenyl group at C2 and hydroxyl group at C3 in C-ring were replaced by hydrogen atom (Fig. S40). The obtained ECDs of the 13 conformers of the model molecule (2-CH_2_ and 3-CH_2_ compounds) are shown in Fig. 4c(ii). The appearance of both signs is ascribed to the helicity around C6 for two reasons; first, the positive (negative) dihedral angle for 10″−4″−6−5 was found to correspond to the positive (negative) rotatory strength, and second, this result holds true for the counterpart model molecule (Table S4). However, this helicity may not be a key factor by which to characterize the ECD in the visible region of **2** and **3**, because two structures with the opposite helicity around C6 are unlikely to produce distinctive energy differences, and thus, the positive and negative contributions are expected to be mostly canceled by the Boltzmann averaging of various conformers.

Thus, to highlight the effect of the chiral configuration on the ECD, we planned a strategy to visualize the effect by the subtraction method. The computed Δ*ε* for the 13 optimized conformers of **2** with 2*R*, 3*S* are displayed in Fig. 4c(i), and from the ECDs of each conformer, the ECDs of the model compound were subtracted. The results, shown in Fig. 4c(iii) as Δ*ε* − Δ*ε*_model_, indicate that the (*R*, *S*) configuration at the C2 and C3 positions has a positive contribution in the ECD at visible region, irrespective of the conformation (Fig. 4c(iii)). Therefore, the contribution from the asymmetric centers at C2 and C3 is a key factor in characterizing the ECD in this region. Similarly, for **3**, both signs at visible region are observed in the Δ*ε*_model_ (Fig. S41(ii)), which are again expected to mostly cancelled by the Boltzmann averaging of the various conformers. In this case, the negative peaks at visible region are observed in Δ*ε* − Δ*ε*_model_ (Fig. S41(iii)), indicating that the 2*S*, 3*S* configuration has a negative contribution irrespective of conformation and is a key factor in characterizing the experimentally observed negative ECD in this region (Fig. 4a). From these results, it is reasonable to surmise that the positive and negative peaks in the experimental ECDs in the visible region can be ascribed to the 2*R*, 3*S* and 2*S*, 3*S* configurations at the C2 and C3 positions for **2** and **3**, respectively.

### Purple color development of catechinopyranocyanidin A (2) and B (3)

Since compound **2** is water-insoluble, **2** was dissolved in 50% MeOH solutions containing buffers of varying pH, and their UV-Vis absorption spectra were recorded (Fig. 4d). At pH 1.0 and pH 5.0, **2** (50 μM) shows a beautiful purple color, and the spectra are nearly the same, with *λ*_vismax_ at 560 nm (pH 1.0) and 563 nm (pH 5.0). At pH 7.0, an additional absorption peak at 440 nm appeared and the color of the solution becomes redder. Compound **3** in the same buffered solutions gave the similar color with those of **2** (Fig. S42). The stability of the color at pH 5.0 was probed in Fig. 4e. Under dark conditions, **2** was very stable and the color was retained after 8 days at rt. The same stability was observed with **3**. In contrast to **2** and **3**, simple anthocyanins and anthocyanidins show different color profiles at pH 1 and 5; at pH 1, the chromophores adopt the flavylium cation form with a red color, and at pH 5, they take a quinonoidal base form with a purple color^16,23^. In addition, at pH 5, simple anthocyanins without aromatic acyl moieties are easily hydrated to give colorless pseudobase within a couple of minutes. Thus, the chromophore of catechinopyranocyanidins display much different characteristics compared to those of anthocyanins.

Recently, several pyranoanthocyanins, which have a pyran-ring between C5 and C4 of the anthocyanidin chromophore, have been identified in wine^24–28^. These compounds are considered to be produced during fermentation by reaction with acetaldehyde and/or other aldehydes, ethanol, and organic acids^24^. Most pyranoanthocyanins exhibit an orange-red color and are very stable in weakly acidic-to-neutral conditions^25–27^. This is because that the hydration reaction to anthocyanidin chromophore is generally occur at C2 and C4 and the substitution at this position inhibit hydration reaction. The stability of catechinopyranocyanidin A (**2**) and B (**3**) may also be due to substitution at the C4″ position. The purple color development may be due to the extended conjugated system in **2** and **3**, as found in blue pyranoanthocyanins in port wine^27,28^. The reason why the same color may be observed under strongly acidic-to-weakly acidic conditions might be because the p*K*a value between flavylium cation and quinonoidalbase is less than 1, although the p*K*_a_ value of simple anthocyanins is around 4.

In conclusion, we isolated two hydrophobic purple pigments, catechinopyranocyanidins A (**2**) and B (**3**), from the seed coats of red adzuki beans, and determined the structure. Compounds **2** and **3** have a new condensed ring system composed of catechin and cyanidin skeletons with a new pyrano ring at C6 and C7 of the catechin segment. The structure of **2** and **3** is differed from vignacyanidin with condensed position, existence of carbonyl group and charge (Fig. S43)^15^. We clarified that the seed-coat pigments in red adzuki beans are not anthocyanins, but rather, hydrophobic compounds, which reasonably explains the processing procedure for coloring “an-paste” by boiling in water and washing repeatedly with water. The photo-degradation of **2** and **3** under dim light is very curious and interesting phenomenon, also. At present, we can only predict the mechanism as follows: radical generation by light irradiation, reaction of the radical with oxygen, then, elimination of carbon monoxide to give colorless compound (Fig. S44). Further studies of clarification of the mechanisms of this photo-degradation, biosynthetic pathway for generation of the pigments in the seed coat and the differences in an-paste coloring according to modification of the food processing procedure are in progress.

## Methods

### Plant materials

Red adzuki beans, *V. angularis* cv. Erimoshozu, were purchased from Hokkaido Research Organization, Agricultural Rerserach Department Tokachi Agricultural Experiment Station and Hokuren.

### General procedure

Infrared (IR) spectra were recorded on a JASCO FT/IR 6100 spectrometer. UV-VIS spectra were recorded on a JASCO V-560 spectrometer. ECD spectra were measured with a JASCO J-720 spectrometer. ESI-TOF-MS and HR-MS were recorded on a Bruker microTOF-QII (ESI) spectrometer. NMR spectra were obtained with a JEOL JNM-ECA-500 (^1^H: 500 MHz and ^13^C: 125 MHz) and Bruker Daltonics AVANCE III HD 600 with a TCI cryoprobe and BBO cryoprobe (^1^H: 600 MHz and ^13^C: 150 MHz) in a 5-mm i.d. tube at variable temperatures using CD_3_OD or DMSO-*d*_6_ as solvent. Chemical shifts were recorded as parts per million (ppm) with the CD_2_HOD or C_2_D_5_HOS resonance used as the standard. In the 2D-NMR experiments, the standard pulse sequences of the JEOL and Bruker instruments were used. Analytical HPLC was performed using a JASCO HPLC system composed with two PU-1585 pumps, a HG-1580-32 mixer, a DG-1580-53 degasser, a MD-1515 detector, and CO-1565 column oven and the system was controlled using ChromNAV ver 2 application. Reversed phase columns (Develosil ODS-HG-5, 2.0 mm i.d. × 250 mm, and Develosil RPAQUEOUS-AR-3, 2.0 mm i.d. × 150 mm, Nomura Chemical) were eluted with a linear gradient elution from 10% to 90% aq. CH_3_CN solution containing 0.5% TFA at 40 °C. Preparative HPLC was carried out using a JASCO preparative HPLC system composed with a 880-PR pump, a UV-970 detector, a RC-250 recorder and a thermostatic oven (Jr-80, Taiyo Kagaku). Develosil ODS-HG-5 (4.6 mm i.d. × 250 mm and 20 mm i.d. × 250 mm, Nomura Chemical) and RPAQUEOUS-AR-5 (20 mm i.d. × 250 mm, Nomura Chemical) were eluted with a stepwise gradient elution from 10% to 40% aq. CH_3_CN containing 0.5% TFA at 40 °C.

### Isolation of purple pigments 2 and 3

Red adzuki beans (10.0 kg) were washed several times with water (60 °C), after which the beans were frozen at −30 °C overnight. To the frozen beans, 12.0 L EtOAc was added, and the mixture was let stand at rt for 24 h, protected from light. The extract was evaporated under reduced pressure to 1.0 L, and then was mixed vigorously with EtOAc (1.0 L). After partitioning, the EtOAc layer was collected and concentrated *in vacuo* to obtain the crude pigment (2.2 g). The crude pigments (6.9 g) obtained from several runs were combined and purified by gel-filtration column chromatography. A column (60 mm i.d. × 430 mm) filled with TOYOPEARL HW-40C resin (Tosoh Bioscience) was washed with methanol (MeOH) before applying a MeOH solution of the pigment. After again washing with MeOH, the column was subjected to stepwise elution with 10% to 70% acetone in MeOH (400 mL each), collecting each fraction separately. After HPLC analysis, fractions 2–4 were combined and concentrated *in vacuo* to give a mixture of **2** and **3** (670 mg). Further purification was performed *via* preparative HPLC (Develosil ODS-HG and RPAQUEOUS-AR-5, 20 mm i.d. × 250 mm). A DMSO solution of the mixture of **2** and **3** (4.7 mg) was injected into the column which was pre-washed with 30% aq. CH_3_CN containing 0.5% TFA, and then eluted. This procedure was carried out repeatedly, eventually producing pure **2** (23 mg) and **3** (2.0 mg) as dark purple masses.

### Photo degradation of 2

Pure **2** (30 μg) was dissolved in MeOH (1.08 mL) to prepare a solution with a 50 μM concentration. The solution was poured into a quartz cuvette (path length: 10 mm), placed in a plant growth chamber (MLR-350, Sanyo), and irradiated with a fluorescent lamp (12 μW/cm^2^) at 25 °C. The solution was analyzed by HPLC as a function of time. The same experiment was carried out for **3**.

### Preparation of light-degraded compounds 4 and 5

The crude mixture of **2** and **3** (34 mg, purity: ~77%, ratio of **2** and **3**: approximately 3:1) was dissolved in MeOH (1.0 L) and poured into a glass bottle and placed in the growth chamber and irradiated with a fluorescent lamp (12 μW/cm^2^) at 25 °C for 8 h. The solution was concentrated *in vacuo*, producing a mixture of **4** and **5** (34 mg). The residue was dissolved in MeOH (30 mL) and purified by preparative HPLC (Develosil ODS-HG-5, 20 mm i.d. × 250 mm) with stepwise gradient elution from 20% to 33% aq. CH_3_CN containing 0.5% TFA. Pure **4** and **5** (4.8 and 2.0 mg, respectively) were obtained as colorless amorphous solids.

### Hexamethylation of light-degraded catechinopyranocyanidin A (4)

To a MeOH solution of **4** (2.5 mg/1 mL), an ether solution of diazomethane (CH_2_N_2_, 1 mL) was added and stirred at rt. Additional CH_2_N_2_ solution was introduced at 0.5, 2, 4, and 8 h, and the solution was stirred at rt continuously for 10 h. The reaction was terminated by the addition of 1% acetic acid (AcOH)-MeOH and the solvent was removed *in vacuo*. The obtained residue was purified by preparative HPLC (Develosil ODS-HG, 4.6 mm i.d. × 250 mm) using linear gradient elution from 10% to 90% aq. CH_3_CN containing 0.5% TFA to give hexamethylated **6** (0.5 mg) from **4** in 17% yield.

### Molecular modeling and ^13^C chemical shift calculations

Conformation searches for the chemical shift calculations were performed with Spartan ’14 and Spartan ’16 (Wavefunction, Irvine, CA)^17^. Models of the 5-keto, 7″-keto, and 4‴-keto forms of **2** were built on Spartan ’16, and a conformational search of each model was performed with MMFF94^29^ by rotating all rotatable C–C and C–O bonds and flipping C2, C3, and C4 to give 399, 409, and 274 candidate conformers, respectively (conformation for the C3′-O and C4′-O, were fixed to take a hydrogen bond and did not rotate during conformation search). These were refined with HF/3–21G and, after removing the overlaps, conformers within 40 kJ/mol from the global minimum conformer were subjected to energy calculation at ωB97X-D/6–31G* level, and then conformers involving more than 15 kJ/mol were discarded. The remained conformers (5-keto: 13, 7″-keto: 30, and 4‴-keto: 22 conformers) were further optimized at the ωB97X-D/6–31 G* and conformers involving more than 3.5 kJ/mol were removed and the remained conformers (5-keto: 13, 7″-keto: 10, and 4‴-keto: 12 conformers) were subjected to energy calculation with fixing the geometries using ωB97X-V/6–311 + G(2df, 2p)[6–311 + G*]//ωB97X-D/6–31 G* to give the Boltzmann distributions. The obtained ^13^C chemical shifts of each elemental conformer were corrected based on its Boltzmann distribution to give theoretical shifts (Table S2). Theoretical ^13^C chemical shifts for 5-keto (remained 11conformers), 7″-keto (11conformers), and 4‴-keto forms (16 conformers) of **3** were calculation in the same manner (Table S3).

### ECD calculations

The ECD curves of **2** and **3** were computed using the Gaussian 16 program^30^, where the 13 and 11 energetically low-lying structures obtained in the chemical-shift calculations were employed for both **2** and **3**, respectively. To examine the effects of the 3,4-dihydroxyphenyl (catechol) group at C2 and hydroxyl group at C3, the ECD curves for model molecules which have only H atoms at C2 and C3 were also examined. To obtain the structures of such models, the catechol and hydroxyl groups at C2 and C3 were replaced by H atoms, and only the positions of newly placed H atoms were optimized with the Cartesian coordinates of the other atoms being fixed. This calculation was performed at the B3LYP/def2-TZVP level. For these obtained structures, the excitation energies and the rotatory strengths for low-lying 15 singlet excited states were computed by using the TD-DFT calculations at the B3LYP/def2-TZVP level. The ECD curves Δ*ε* were computed by using these properties and the Gaussian functions. The further details, including the computed results, are provided in the Supporting Information.

## Supplementary information

Supporting information

## Data Availability

The datasets generated during and/or analysed during the current study are available from the corresponding author on reasonable request.
