# Author Correction: Structure of two purple pigments, catechinopyranocyanidins A and B from the seed-coat of the small red bean, *Vigna angularis*

**DOI:** 10.1038/s41598-019-53406-9

**Published:** 2019-11-08

**Authors:** Kumi Yoshida, Nobukazu Nagai, Yoshiki Ichikawa, Miki Goto, Kohei Kazuma, Kin-ichi Oyama, Kazushi Koga, Masaru Hashimoto, Satoru Iuchi, Yoshiaki Takaya, Tadao Kondo

**Affiliations:** 1 Graduate School of Informatics, Nagoya University, Chikusa, Nagoya, 464-8601 Japan; 2 Graduate School of Information Sciences, Nagoya University, Chikusa, Nagoya, 464-8601 Japan; 3 Graduate School of Human Informatics, Nagoya University, Chikusa, Nagoya, 464-8601 Japan; 4 Research Center for Materials Science, Nagoya University, Chikusa, Nagoya, 464-8602 Japan; 5 Graduate School of Bioagricultural Sciences, Nagoya University, Chikusa, Nagoya, 464-8601 Japan; 6 Faculty of Agriculture and Life Science, Hirosaki University, 3 Bunkyo-cho, Hirosaki, 036-8561 Japan; 7 Faculty of Pharmacy, Meijo University, 150 Yagotoyama, Tenpaku, Nagoya, 468-8503 Japan

Correction to: *Scientific Reports* 10.1038/s41598-018-37641-0, published online 06 February 2019

The original version of this Article contained an error in the title of the paper, where “catechinopyranocyanidins” was incorrectly given as “catechiopyranocyanidins”. This has now been corrected in the PDF and HTML versions of the Article, and in the accompanying Supplementary Information file.

