# Supplementary material for: Structure of two purple pigments, catechinopyranocyanidins A and B from the seed-coat of the small red bean, Vigna angularis
Source: Sci Rep. 2019 Feb 6;9:1484. doi: 10.1038/s41598-018-37641-0 (PMC6365516; doi:10.1038/s41598-018-37641-0)
Supplement: Supplementary file 1 — Supporting information [file 41598_2018_37641_MOESM1_ESM.pdf]

Supporting Information

Structure of two purple pigments, catechinopyranocyanidins A and B from the seed-coat of the small red bean, *Vigna angularis*

Kumi Yoshida[1*], Nobukazu, Nagai[2], Yoshiki Ichikawa[2], Miki Goto[3], Kohei Kazuma[1], Kin-ichi Oyama[4], Kazushi Koga[5], Masaru Hashimoto[6], Satoru Iuchi[1], Yoshiaki Takaya[7] and Tadao Kondo[1]

[1]Graduate School of Informatics, Nagoya University, Chikusa, Nagoya 464-8601, Japan

[2]Graduate School of Information Sciences, Nagoya University, Chikusa, Nagoya 464-8601, Japan

[3]Graduate School of Human Informatics, Nagoya University, Chikusa, Nagoya 464-8601, Japan

[4]Research Center for Materials Science, Nagoya University, Chikusa, Nagoya 464-8602, Japan

[5]Graduate School of Bioagricultural Science, Nagoya University, Chikusa, Nagoya 464-8601, Japan

[6]Faculty of Agriculture and Life Science, Hirosaki University, 3 Bunkyo-cho, Hirosaki, 036-8561, Japan

[7]Faculty of Pharmacy, Meijo University, 150 Yagotoyama, Tenpaku, Nagoya 468-8503, Japan

*To whom correspondence should be addressed. E-mail: yoshidak@i.nagoya-u.ac.jp

Tel & Fax; +819-52-789-5638

Content:

Catechinopyranocyanidin A (**2**) (p.2–18)

Catechinopyranocyanidin B (**3**) (p.19–34)

Photo-degraded catechinopyranocyanidin A (**4**) (p.35–54)

Photo-degraded catechinopyranocyanidin B (**5**) (p.55–64)

Hexamethylphoto-degraded catechinopyranocyanidin A (**6**) (p.65–71)

Calculation of chemical shift of ^{13}C NMR of **2** and **3** (p.72–73)

Absolute configuration of **2** and **3** (p.74–79)

Purple color development of **2** and **3** (p.80)

Comparison of the structure with vignacyanidin (p.81)

Proposed mechanism of photo-degradation of Catechinopyranocyanidin A (**2**) (p.82)

Supplementary References

S1. Lee, C., Yang, W. & Parr, R. G. Development of the Colle-Salvetti correlation-energy formula into a functional of the electron density. *Phys. Rev. B* **37**, 785-789 (1988). Miehlich, B., Savin, A., Stoll, H., & Preuss, H. Results obtained with the correlation energy density functionals of Becke and Lee, Yang and Parr. *Chem. Phys. Lett.* **157**, 200-206 (1989). Becke, A. D. Density-functional thermochemistry. III. The role of exact exchange. *J. Chem. Phys.* **98**, 5648-5652 (1993).

S2. Weigend, F. & Ahlrichs, R. Balanced basis sets of split valence, triple zeta valence and quadruple zeta valence quality for H to Rn: Design and assessment of accuracy. *Phys. Chem. Chem. Phys.* **7**, 3297-3305 (2005).

S3. Stephens, P. J. & Harada, N. ECD Cotton effect approximated by the Gaussian curve and other methods. *Chirality* **22**, 229-233 (2010).

Catechinopyranocyanidin A (2)

(10*R*,11*S*)-2,10-bis(3,4-dihydroxyphenyl)-1,5,11-trihydroxy-11,12-dihydrodipyrano[3,2-*b*:2',3',4'-*kl*]xanthen-13(10*H*)-one

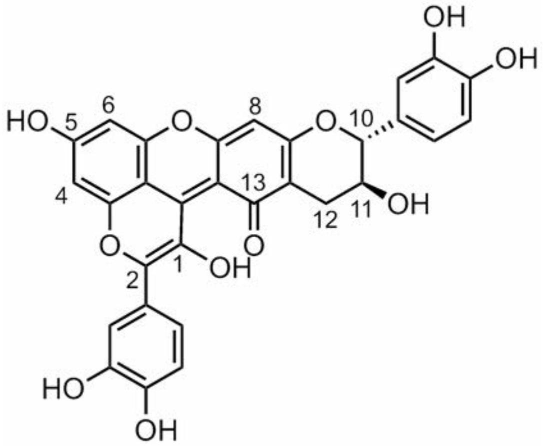

(The numbering of carbon atoms in the structure is different from that in the main text, but only for the IUPAC nomenclature)

ECD λ, (Δ*ε*): 570 nm (+2.19), 415 nm (–3.37), 349 nm (+0.98) (1.93x10^{-5}M, MeOH), UV λ_{max} (*ε*): 574 nm (28,400), 418 nm (12,300), 280 nm (15,800) (1.93x10^{-5}M, MeOH), IR (KBr) ν_{max} cm^{-1}: 3442, 3175, 1681, 1598, 1397, 1187, 841, 807, 724, HR-MS (ESI) Calcd for $C_{30}H_{21}O_{11}$ $[M+H]^+$: 557.1078, Found: 557.1071, Calcd for $C_{30}H_{20}O_{11}Na$ $[M+Na]^+$: 579.0898, Found: 579.0891.

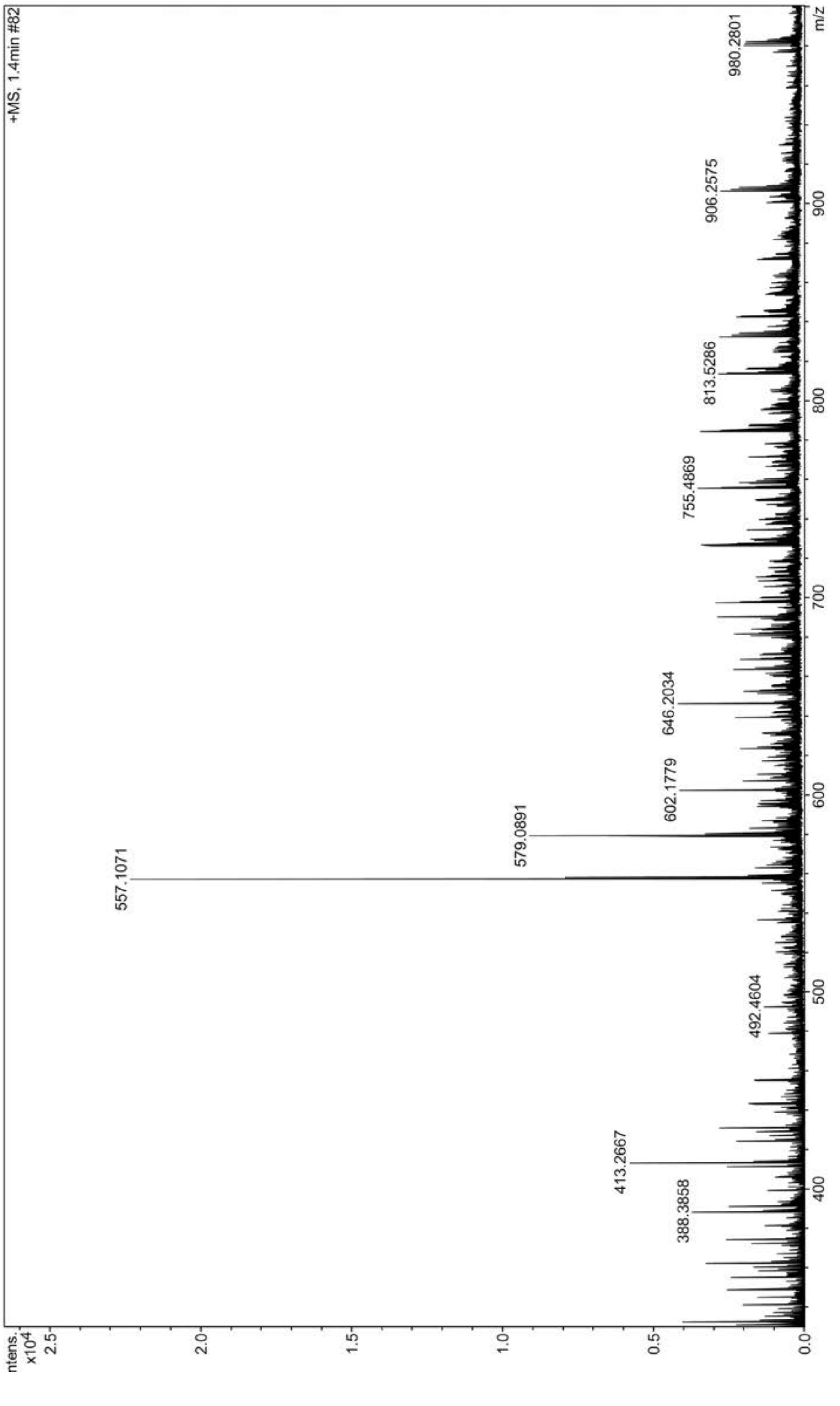

Figure S1-1: HR-ESI-TOF MS of **2**.

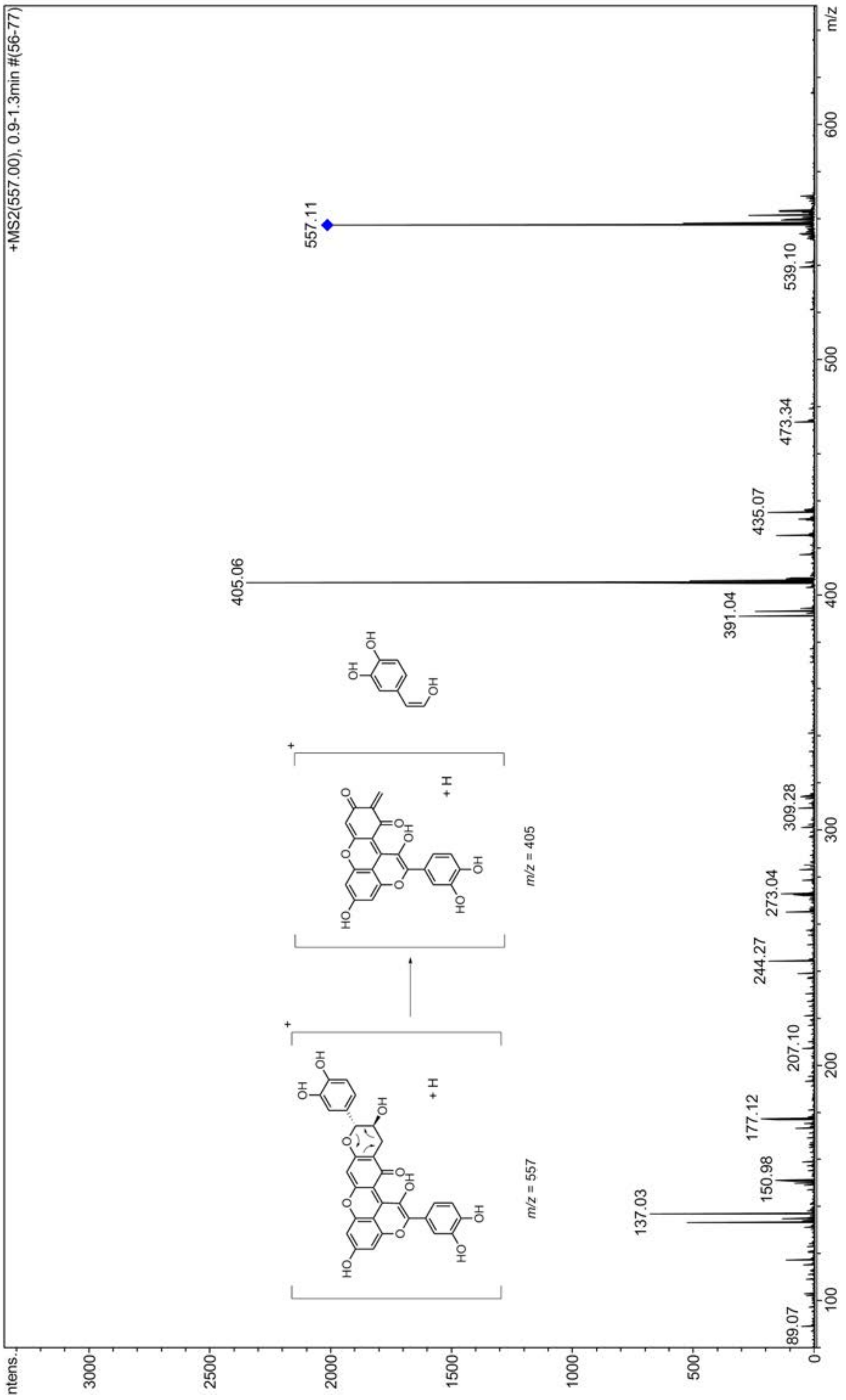

Figure S1-2: MS/MS of **2**.

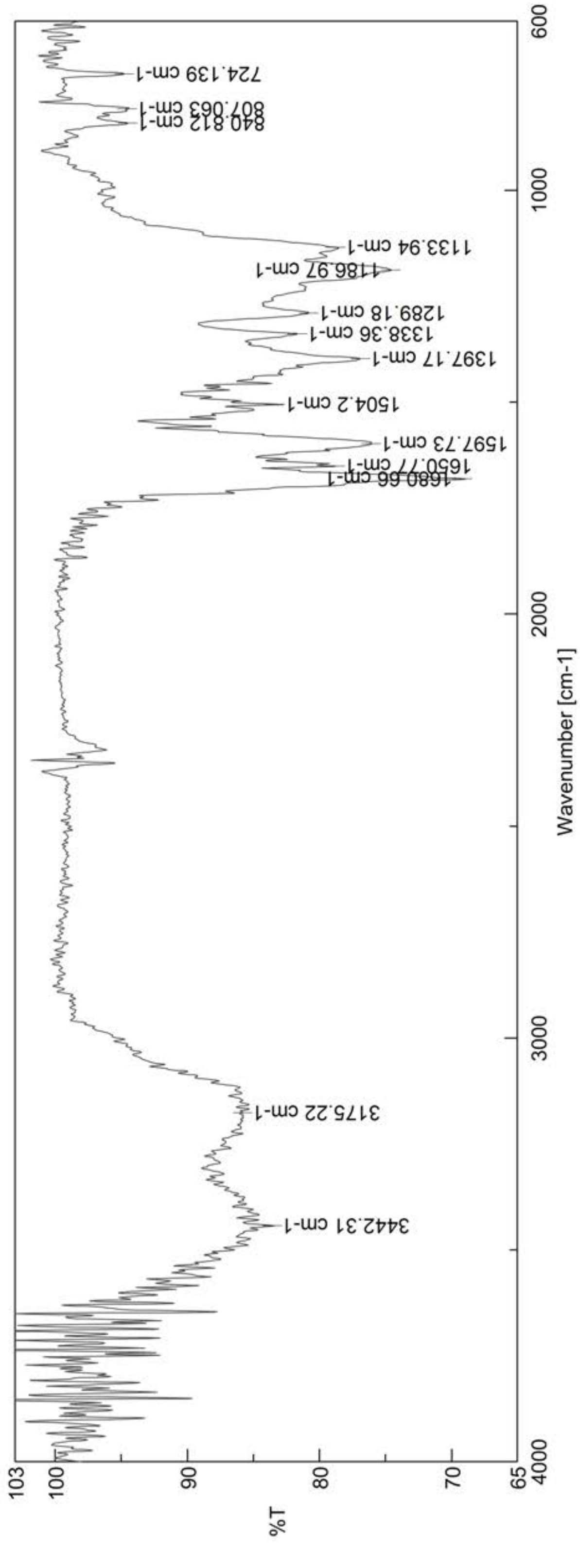

Figure S2: IR of **2**.

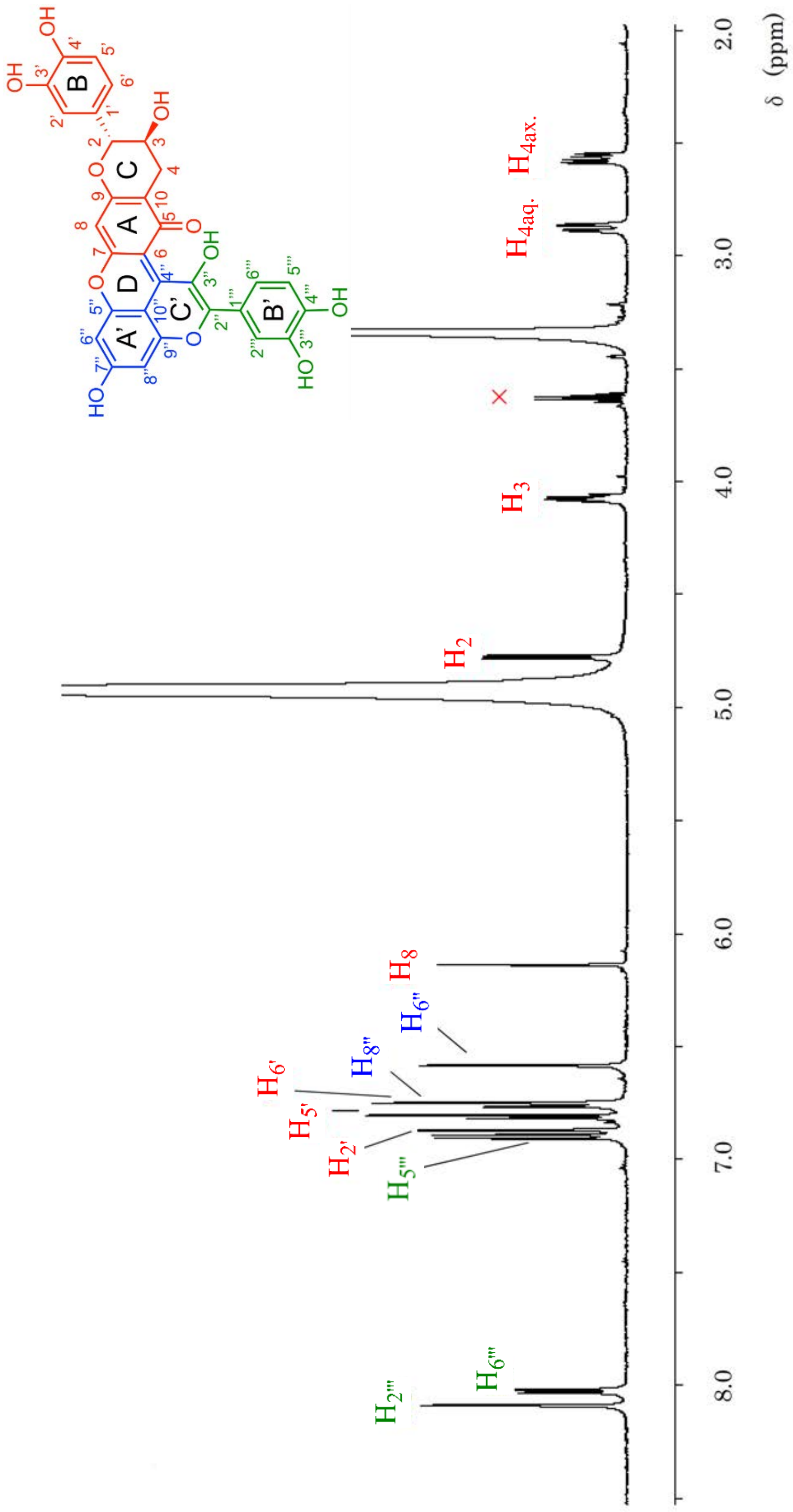

Figure S3: ^{1}H-NMR of **2** (^{1}H: 600 MHz, CD_3OD, rt).

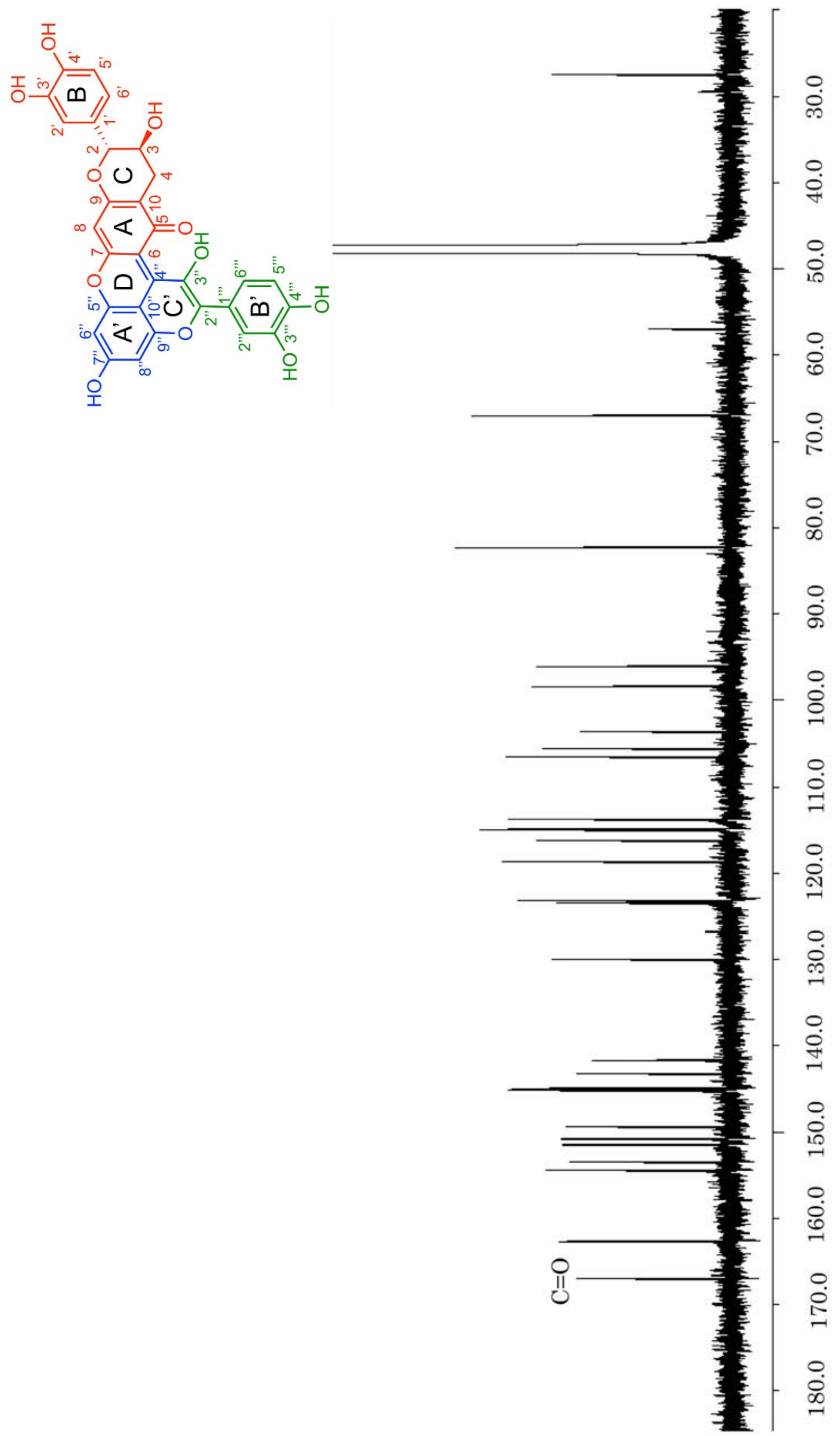

Figure S4: ^{13}C-NMR of **2** (^{13}C: 150 MHz, CD_3OD, rt).

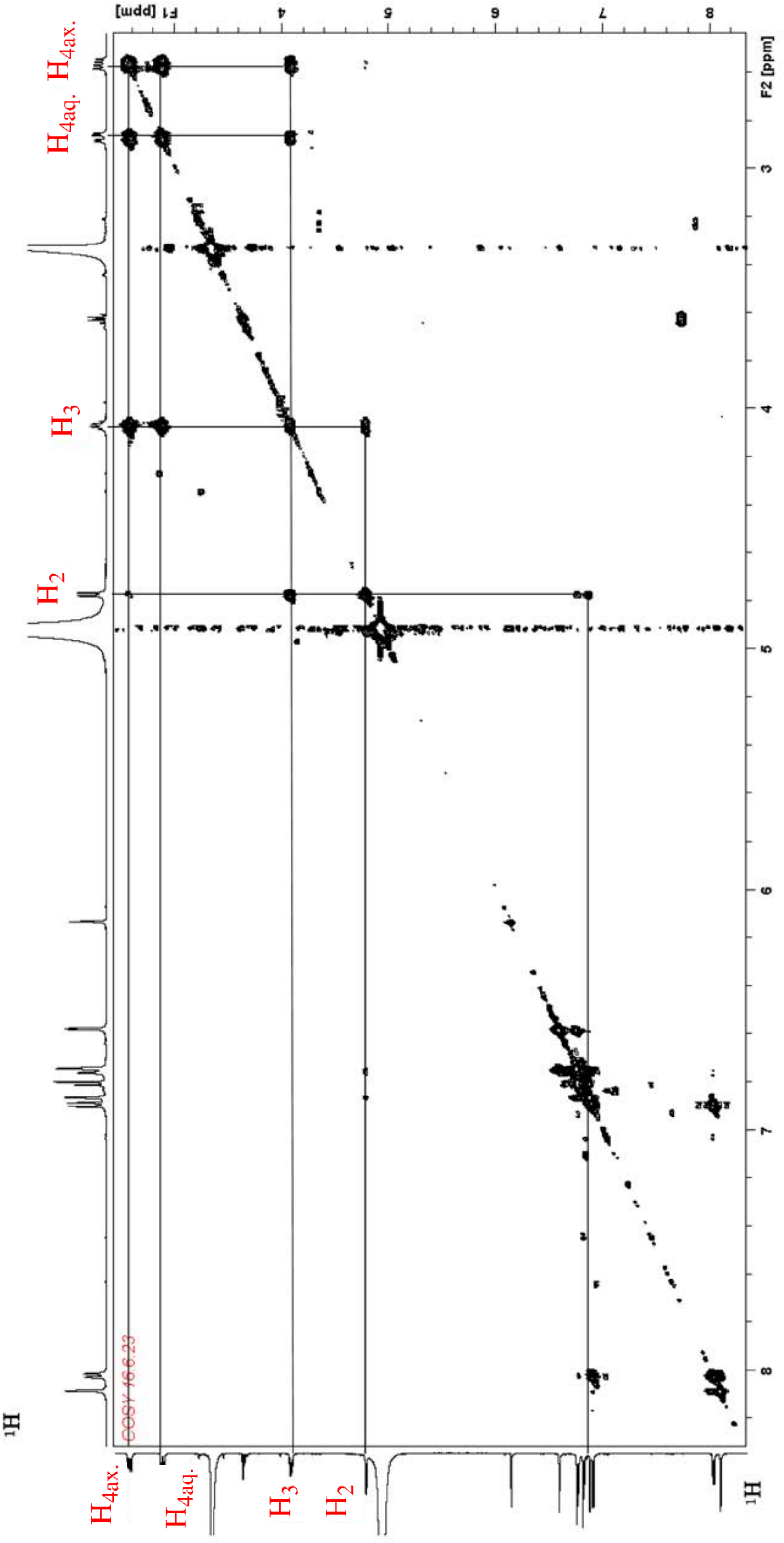

Figure S5-1: COSY of **2** (^{1}H: 600 MHz, CD_3OD, rt).

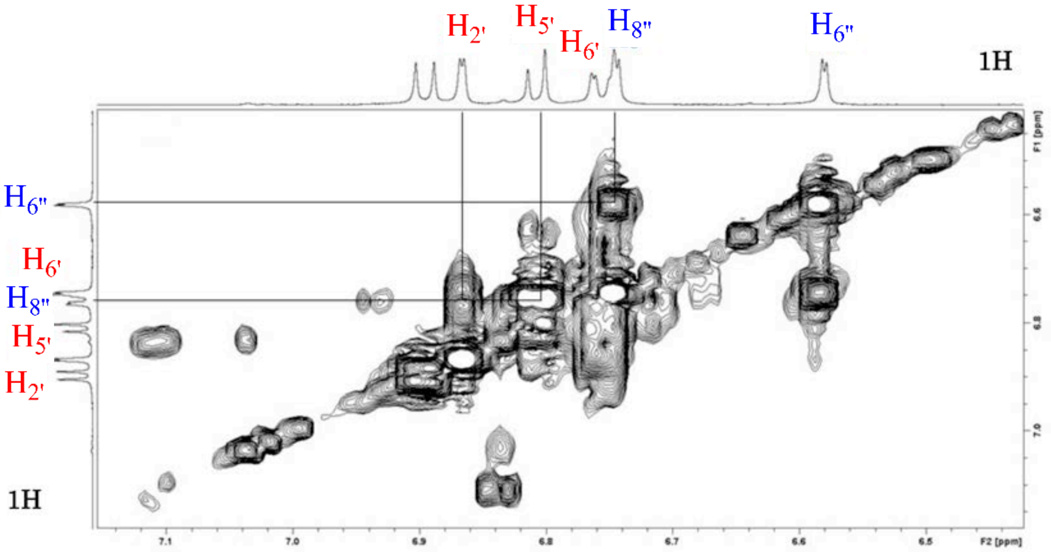

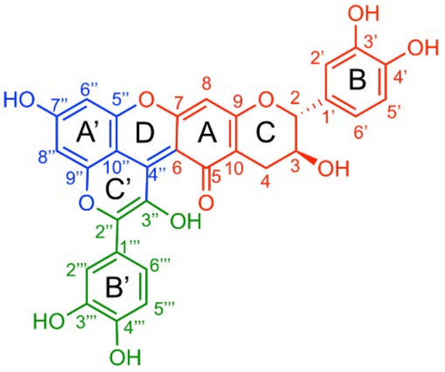

Figure S5-2: Expanded spectrum of COSY of **2** in lower field and observed correlations (^{1}H: 600 MHz, CD_3OD, rt).

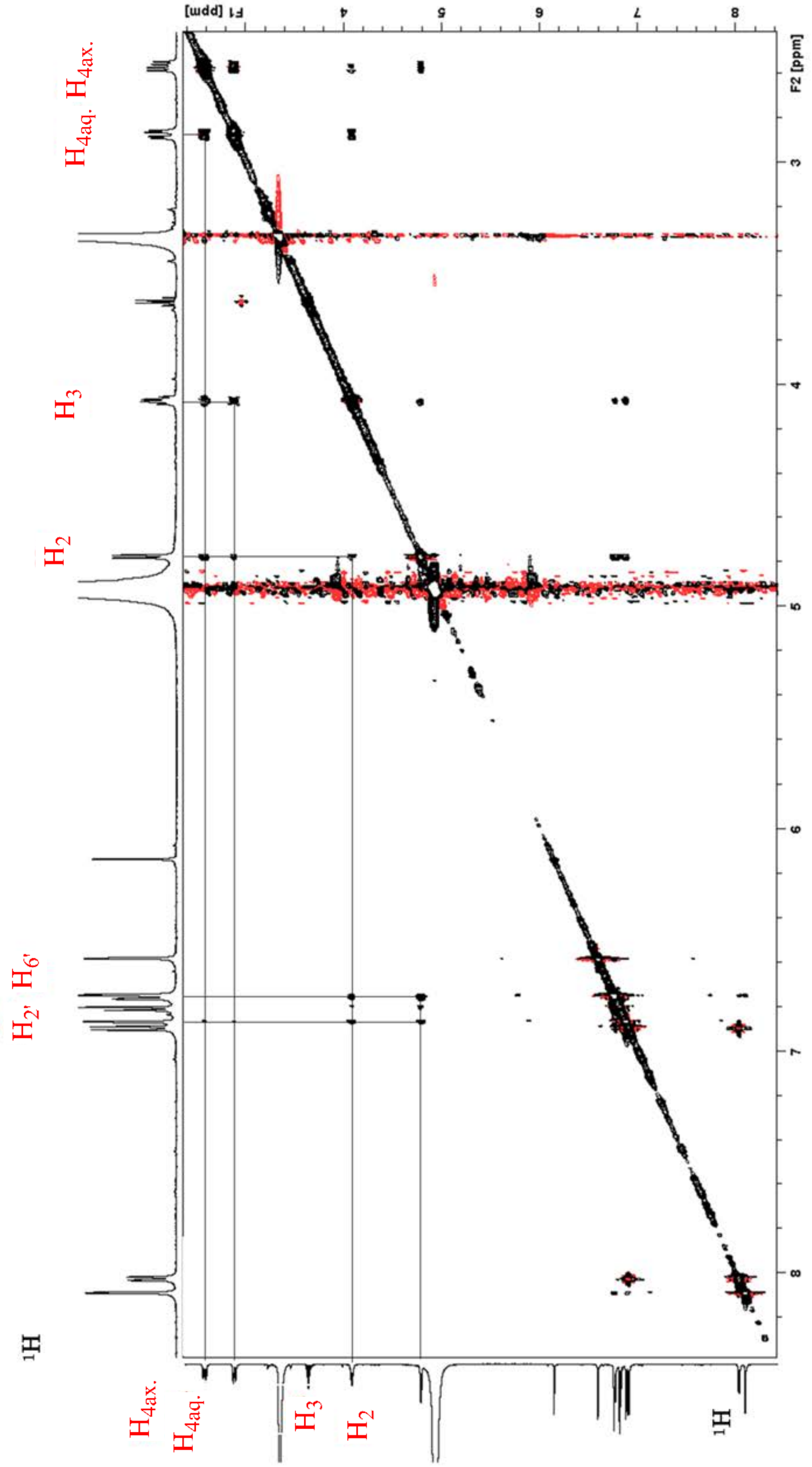

Figure S6-1: NOESY of **2** (^{1}H: 600 MHz, CD_3OD, rt).

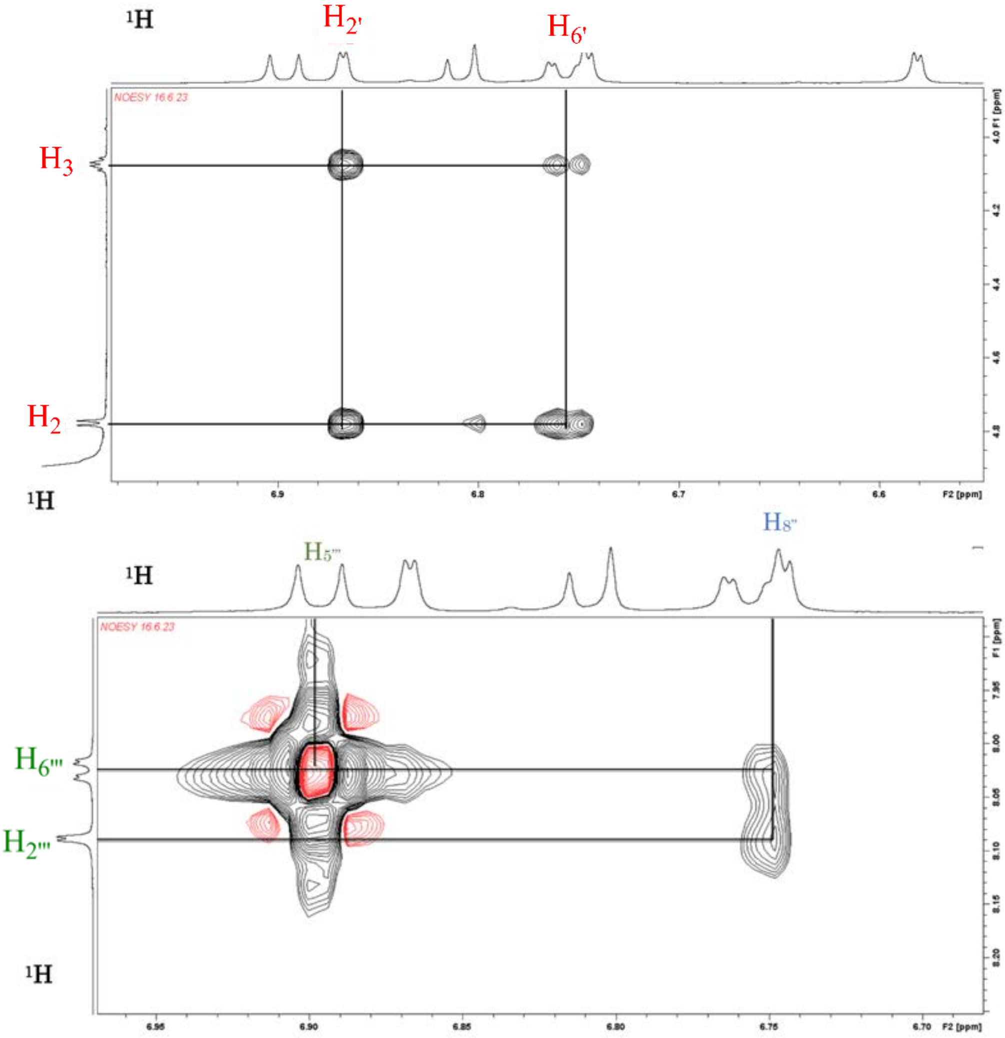

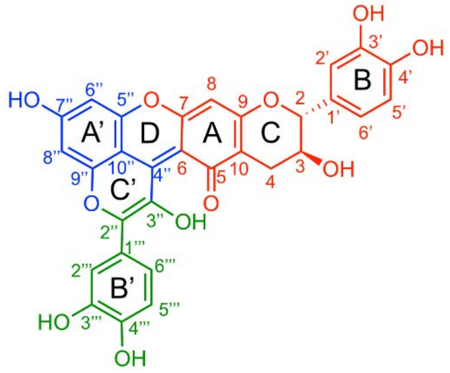

Figure S6-2: Expanded spectra of NOESY of **2** and the observed correlations (^{1}H: 600 MHz, CD_3OD, rt).

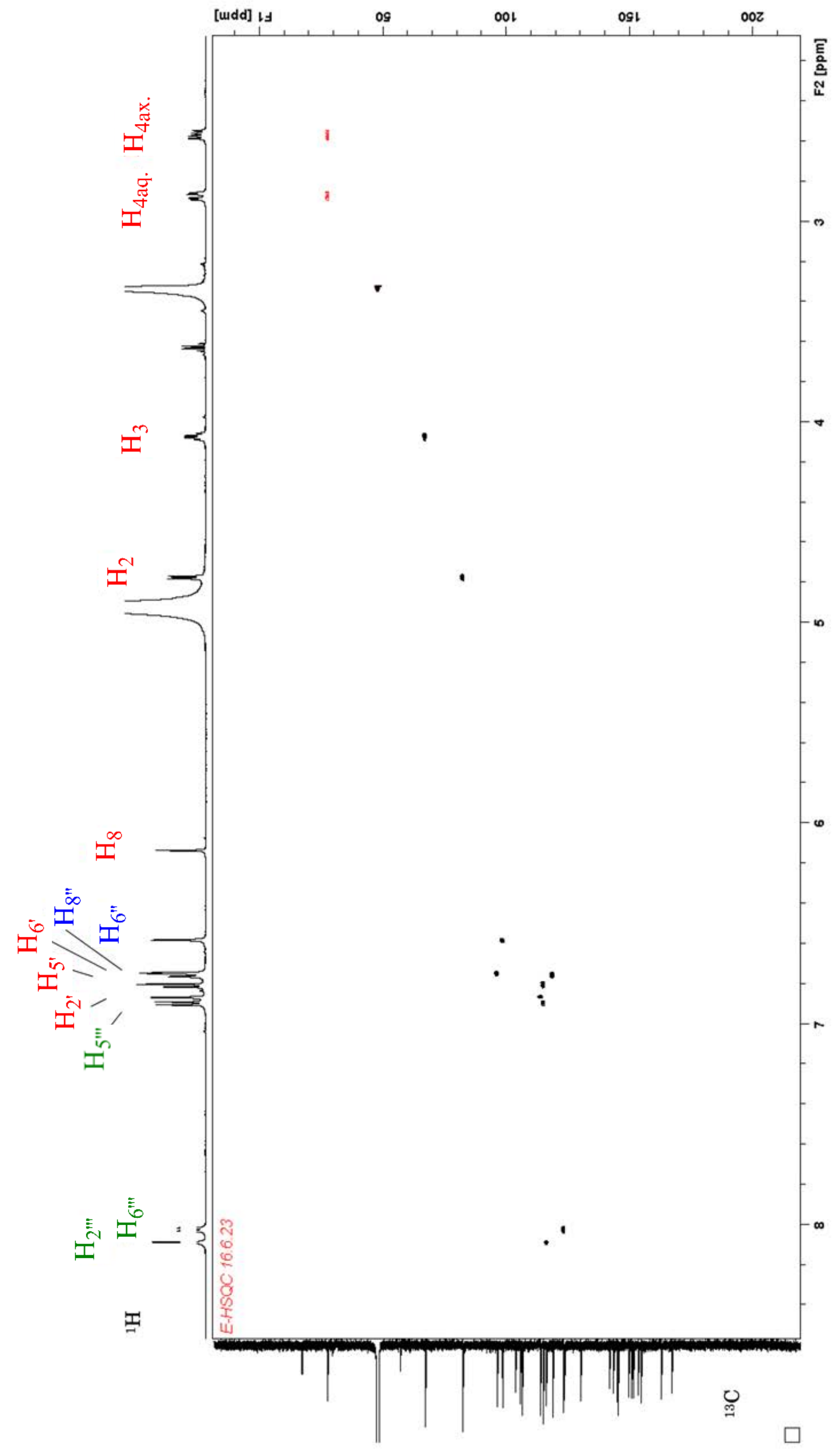

Figure S7-1: HSQC of **2**. (^{1}H: 600 MHz, ^{13}C: 150 MHz, CD_3OD, rt).

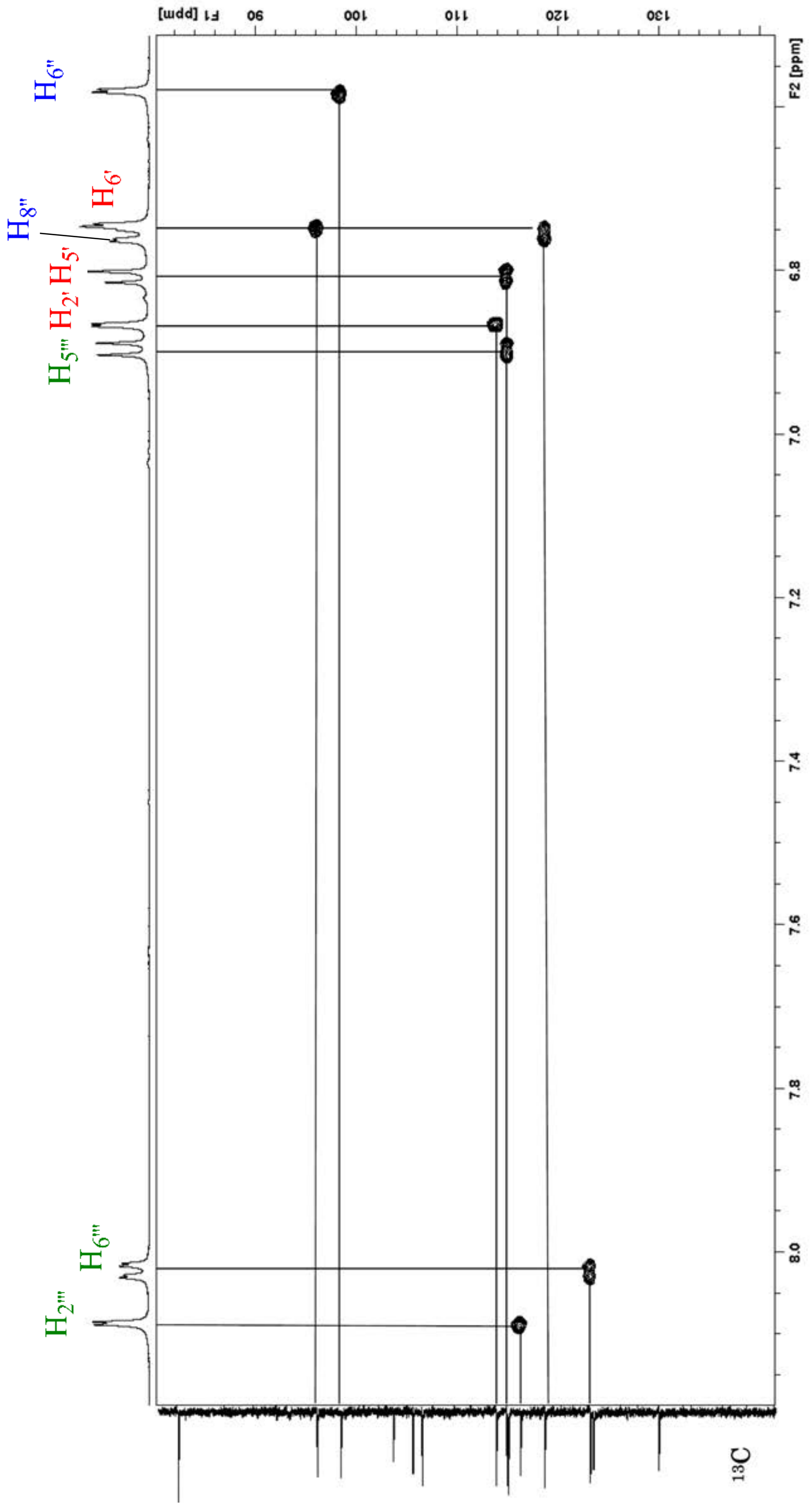

Figure S7-2: Expanded spectrum of HSQC of **2** with lower field in ^{1}H NMR (^{1}H: 600 MHz, ^{13}C: 150 MHz, CD_3OD, rt).

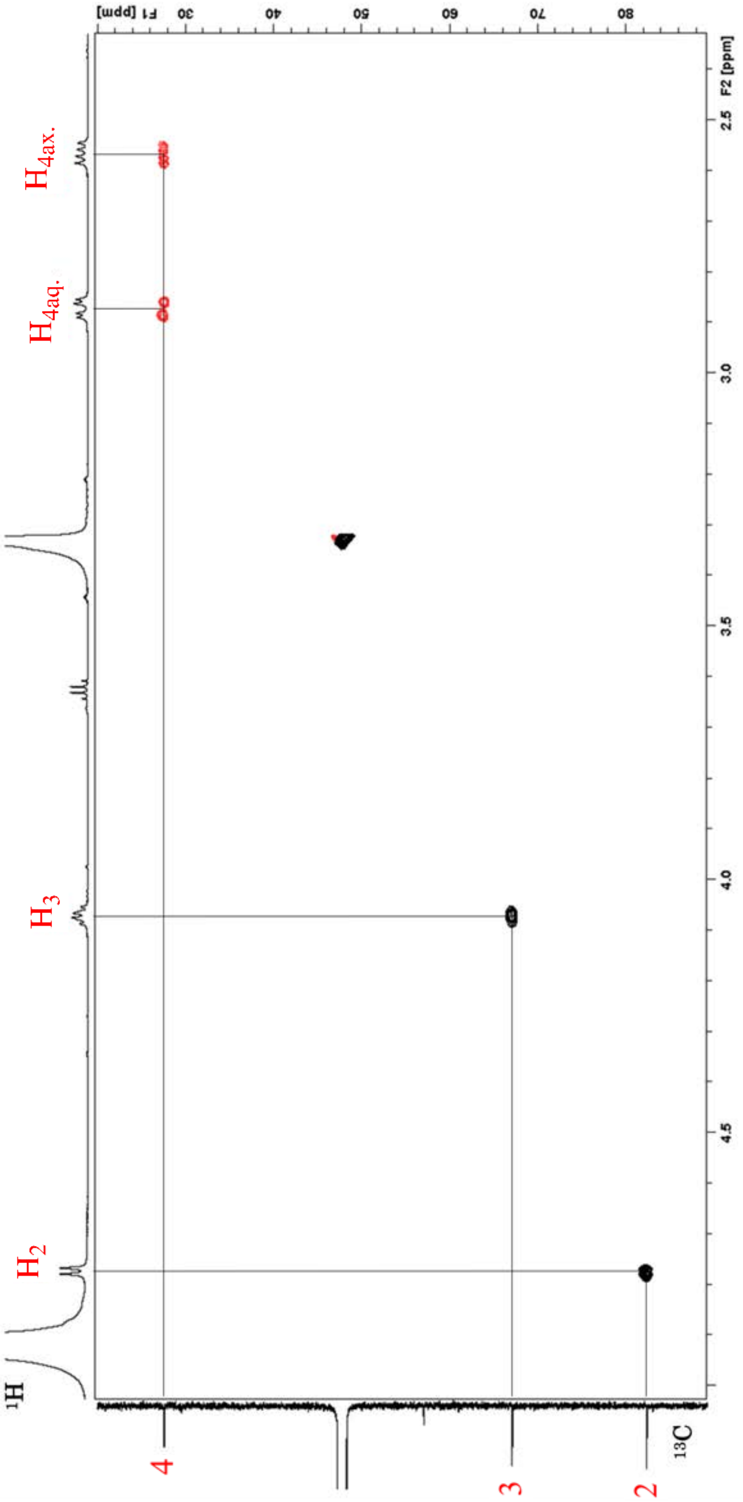

Figure S7-3: Expanded spectrum of HSQC of **2** with higher field in ^{1}H NMR (^{1}H: 600 MHz, ^{13}C: 150 MHz, CD_3OD, rt).

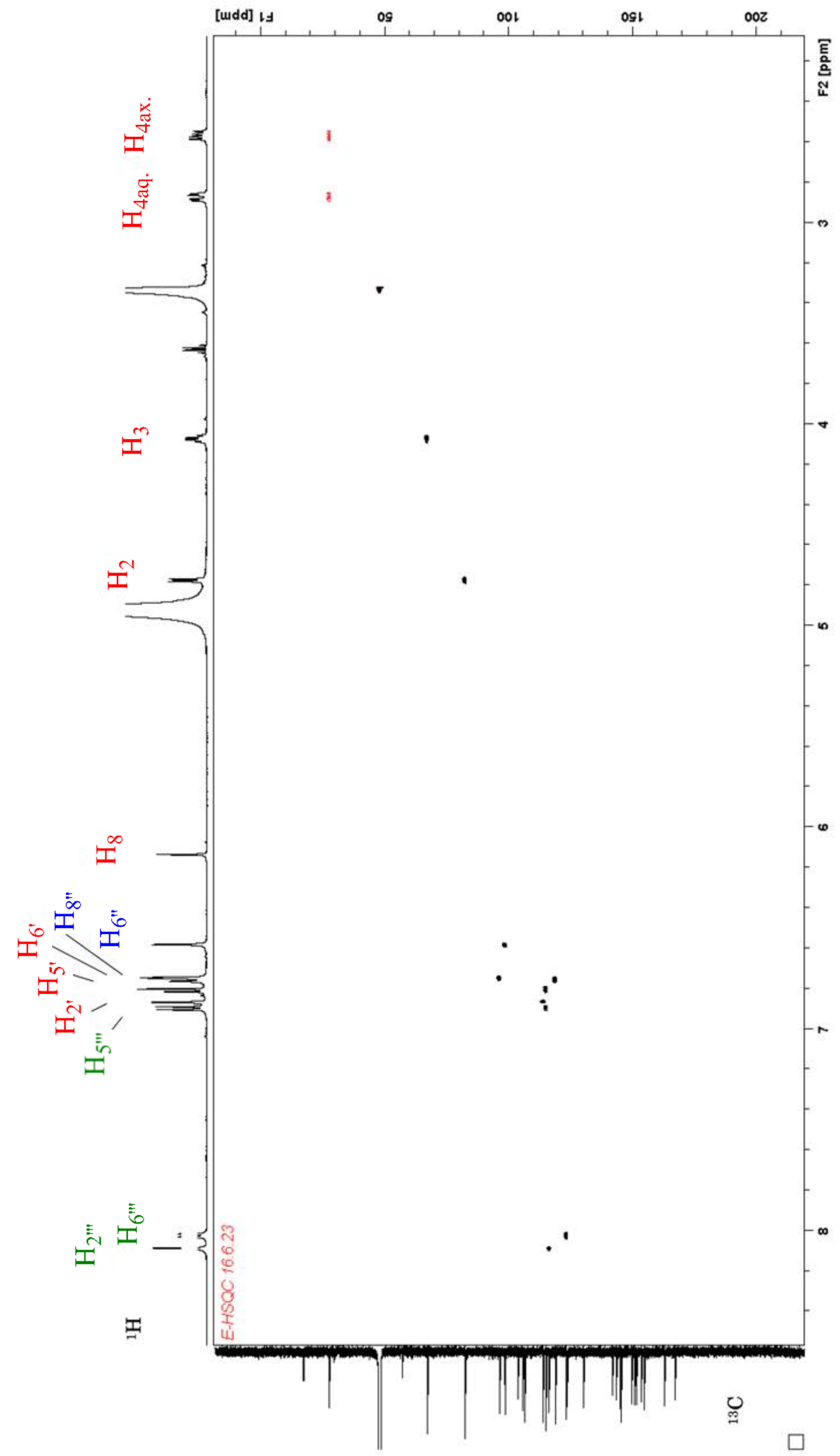

Figure S8-1: HMBC of **2**

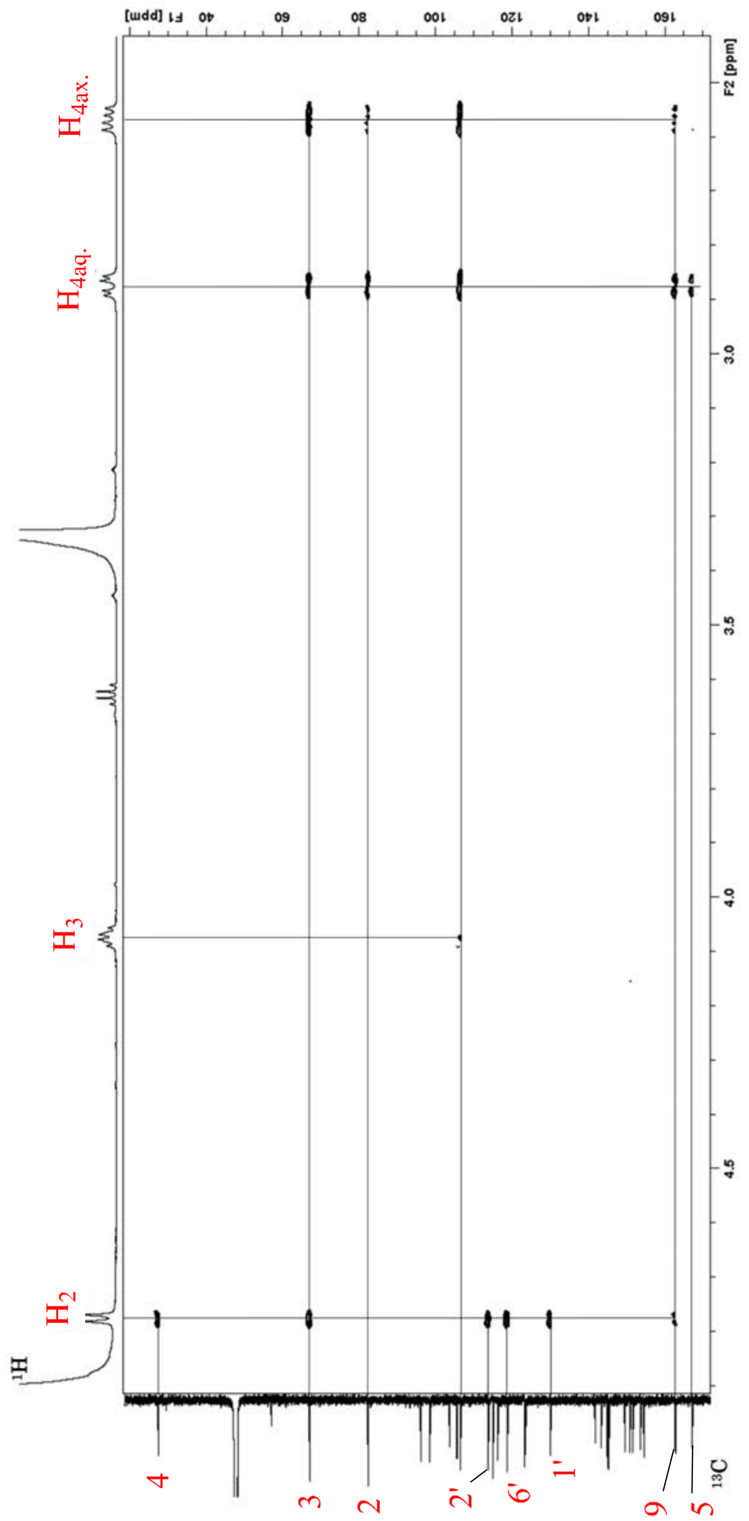

Figure S8-2: Expanded spectrum of HMBC of **2** with higher field in ^{1}H NMR (^{1}H: 600 MHz, ^{13}C: 150 MHz, CD_3OD, rt).

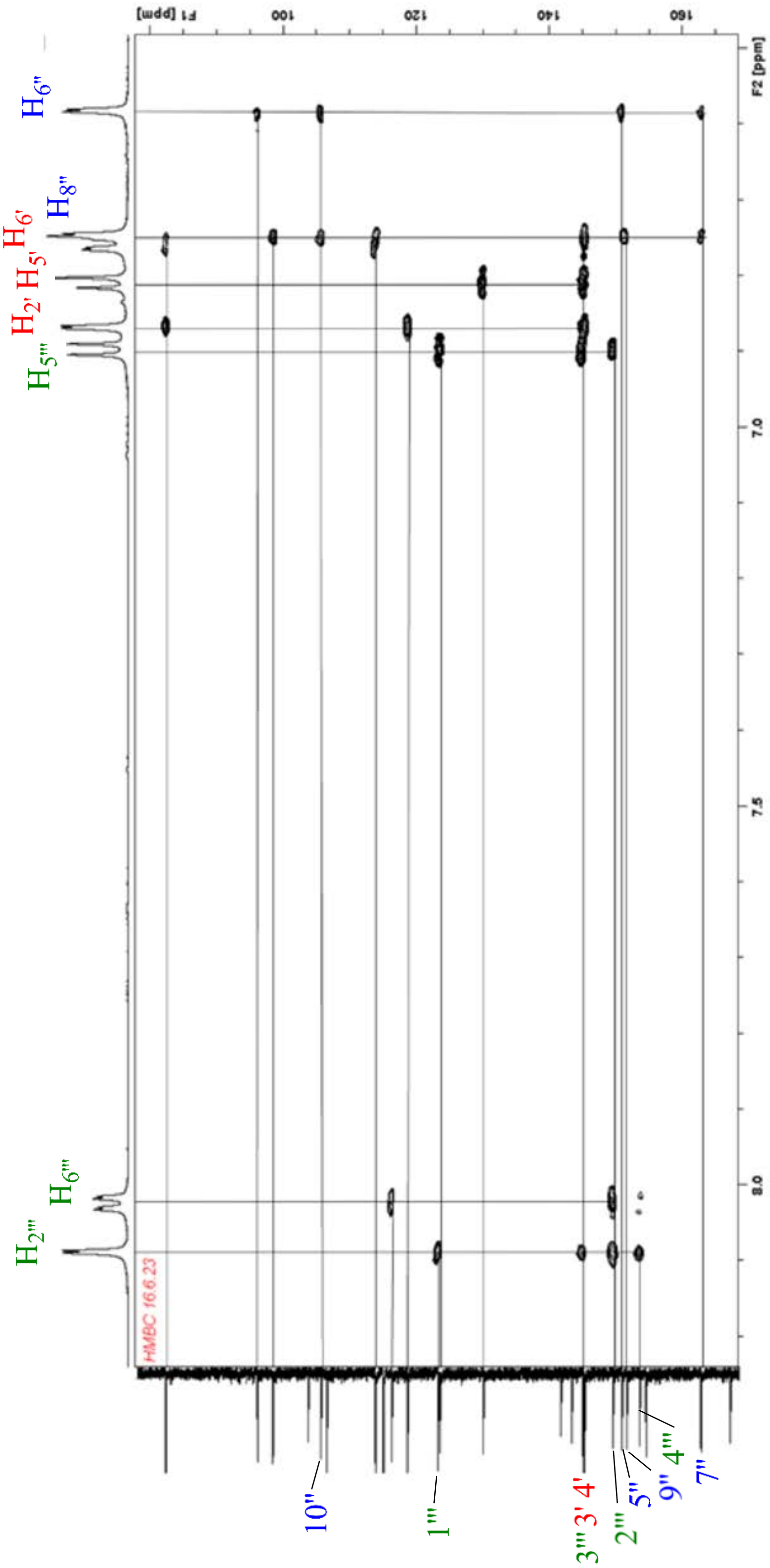

Figure S8-3: Expanded spectrum of HMBC of **2** with lower field in ^{1}H NMR (^{1}H: 600 MHz, ^{13}C: 150 MHz, CD_3OD, rt).

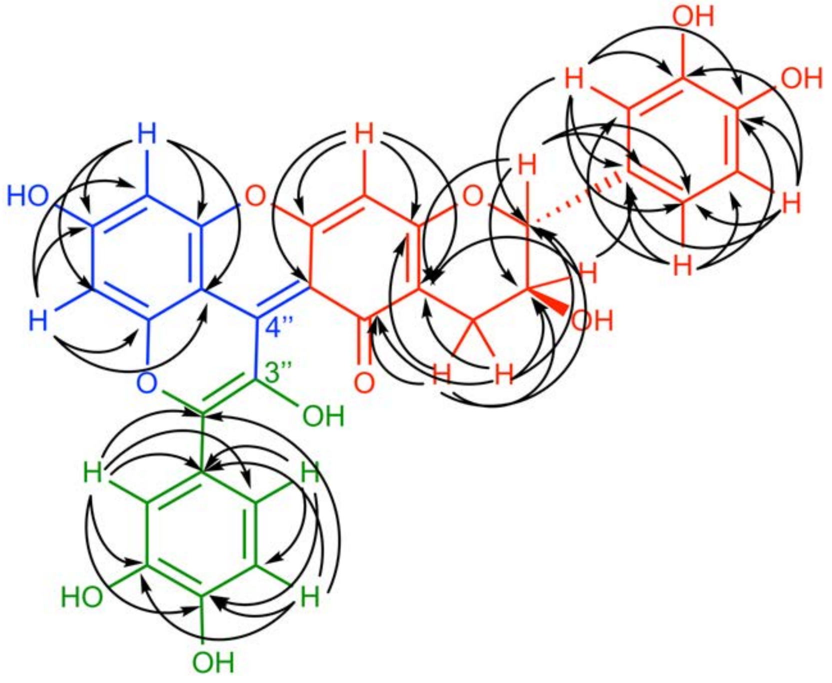

Figure S8-4: Observed HMBC correlations of **2**.

Catechinopyranocyanidin B (3)

(10*S*,11*S*)-2,10-bis(3,4-dihydroxyphenyl)-1,5,11-trihydroxy-11,12-dihydrodipyrano[3,2-*b*:2',3',4'-*kl*]xanthen-13(10*H*)-one

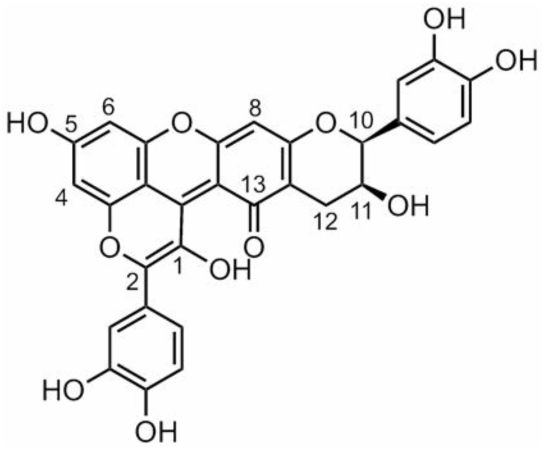

(The numbering of carbon atoms in the structure is different from that in the main text, but only for the IUPAC nomenclature)

ECD λ, (Δ*ε*): 570 nm (–1.75), 415 nm (+1.25), 349 nm (–0.84) (3.07×10^{-5}M, MeOH), UV λ_{max} (*ε*): 574 nm (26,300), 418 nm (11,400), 280 nm (14,900) (3.07×10^{-5}M, MeOH), IR (KBr) ν_{max} cm^{-1}: 3155, 1682, 1400, 1207, 841, 804, 724, HR-MS (ESI) Calcd for $C_{30}H_{21}O_{11}$ $[M+H]^+$: 557.1078, Found: 557.1073, Calcd for $C_{30}H_{20}O_{11}Na$ $[M+Na]^+$: 579.0898, Found: 579.0891

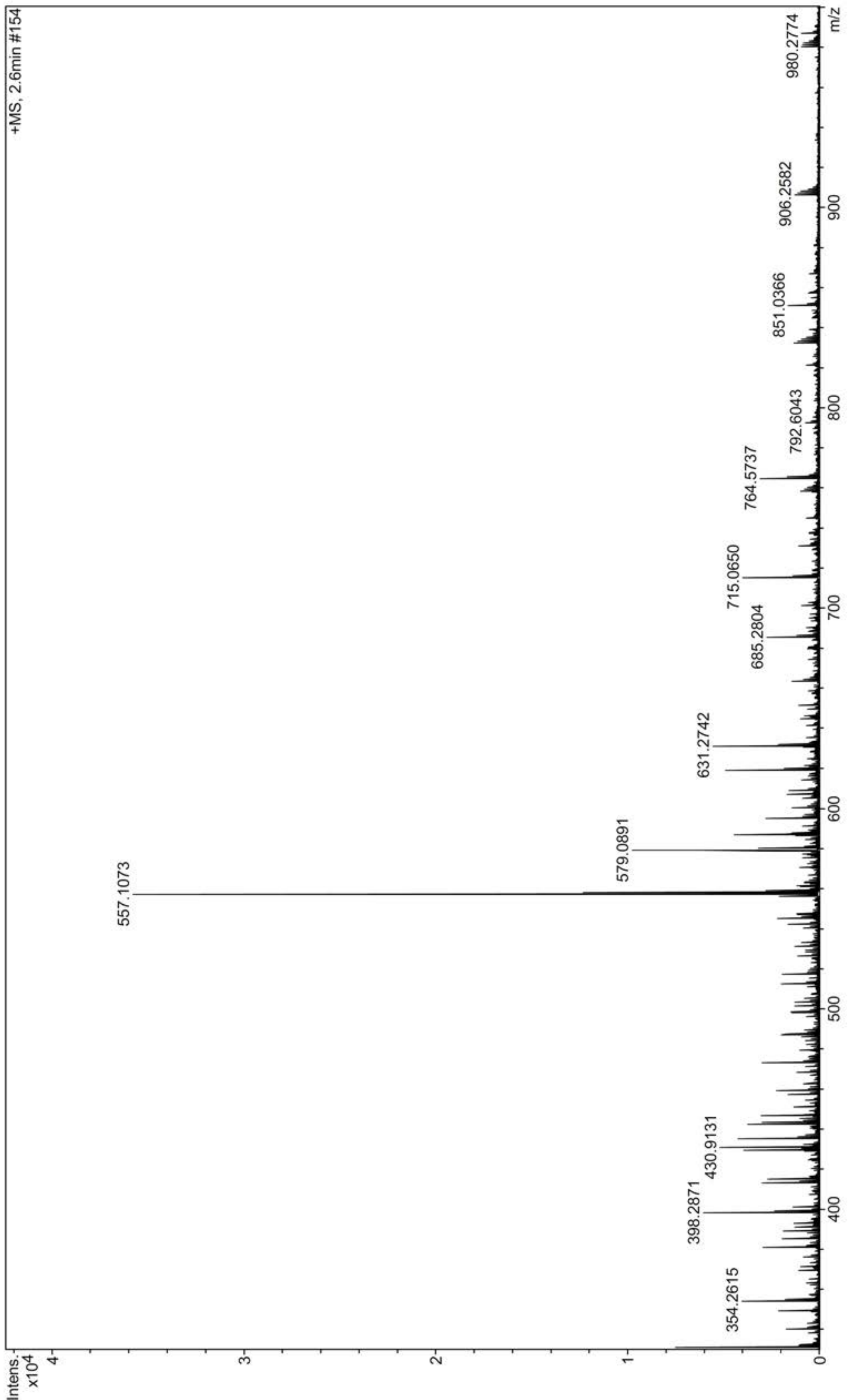

Figure S9-1: HR-ESI-TOF MS of **3**.

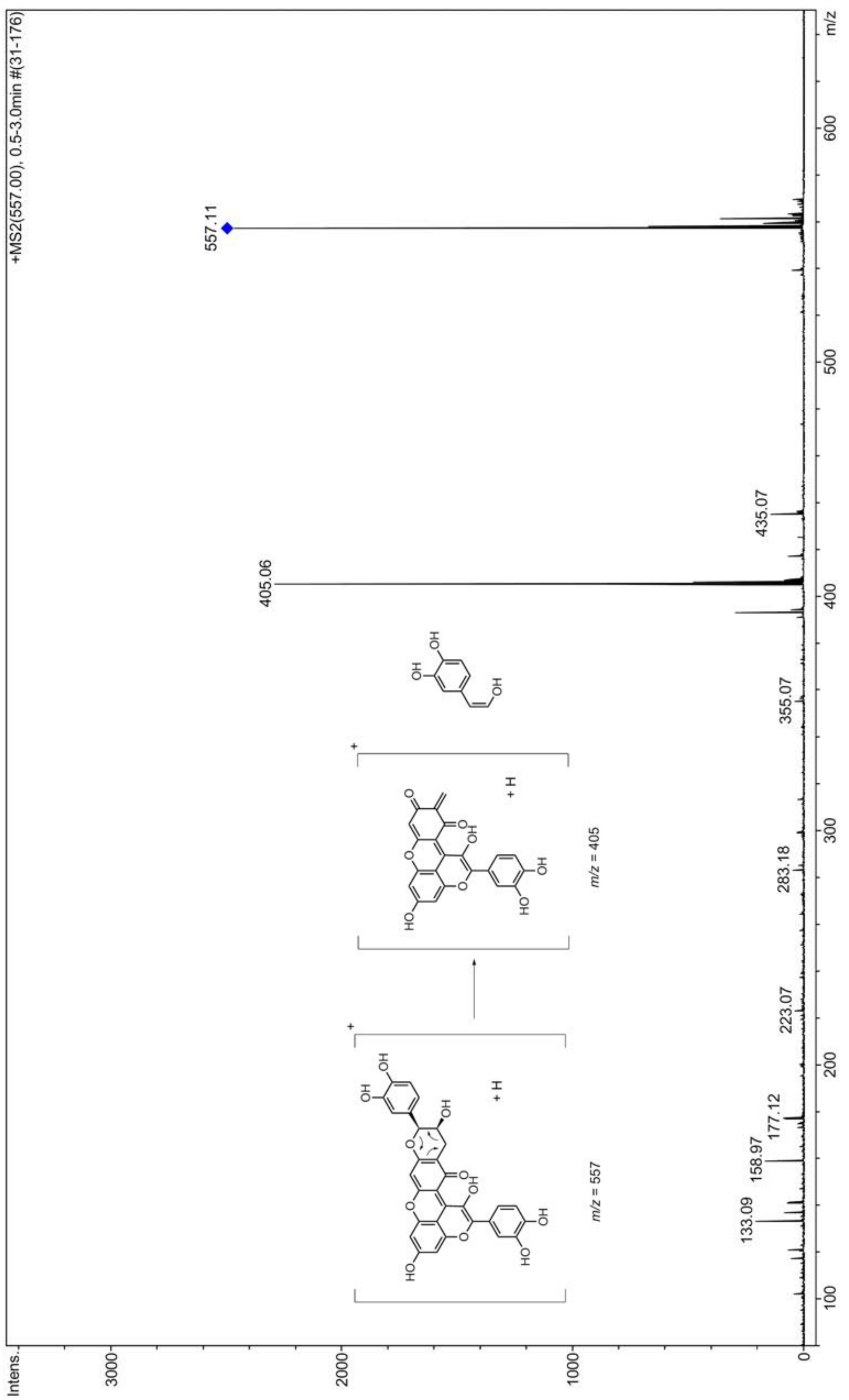

Figure S9-2: MS/MS of **3**.

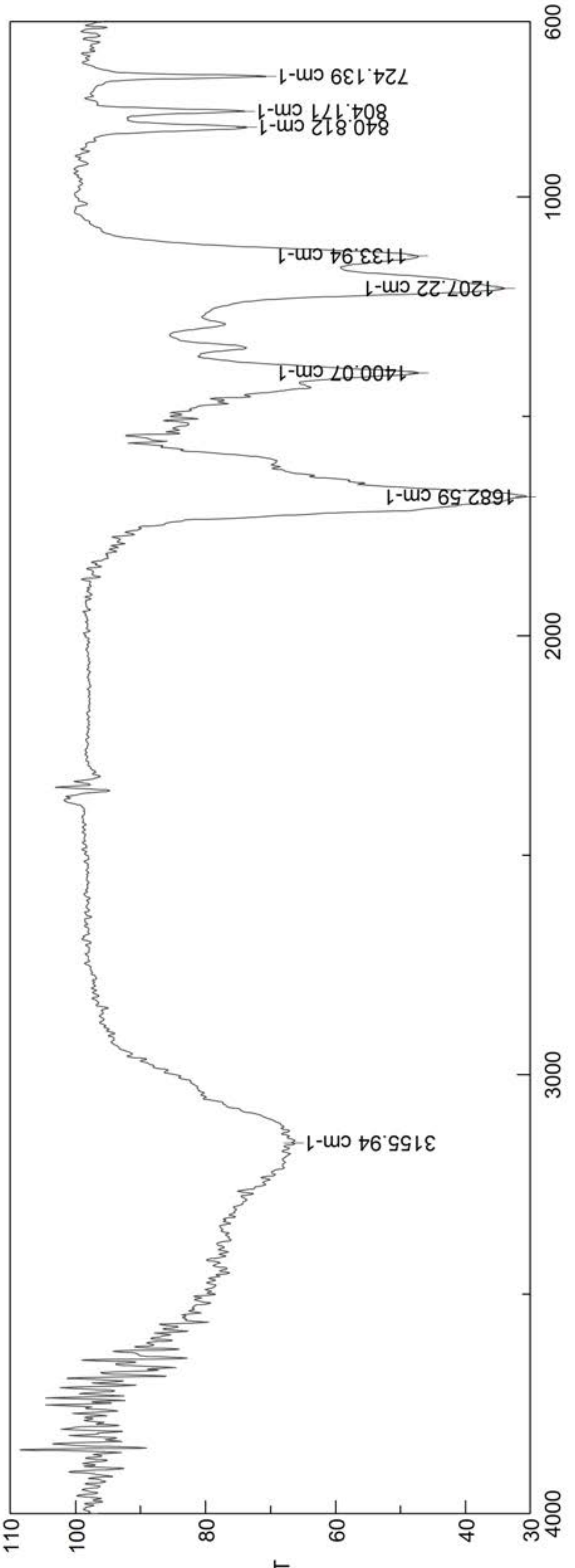

Figure S10: IR of **3**.

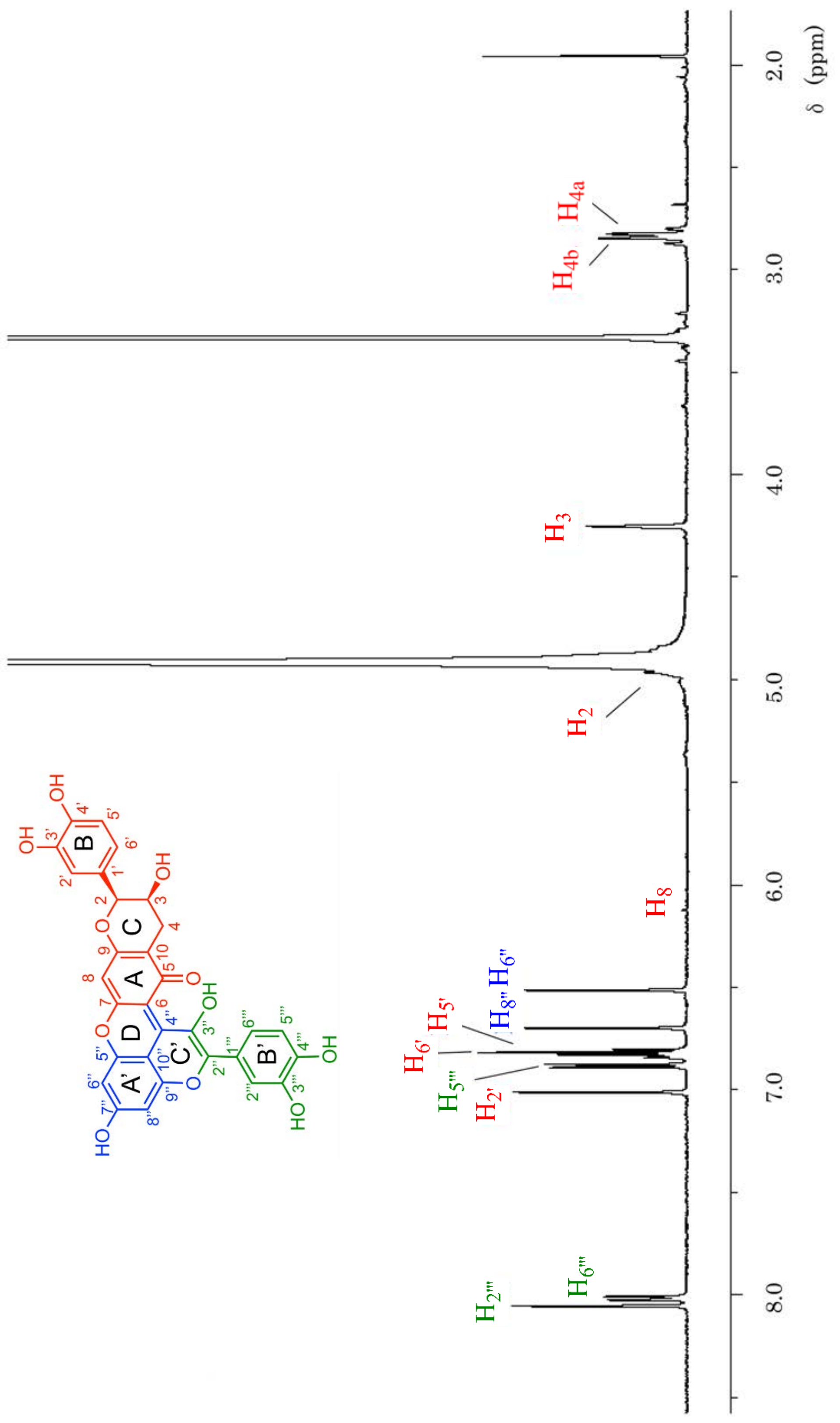

Figure S11: ^{1}H-NMR of **3** (^{1}H: 600 MHz, CD_3OD, rt).

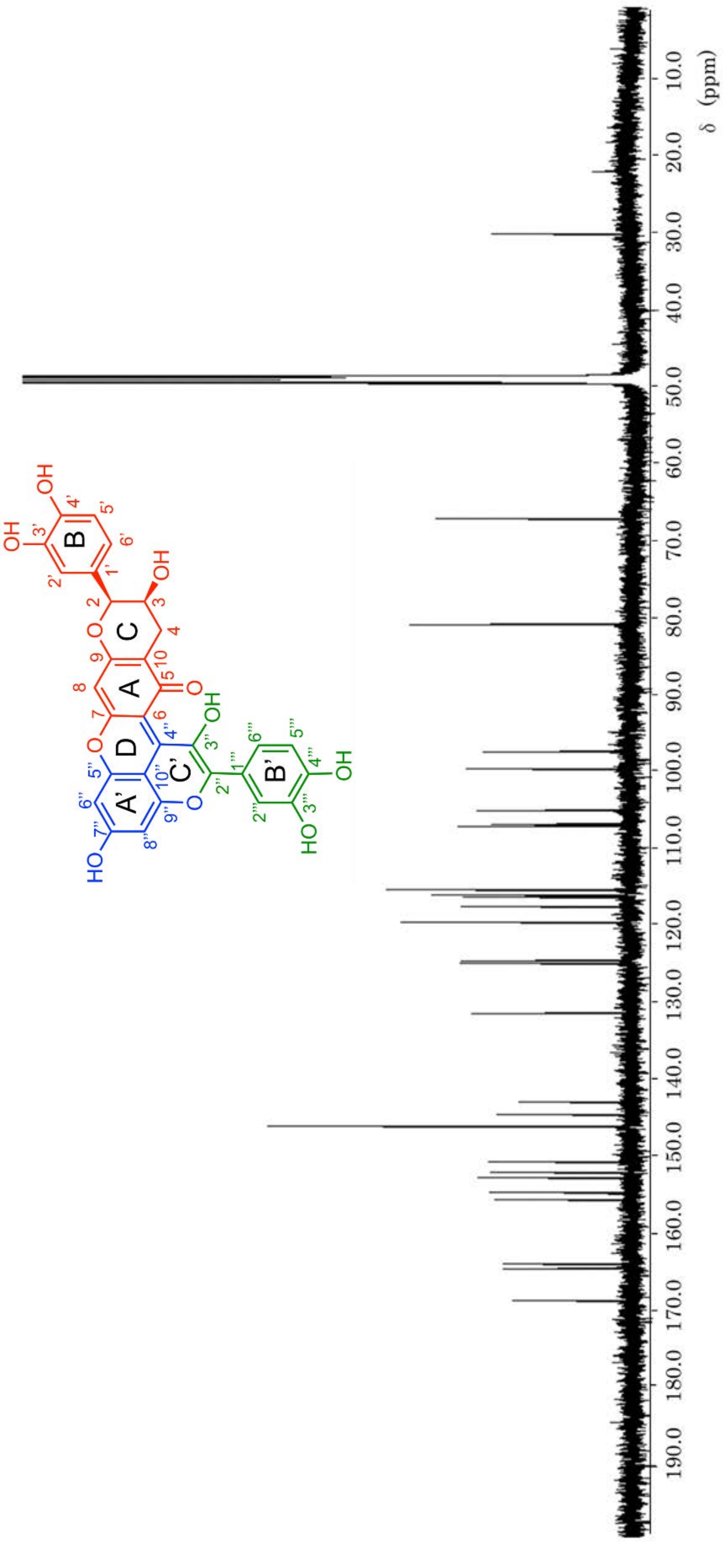

Figure S12: ^{13}C-NMR of **3** (^{13}C: 150 MHz, CD_3OD, rt).

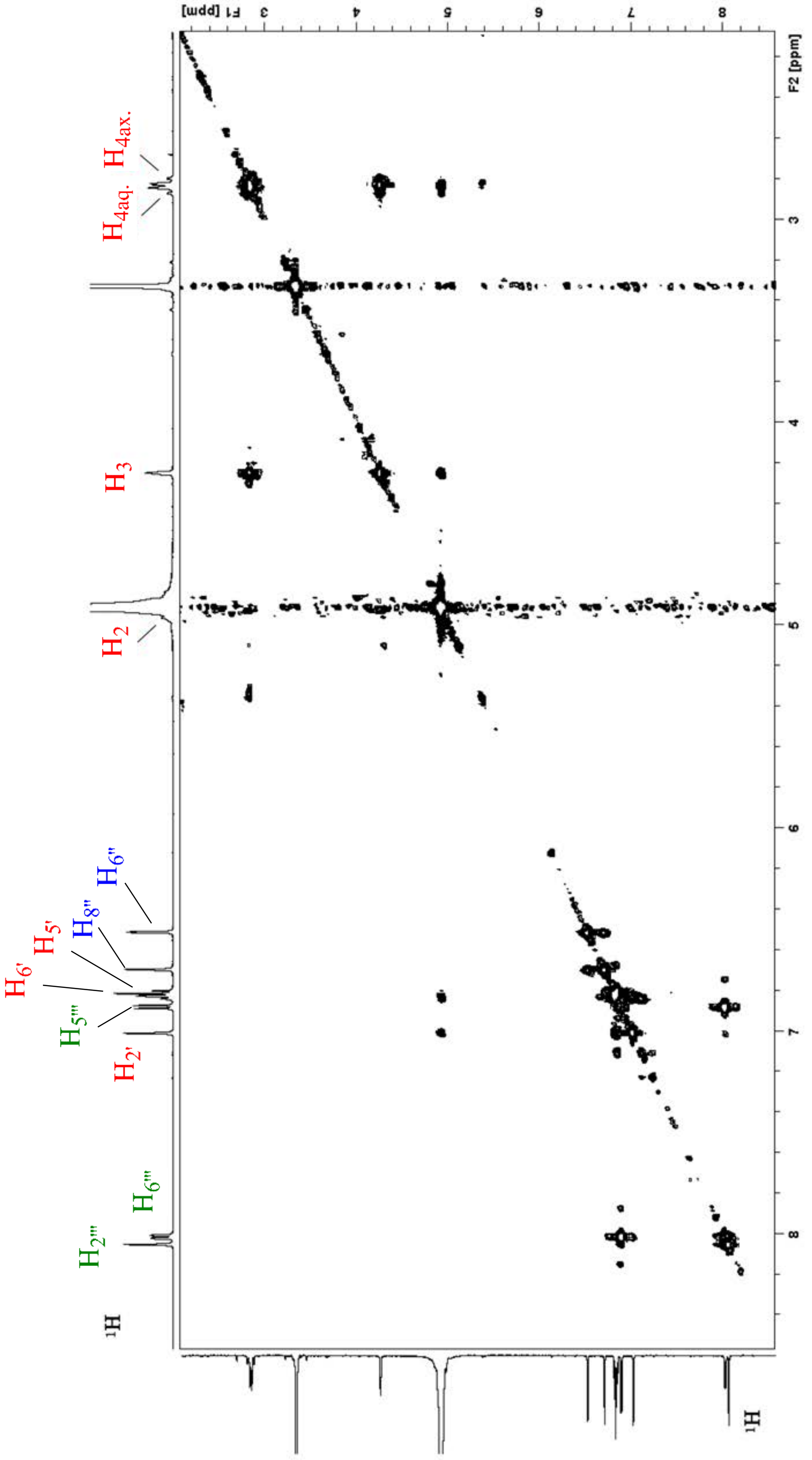

Figure S13-1: COSY of **3** (^{1}H: 600 MHz, CD_3OD, rt).

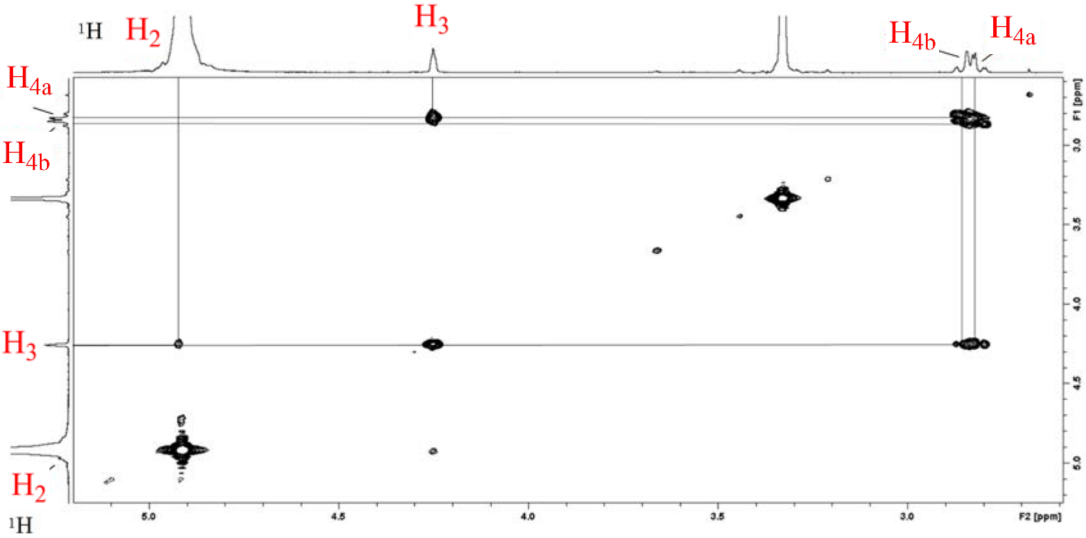

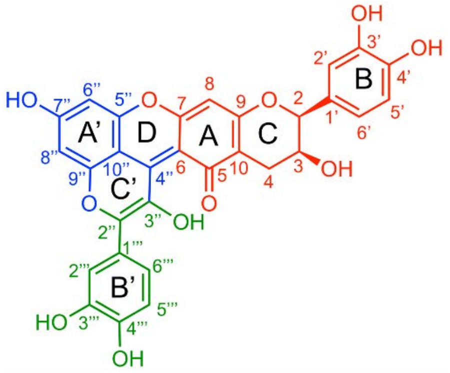

Figure S13-2: Expanded spectrum of COSY of **3** and observed correlations (^{1}H: 600 MHz, CD_3OD, rt).

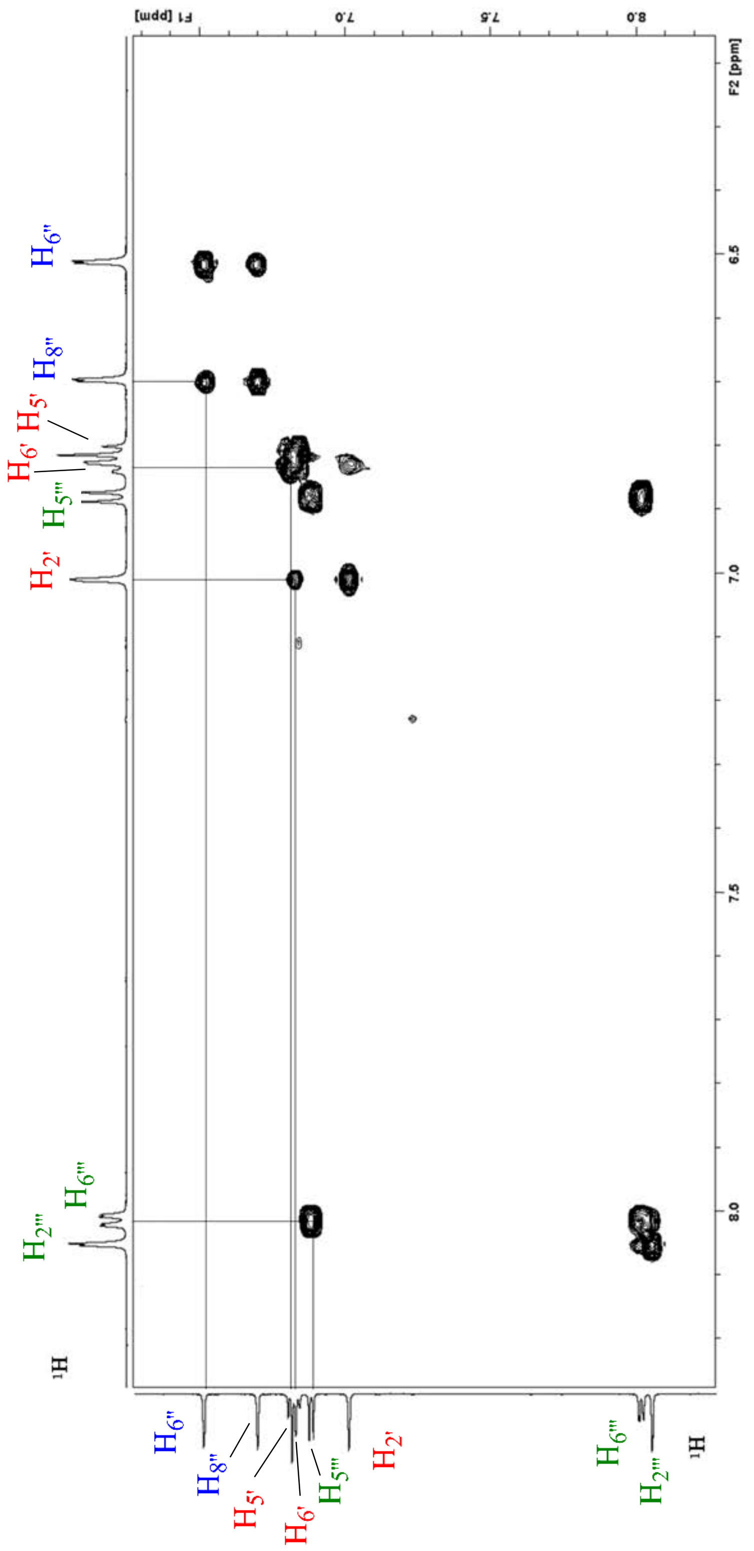

Figure S13-3: Expanded spectrum of COSY of **3** and observed correlations (^{1}H: 600 MHz, CD_3OD, rt).

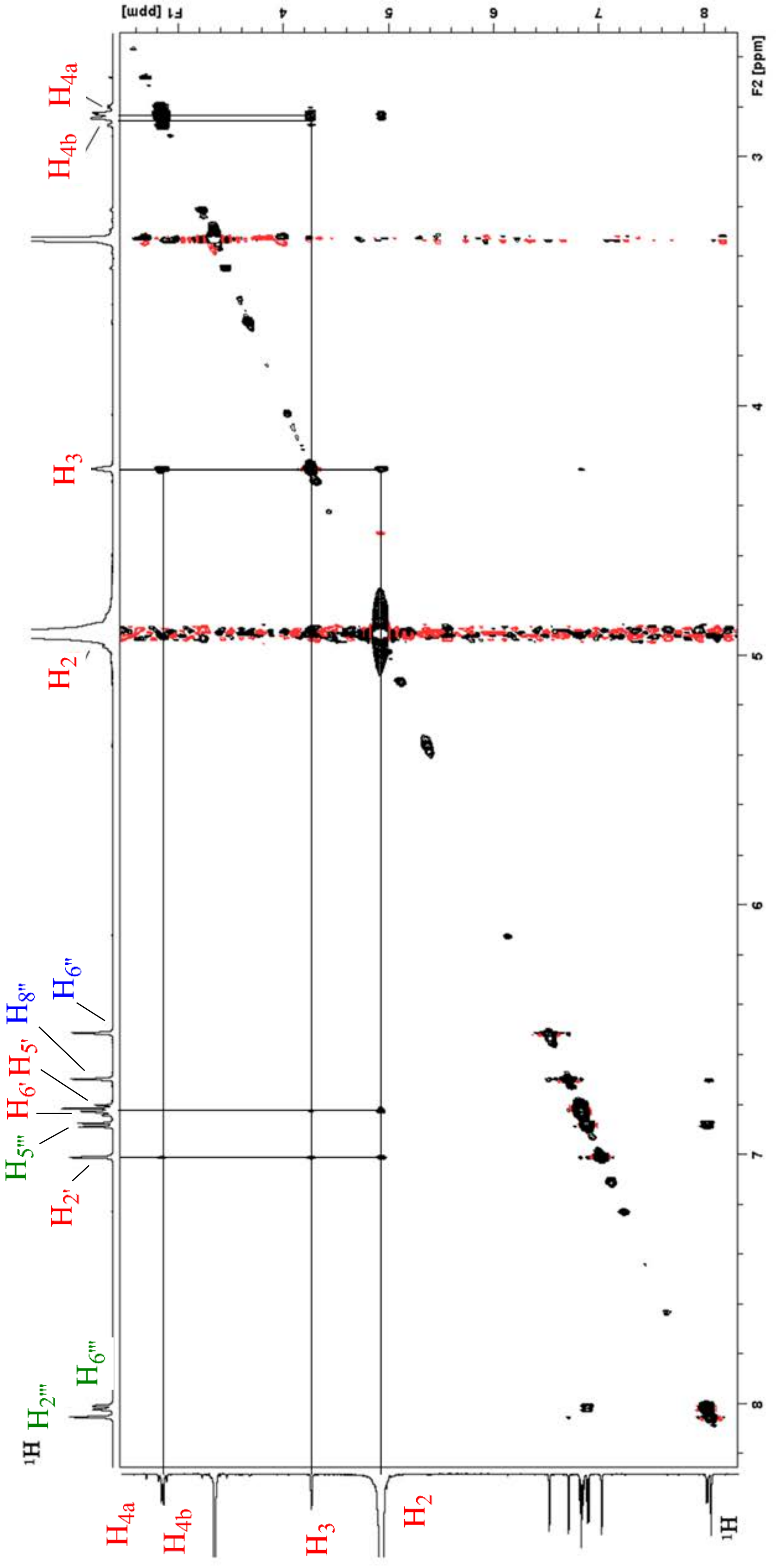

Figure S14-1: NOESY of **3** (^{1}H: 600 MHz, CD_3OD, rt).

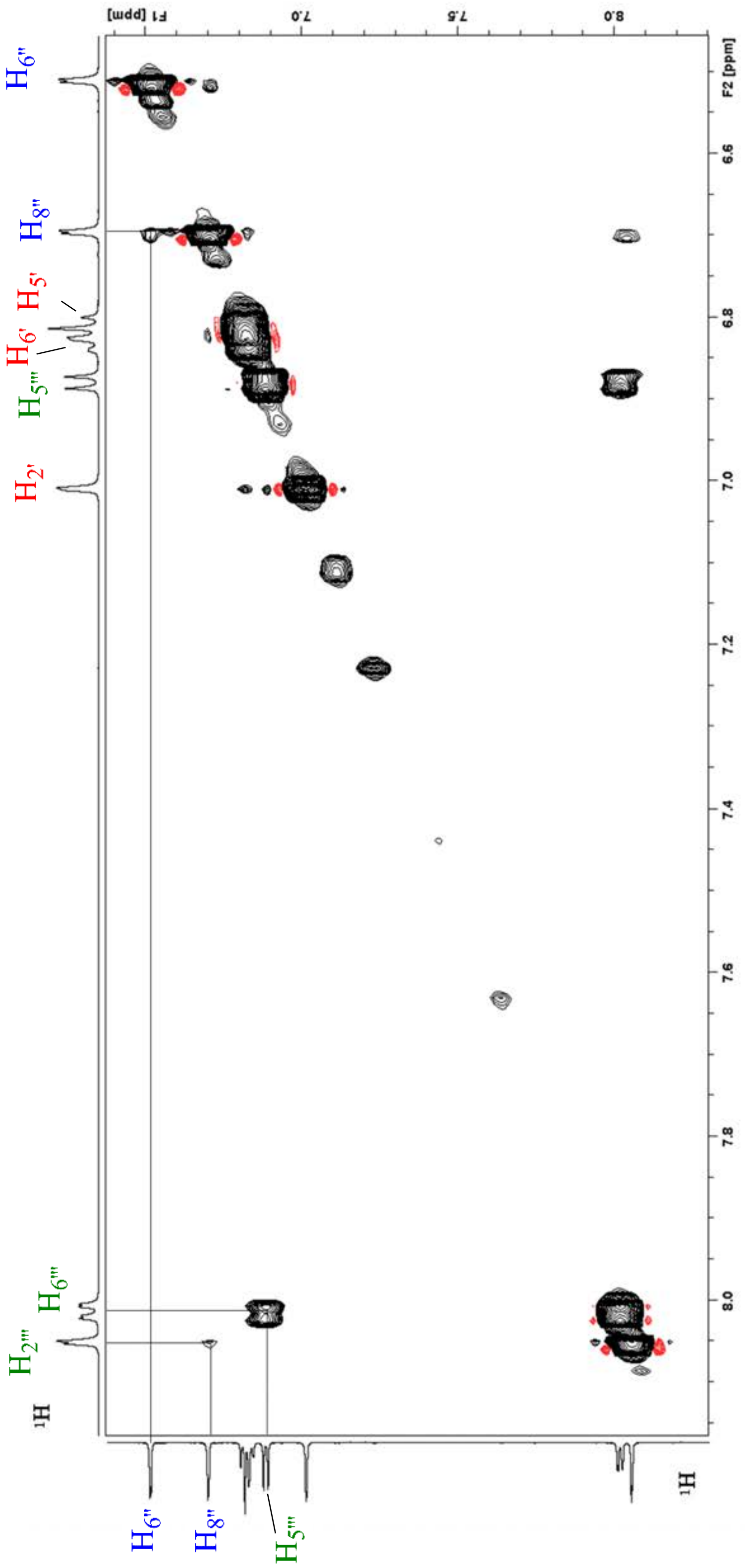

Figure S14-2: Expanded spectrum of NOESY of **3** and observed correlations (^1H: 600 MHz, CD_3OD, rt).

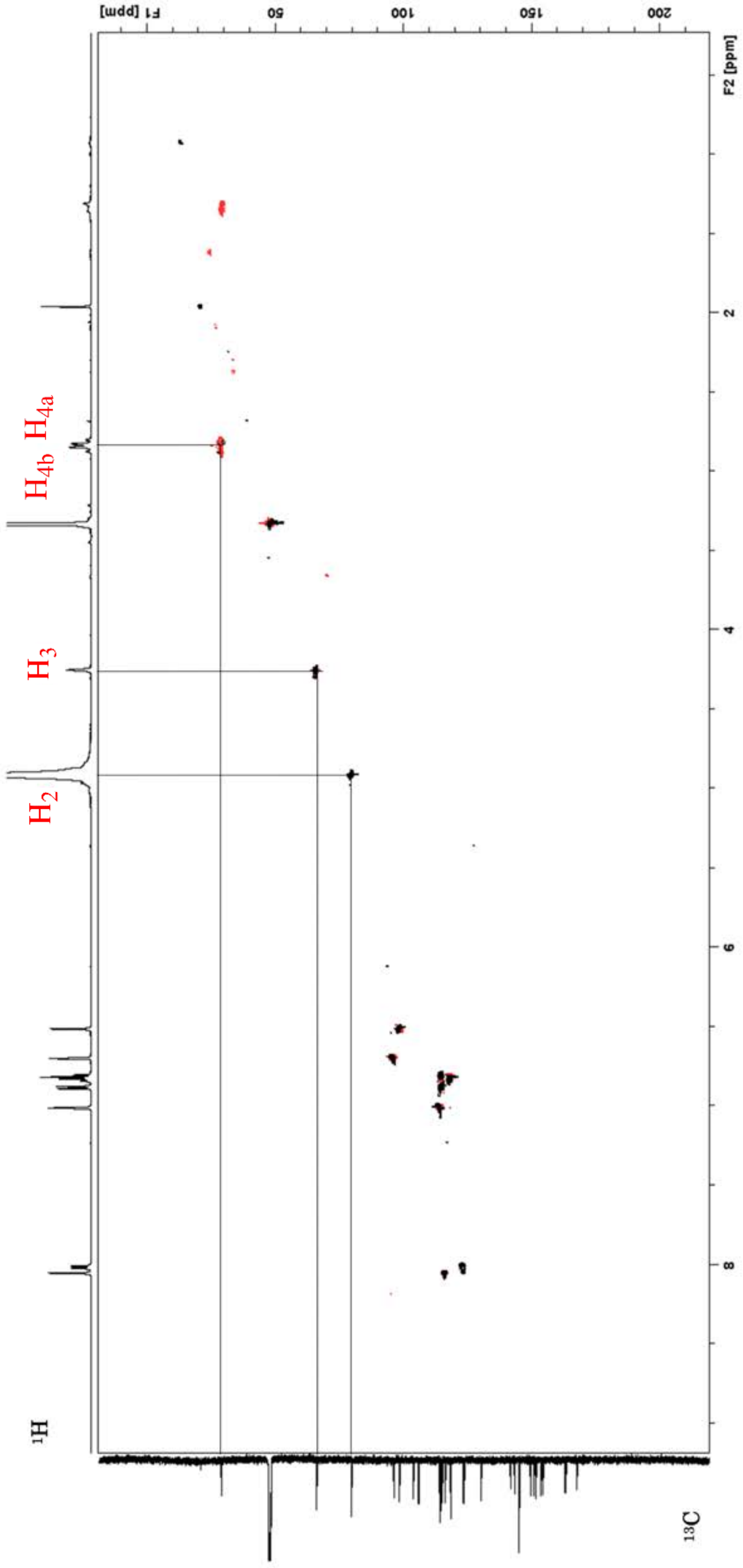

Figure S15-1: HSQC of **3** (^{1}H: 600 MHz, ^{13}C: 150 MHz, CD_3OD, rt).

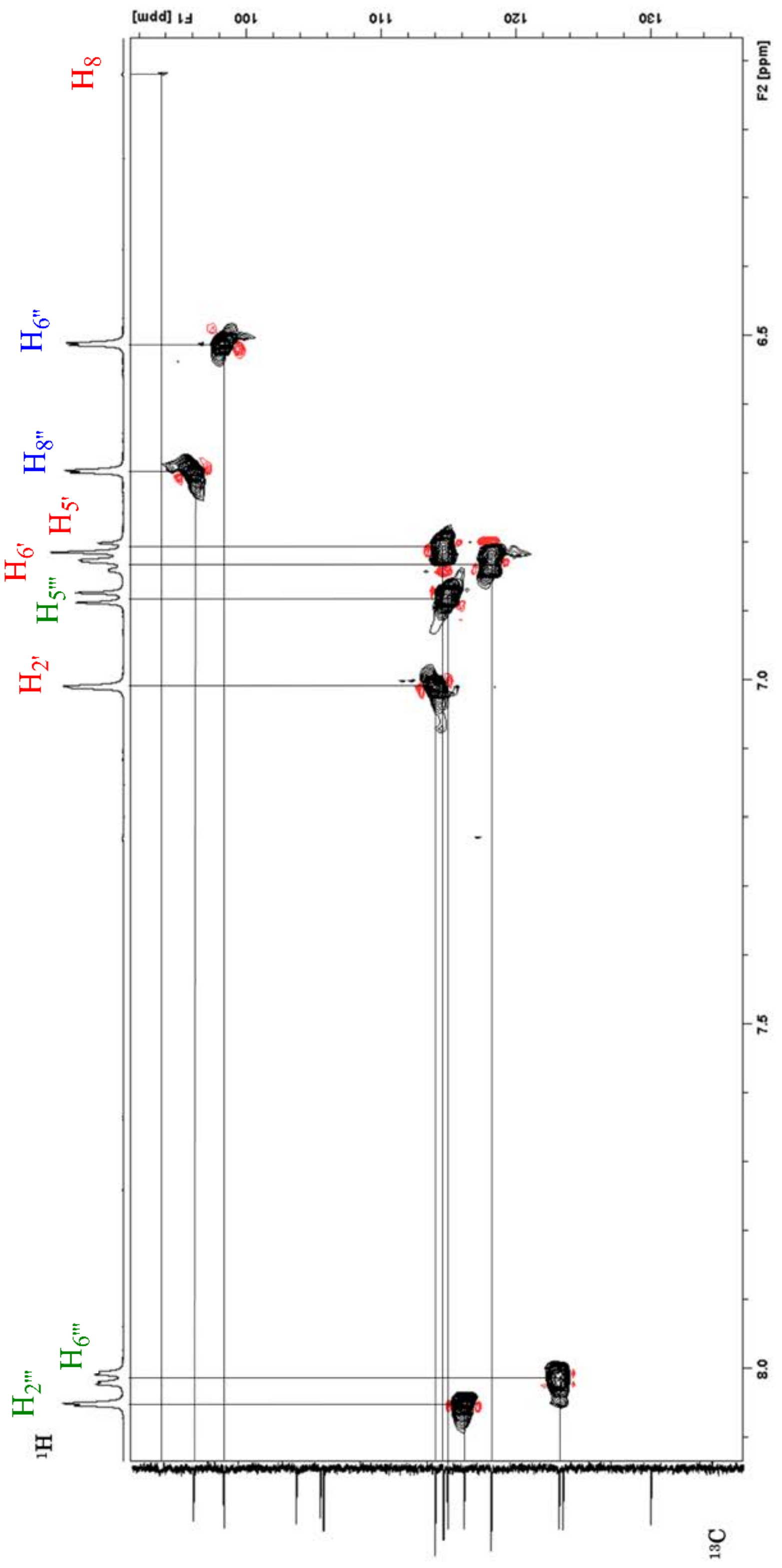

Figure S15-2: Expanded spectrum of HSQC of **3** (^{1}H: 600 MHz, ^{13}C: 150 MHz, CD_3OD, rt).

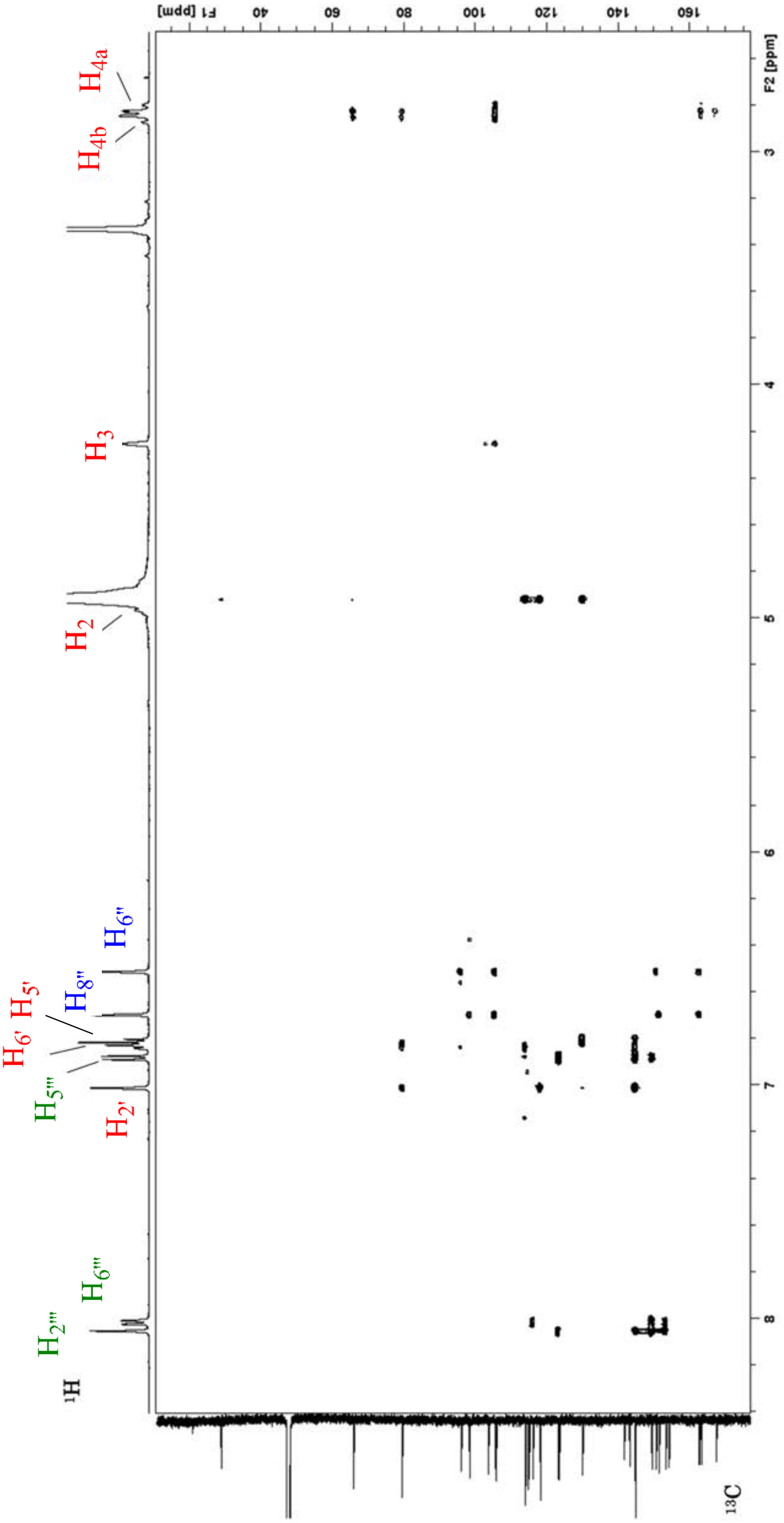

Figure S16-1: HMBC of **3** (^{1}H: 600 MHz, ^{13}C: 150 MHz, CD_3OD, rt).

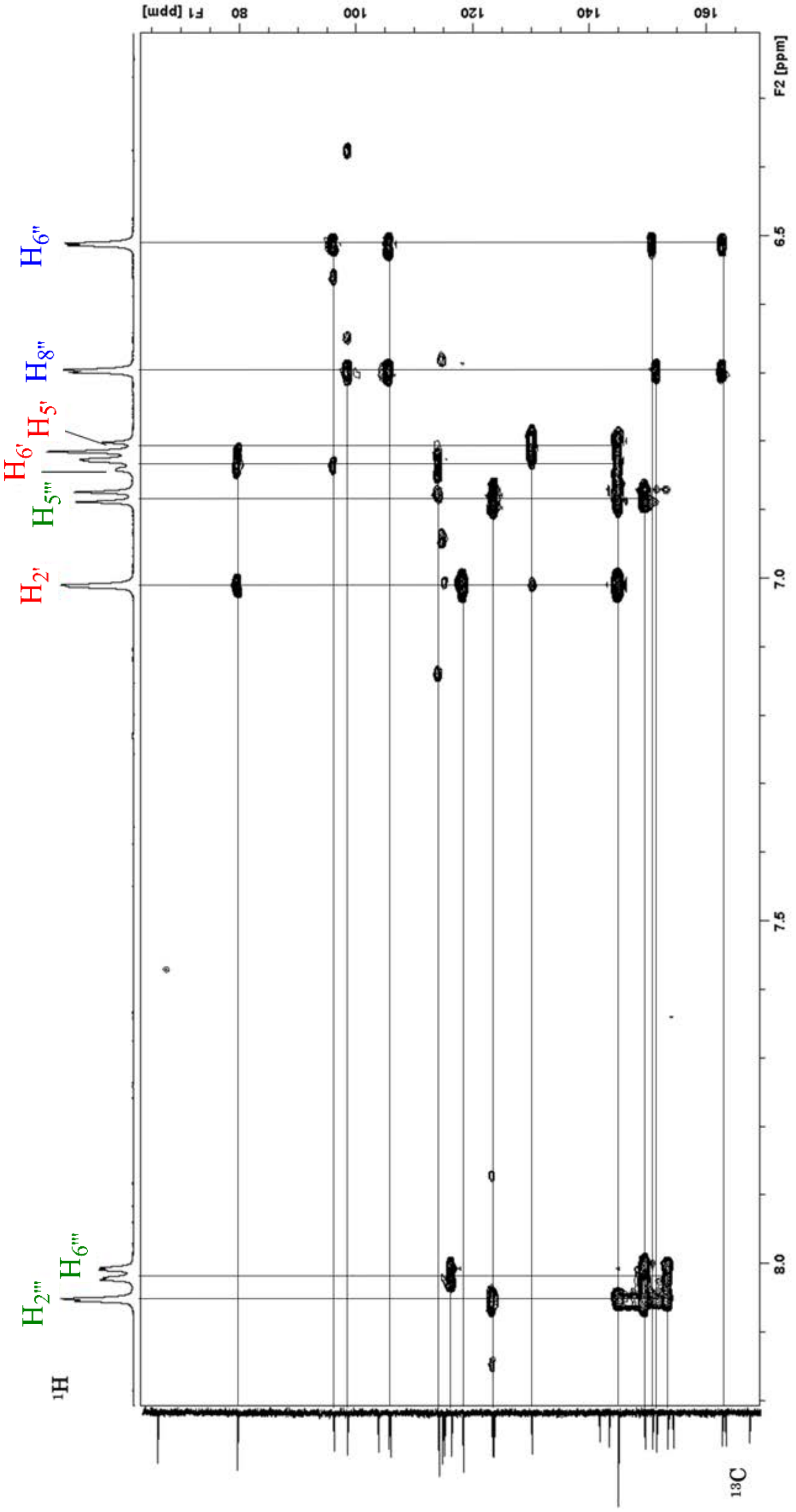

Figure S16-2: Expanded spectrum of HMBC of **3** and observed correlations (^{1}H: 600 MHz, ^{13}C: 150 MHz, CD_3OD, rt).

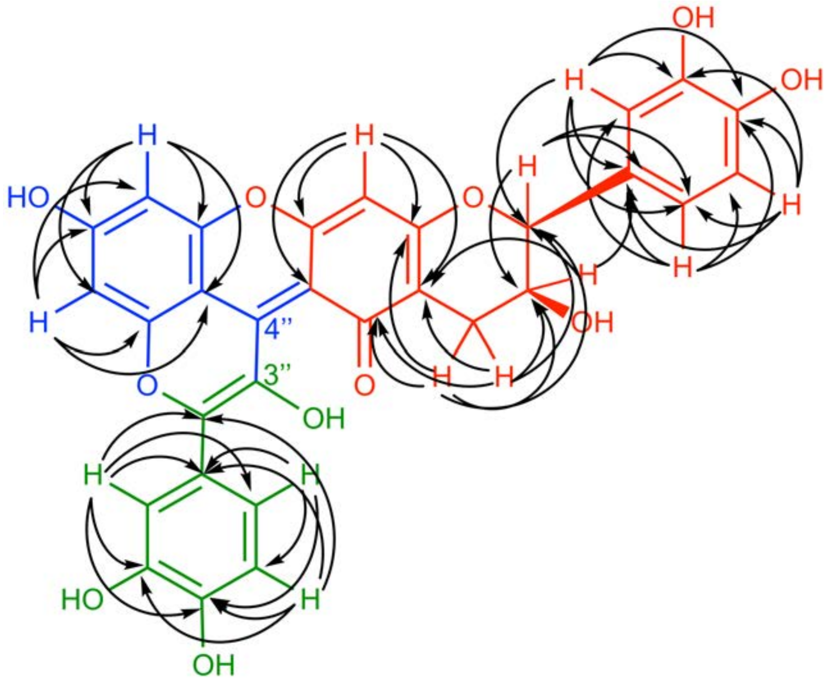

Figure S16-3: Observed HMBC correlations of **3**.

Photo-degraded catechinopyranocyanidin A (4)

(2*R*,3*S*)-2-(3,4-dihydroxyphenyl)-3,5,9-trihydroxy-6-oxo-2,3,4,6-tetrahydropyrano[3,2-*b*]xanthen-7-yl 3,4-dihydroxybenzoate

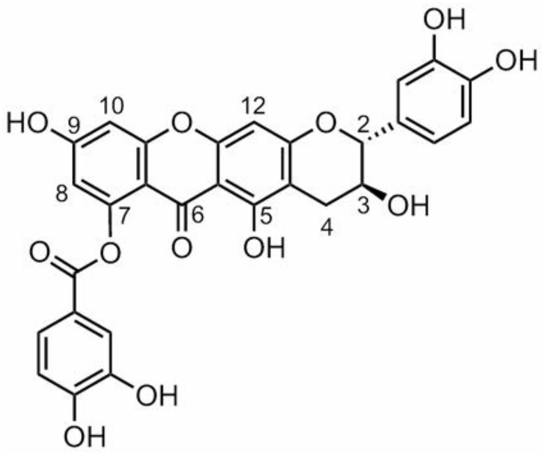

(The numbering of carbon atoms in the structure is different from that in the main text, but only for the IUPAC nomenclature)

ECD λ, (Δ*ε*): 351 nm (–1.24), 318 nm (0.15), 286 nm (6.09), 256 nm (5.62) ($2.00x10^{-5}$M, MeOH), UV λ_{max} (*ε*): 315 nm (22,400), 258 nm (25,200), 242 nm (34,200) ($2.00x10^{-5}$M, MeOH), IR (KBr) ν_{max} cm^{-1}:
3373, 3202, 1656, 1614, 1445, 1398, 1292, 1178, 1131, 837, 802, 723, HR-MS (ESI) Calcd for $C_{29}H_{21}O_{12}$ $[M+H]^+$: 561.1028, Found: 561.1026.

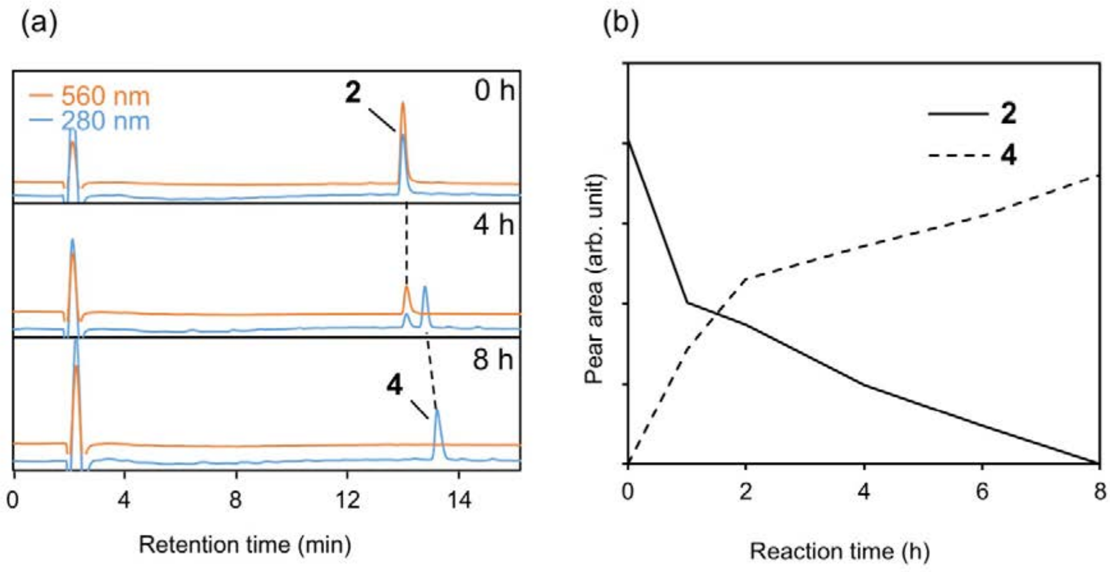

Figure S17: HPLC analysis of degradation reaction of **2** in MeOH (0.05 mM) by irradiation by fluorescent light (12 μW/cm2, 25°C). a: HPLC chromatogram of the reaction mixture with time course. B: Change of content of **2** and **4** in the reaction mixture analyzed with HPLC (det.: 280 nm).

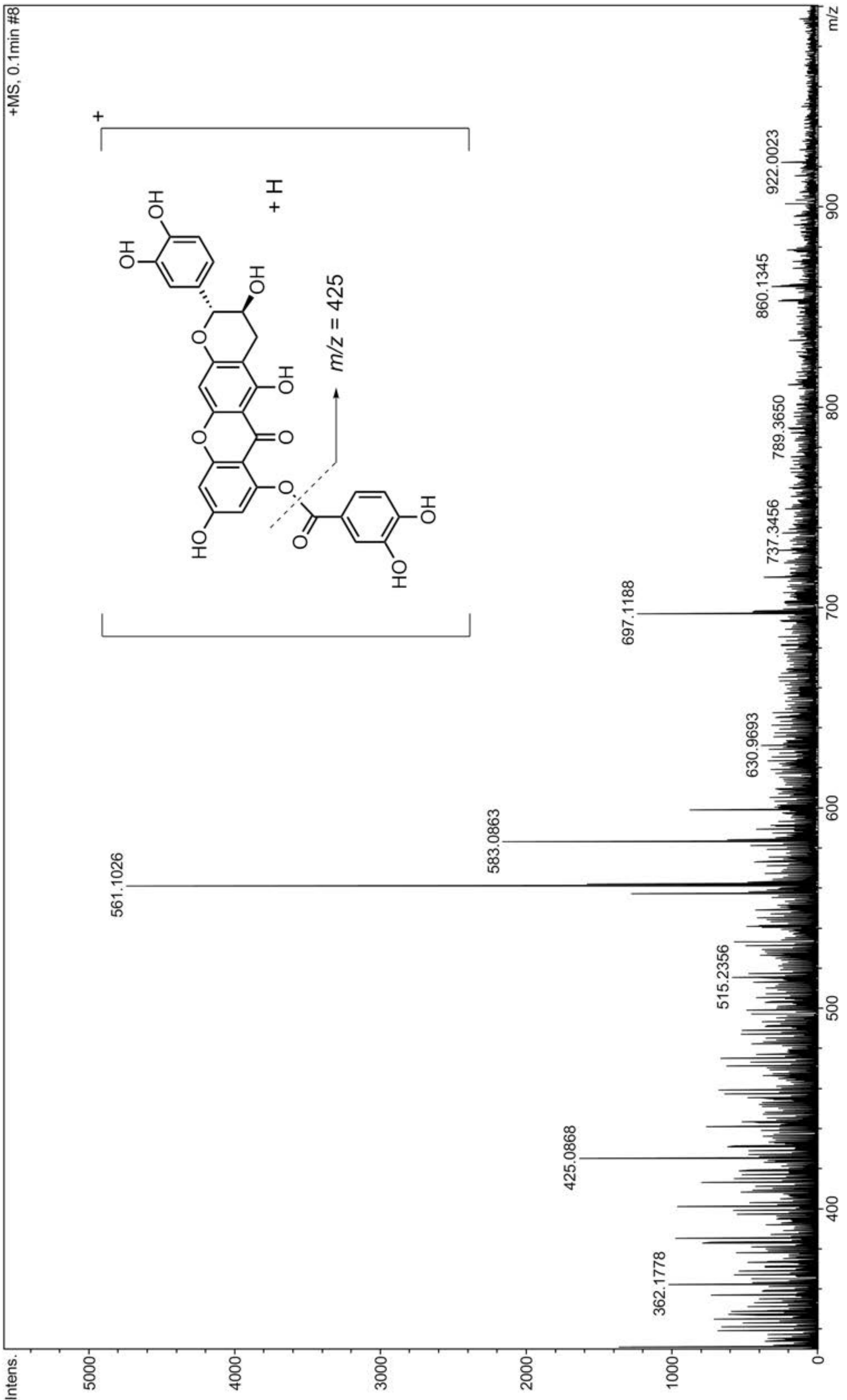

Figure S18: HR ESI-TOF MS of **4**.

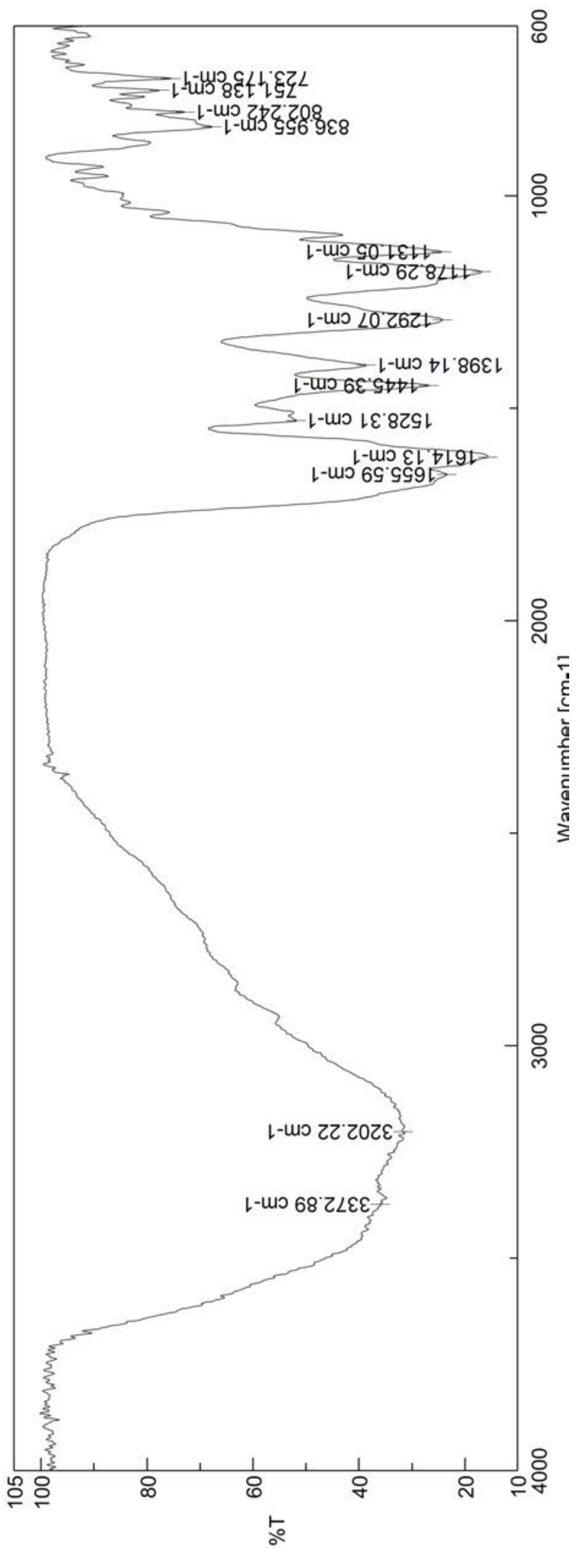

Figure S19: IR of **4**.

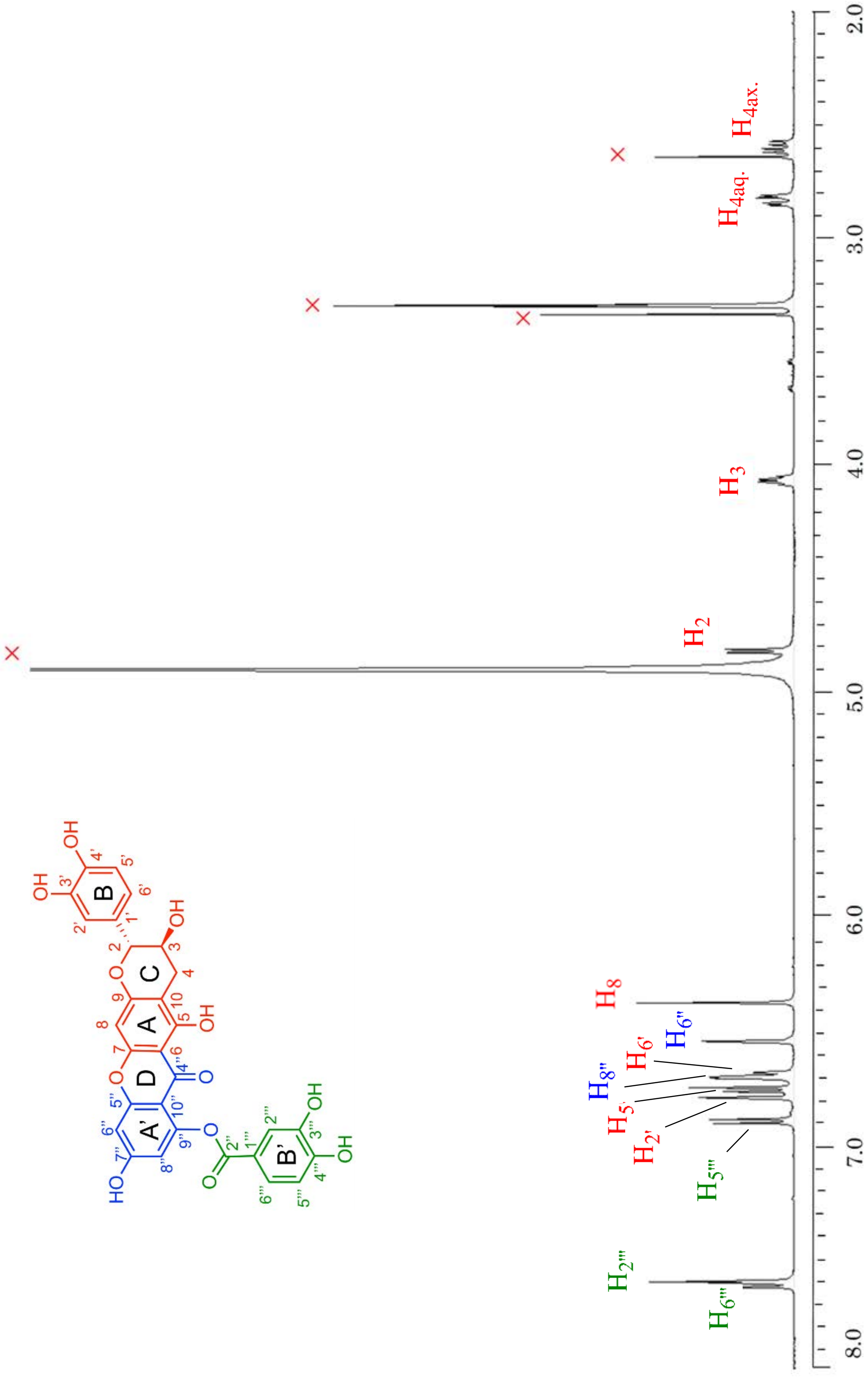

Figure S20: ^{1}H NMR of **4** (^{1}H: 500 MHz, CD_3OD, rt).

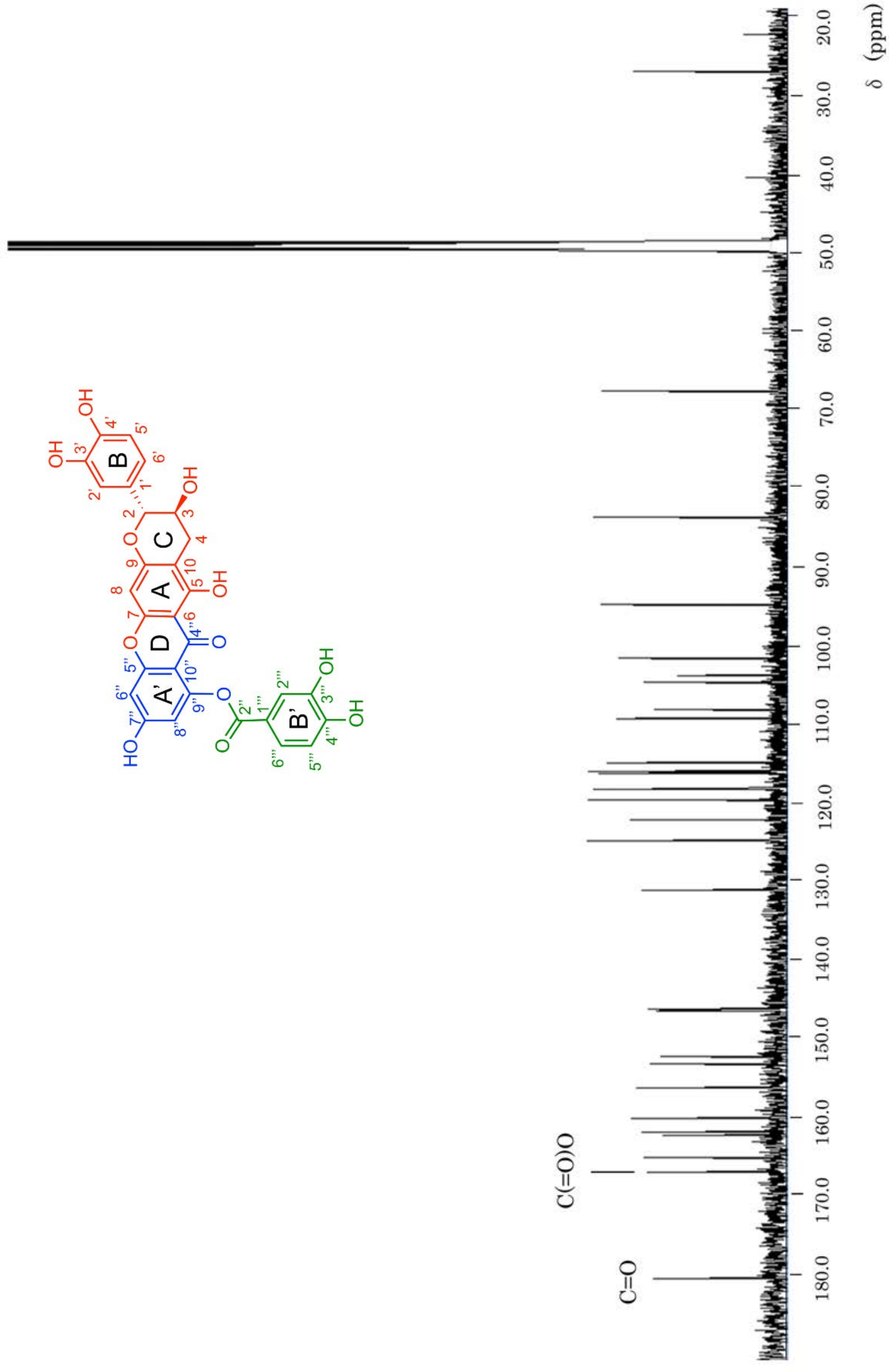

Figure S21: ^{13}C-NMR of **4** (^{13}C: 125 MHz, CD_3OD, rt).

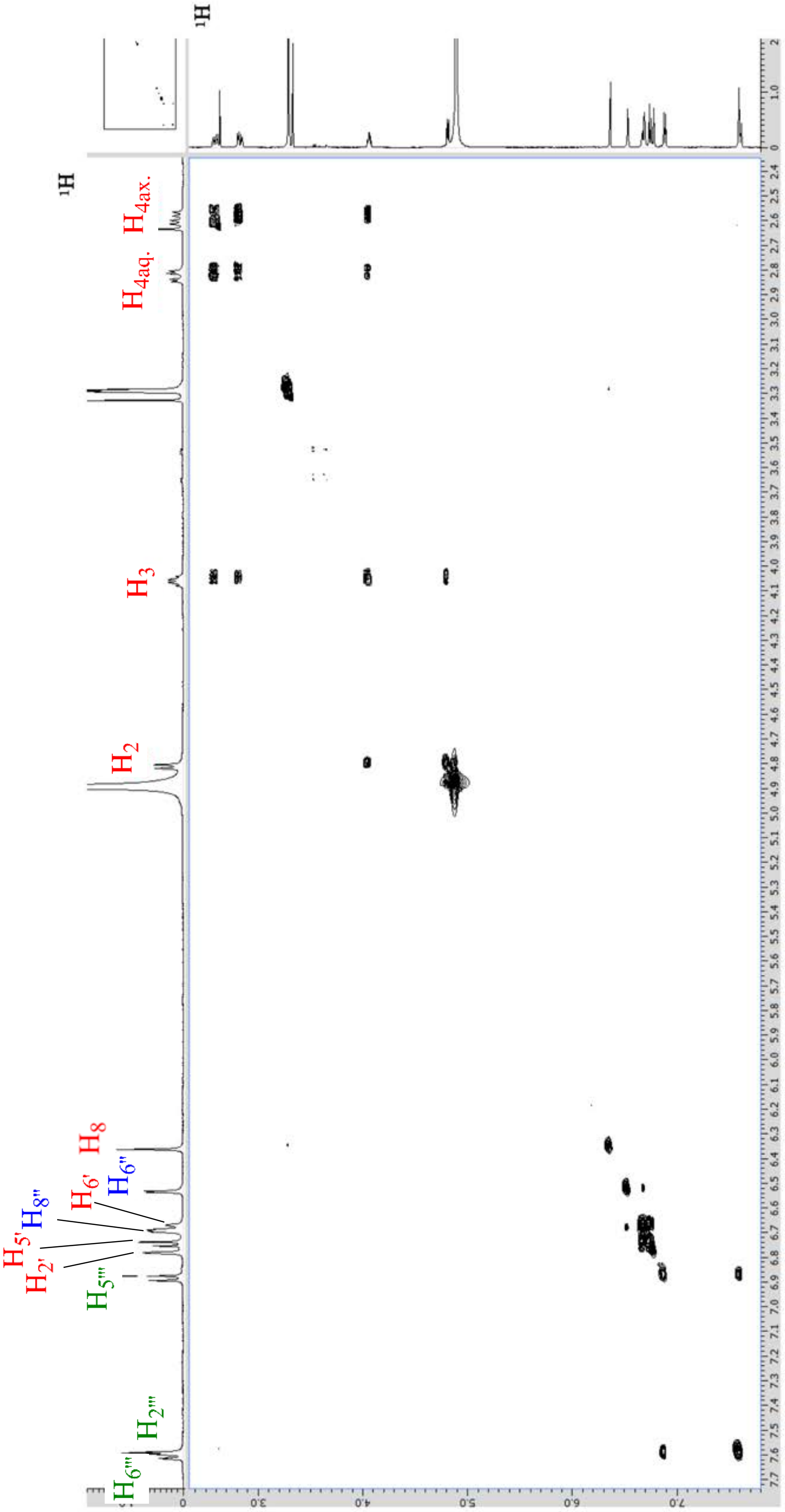

Figure S22-1: COSY of **4** (^{1}H: 500 MHz, CD_3OD, rt).

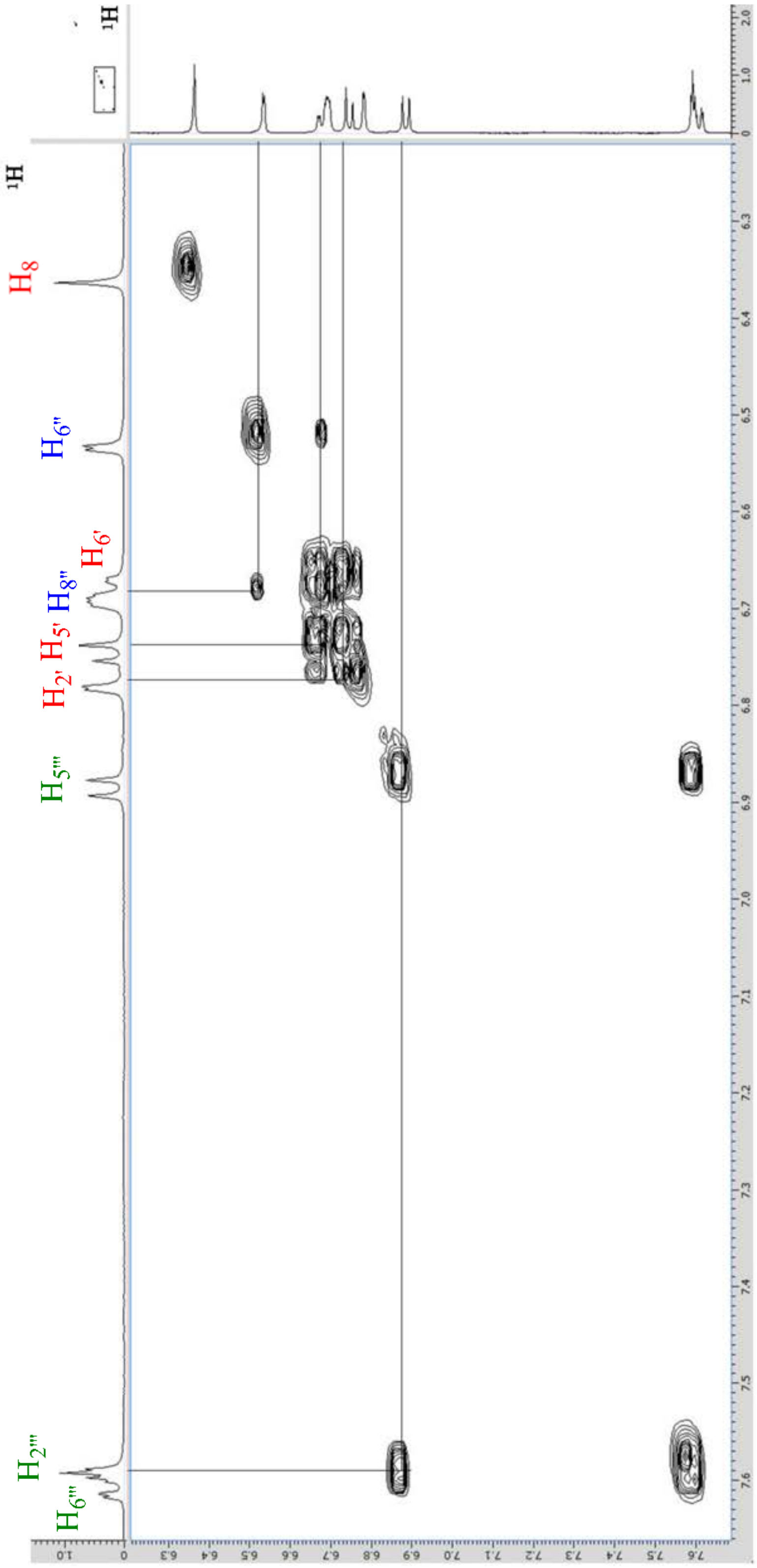

Figure S22-2: Expanded spectrum of COSY of **4** (^{1}H: 500 MHz, CD_3OD, rt).

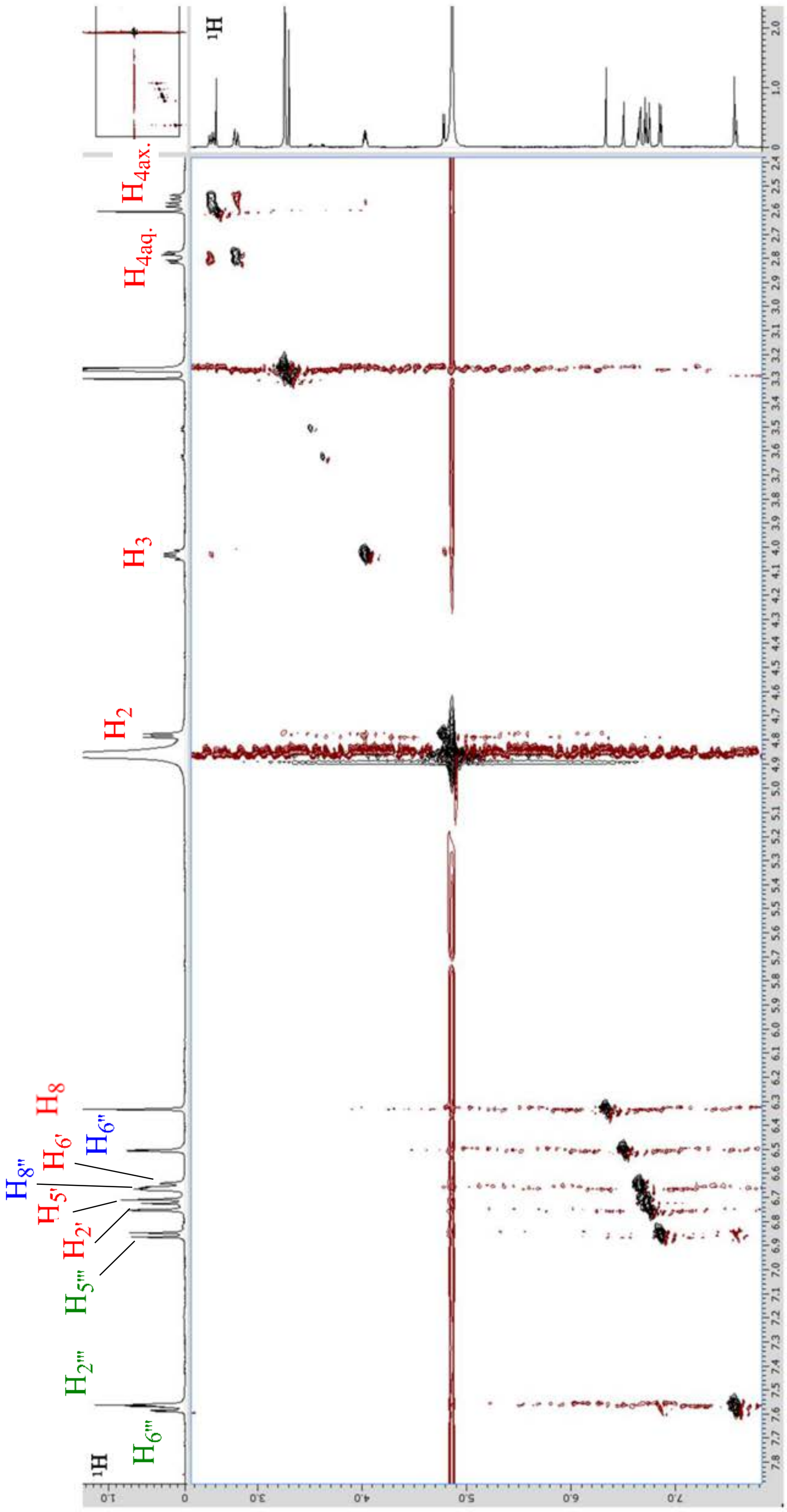

Figure S23-1: NOESY of **4** (^{1}H: 500 MHz, CD_3OD, rt).

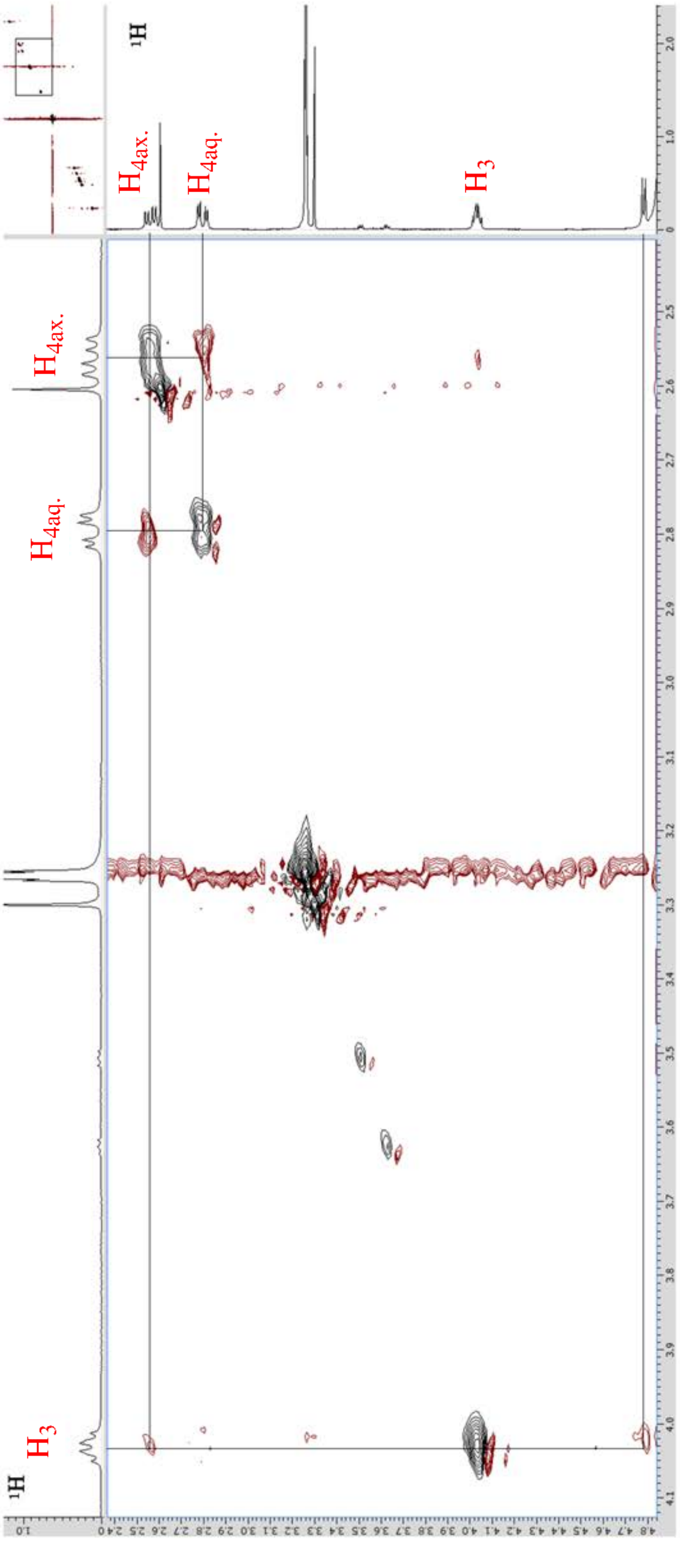

Figure S23-2: Expanded spectrum of NOESY of **4** and observed correlations (^{1}H: 500 MHz, CD_3OD, rt).

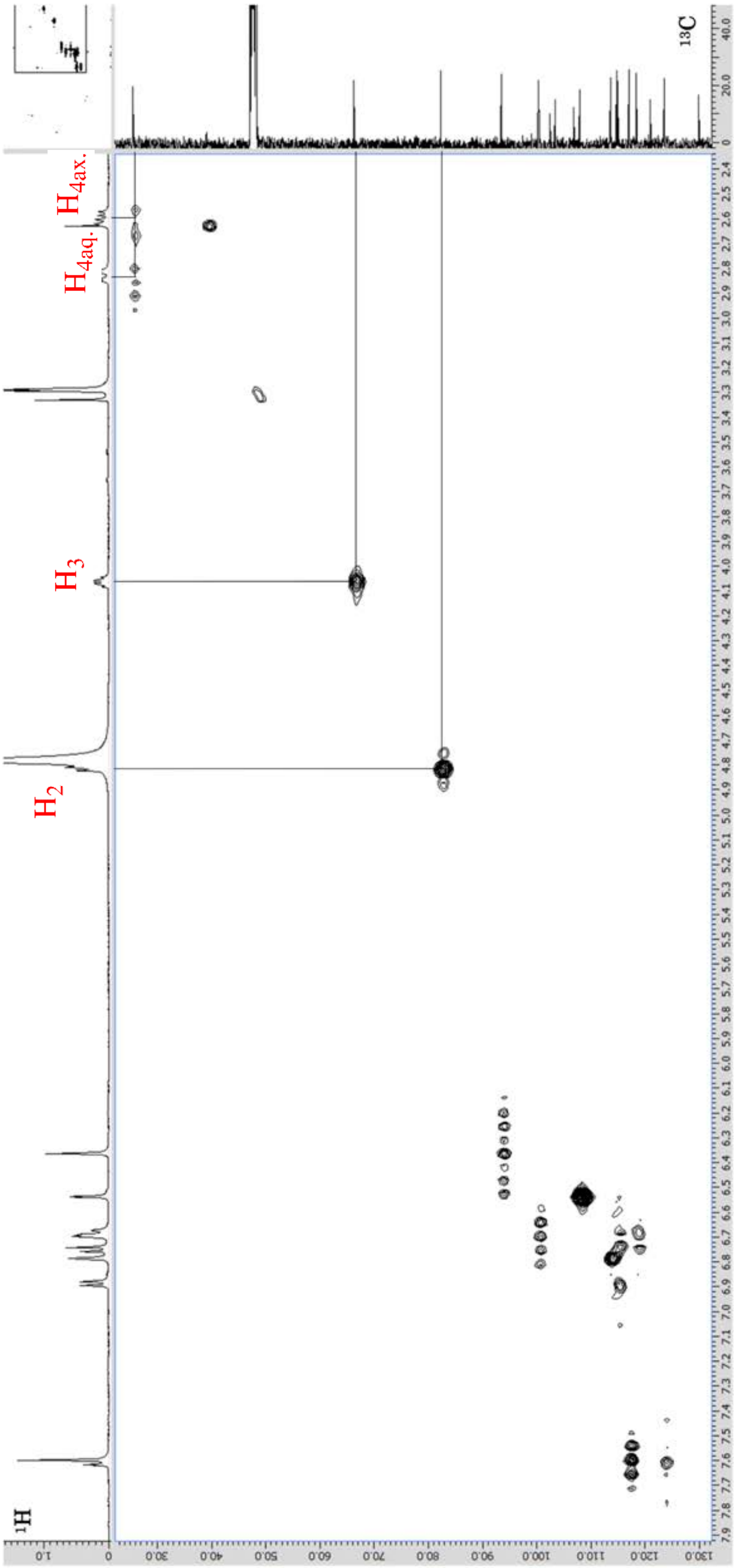

Figure S24-1: HSQC of **4** (^{1}H: 500 MHz, ^{13}C: 125 MHz, CD_3OD, rt).

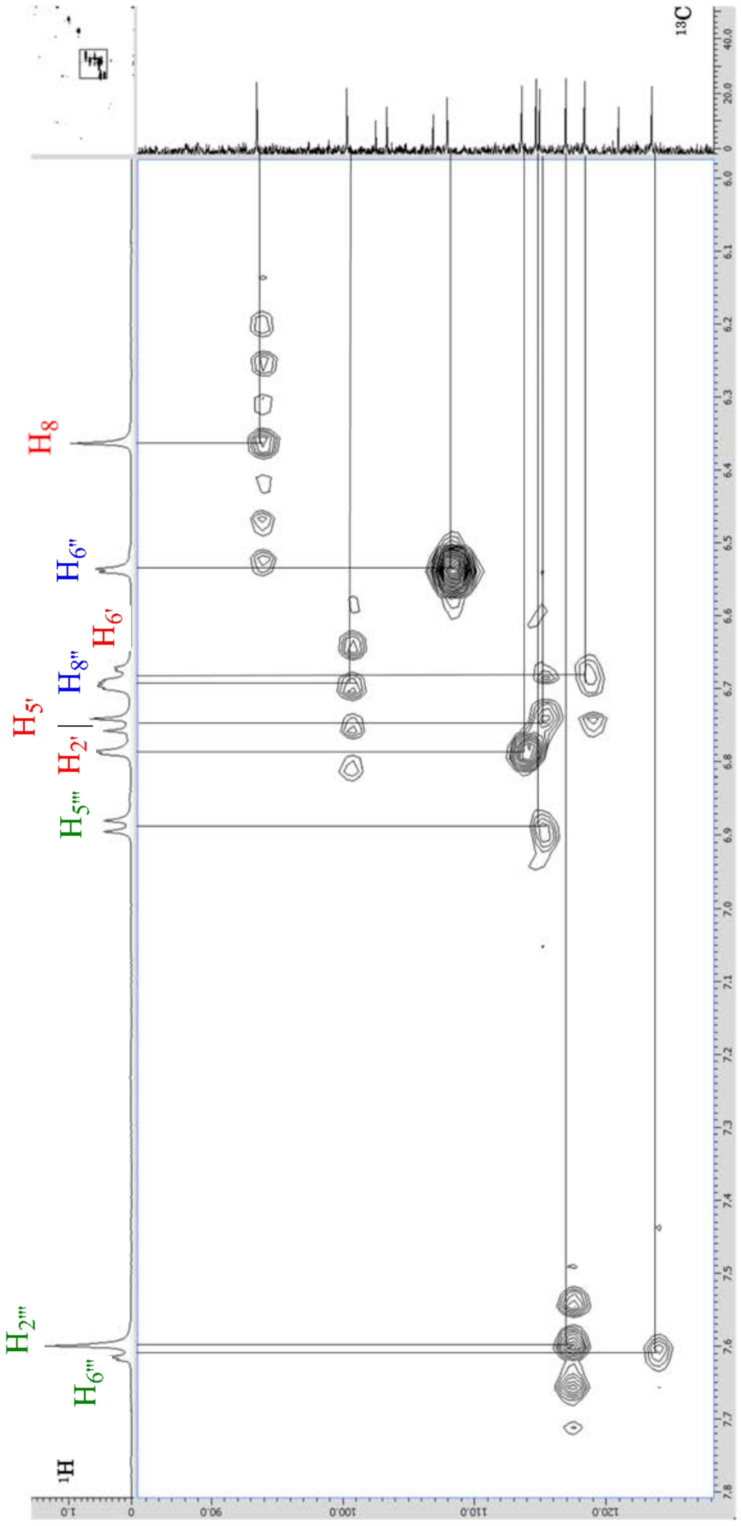

Figure S24-2: Expanded spectrum of HSQC of **4** and observed correlations (^{1}H: 500 MHz, ^{13}C: 125 MHz, CD_3OD, rt).

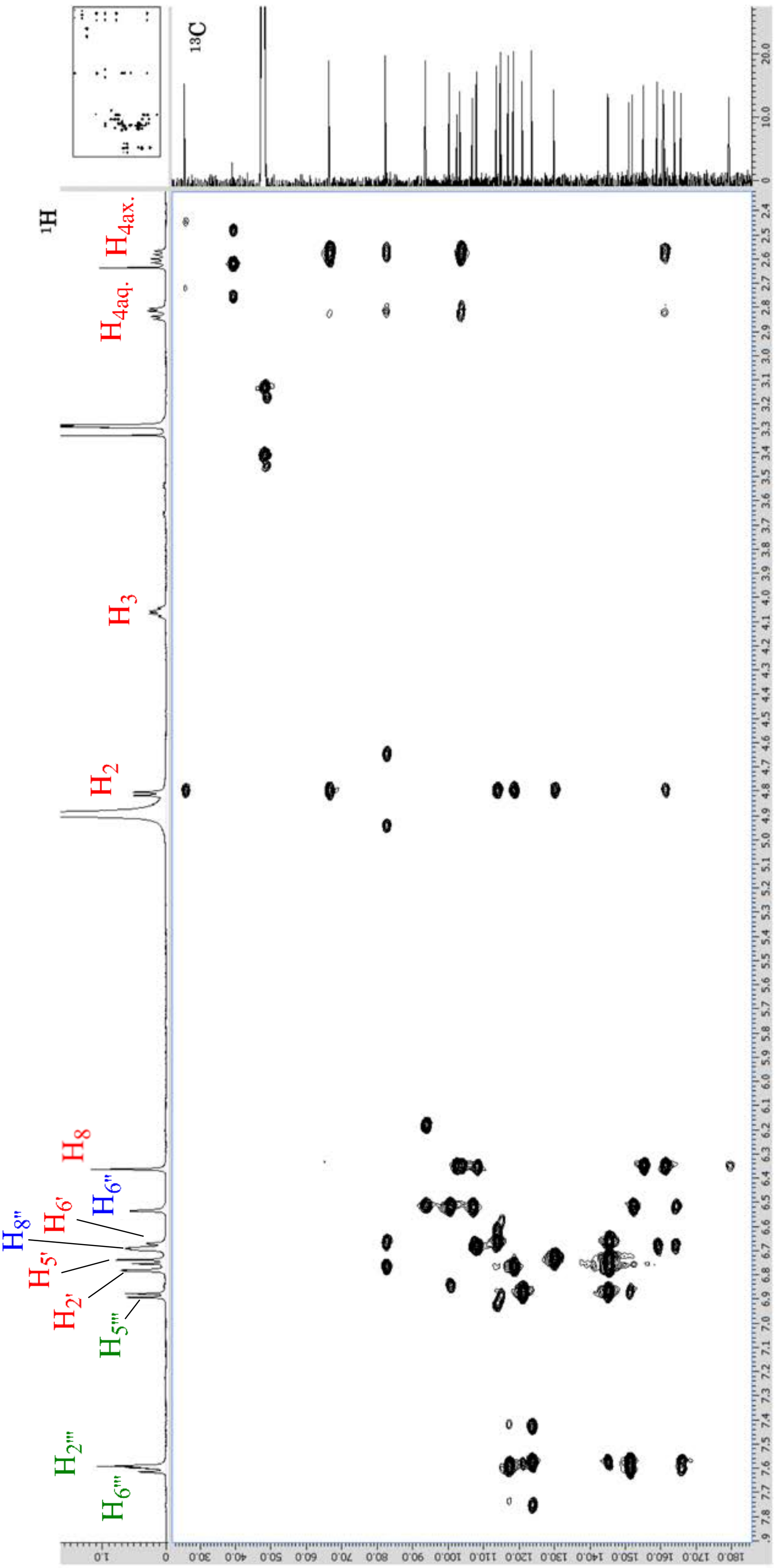

Figure S25-1: HMBC of **4** (^{1}H: 500 MHz, ^{13}C: 125 MHz, CD_3OD, rt).

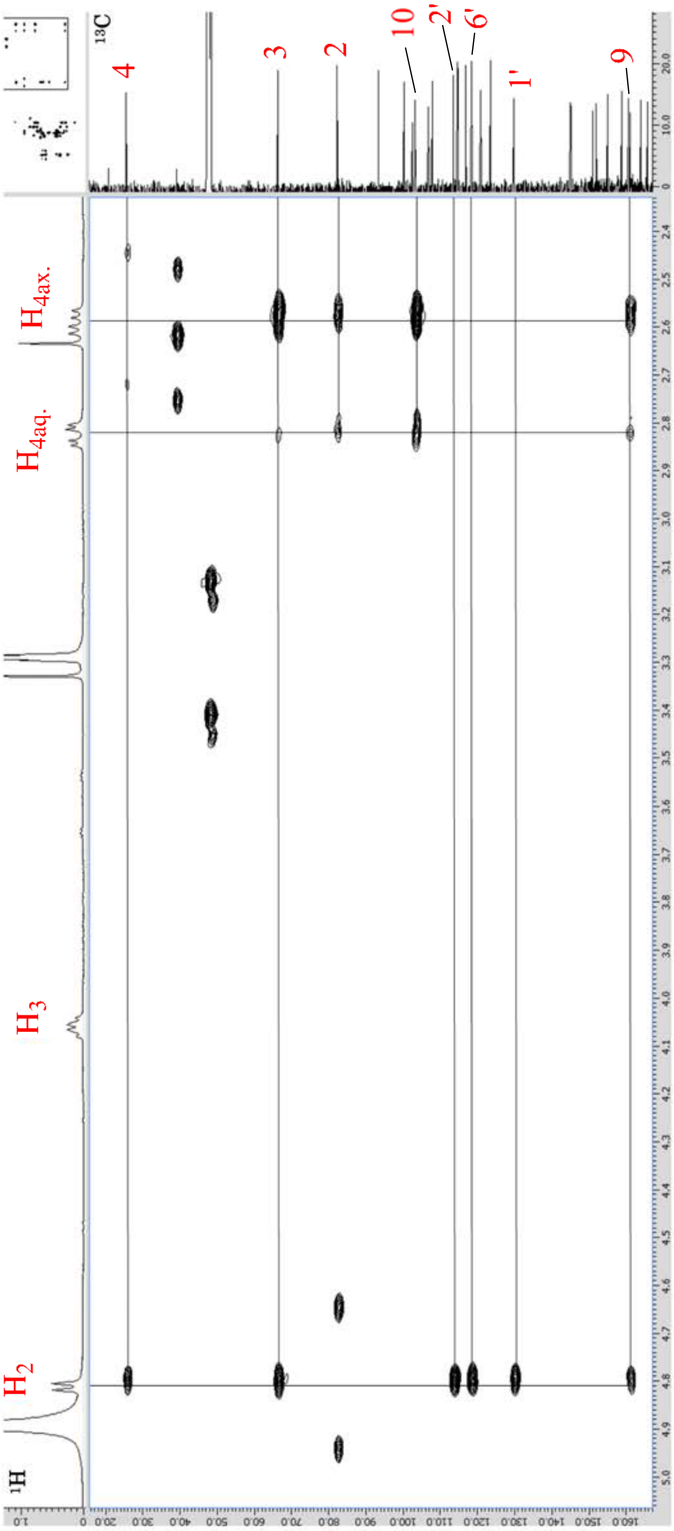

Figure S25-2: Expanded spectrum of HMBC of **4** and observed correlations (^{1}H: 500 MHz, ^{13}C: 125 MHz, CD_3OD, rt).

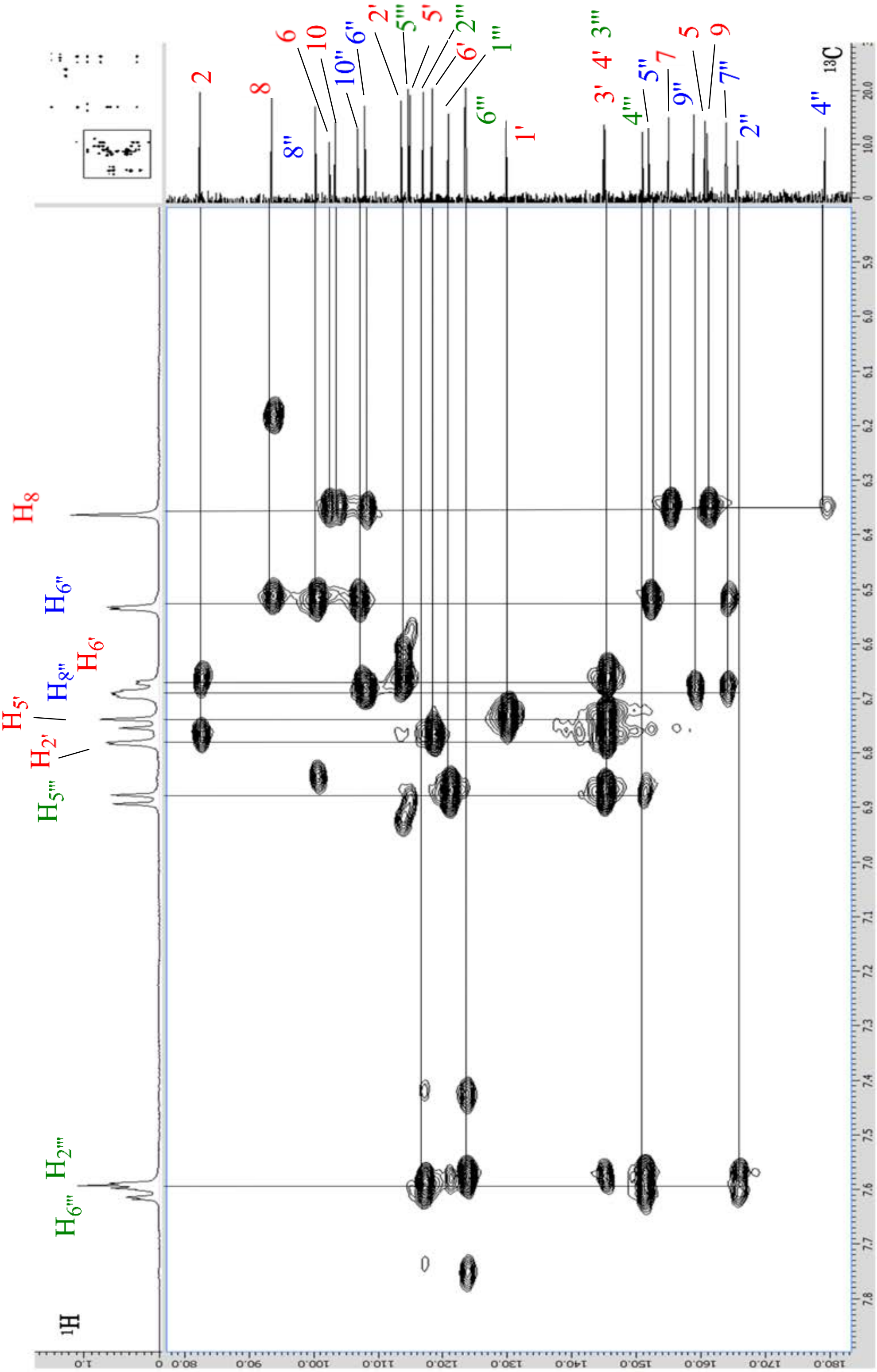

Figure S25-3: Expanded spectrum of HMBC of **4** and observed correlations (^{1}H: 500 MHz, ^{13}C: 125 MHz, CD_3OD, rt).

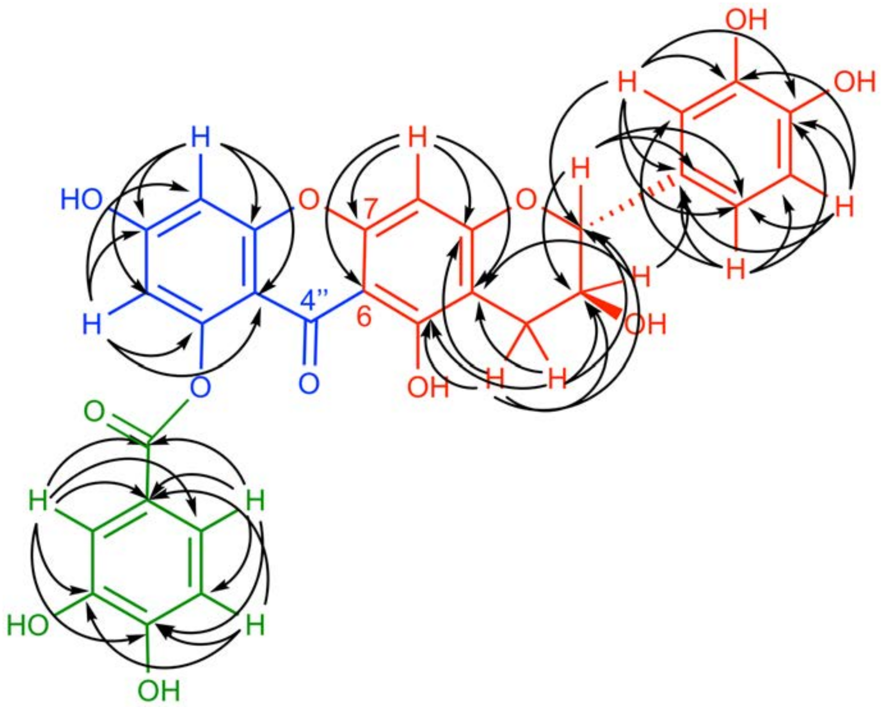

Figure S25-4: Observed HMBC correlations of **4**.

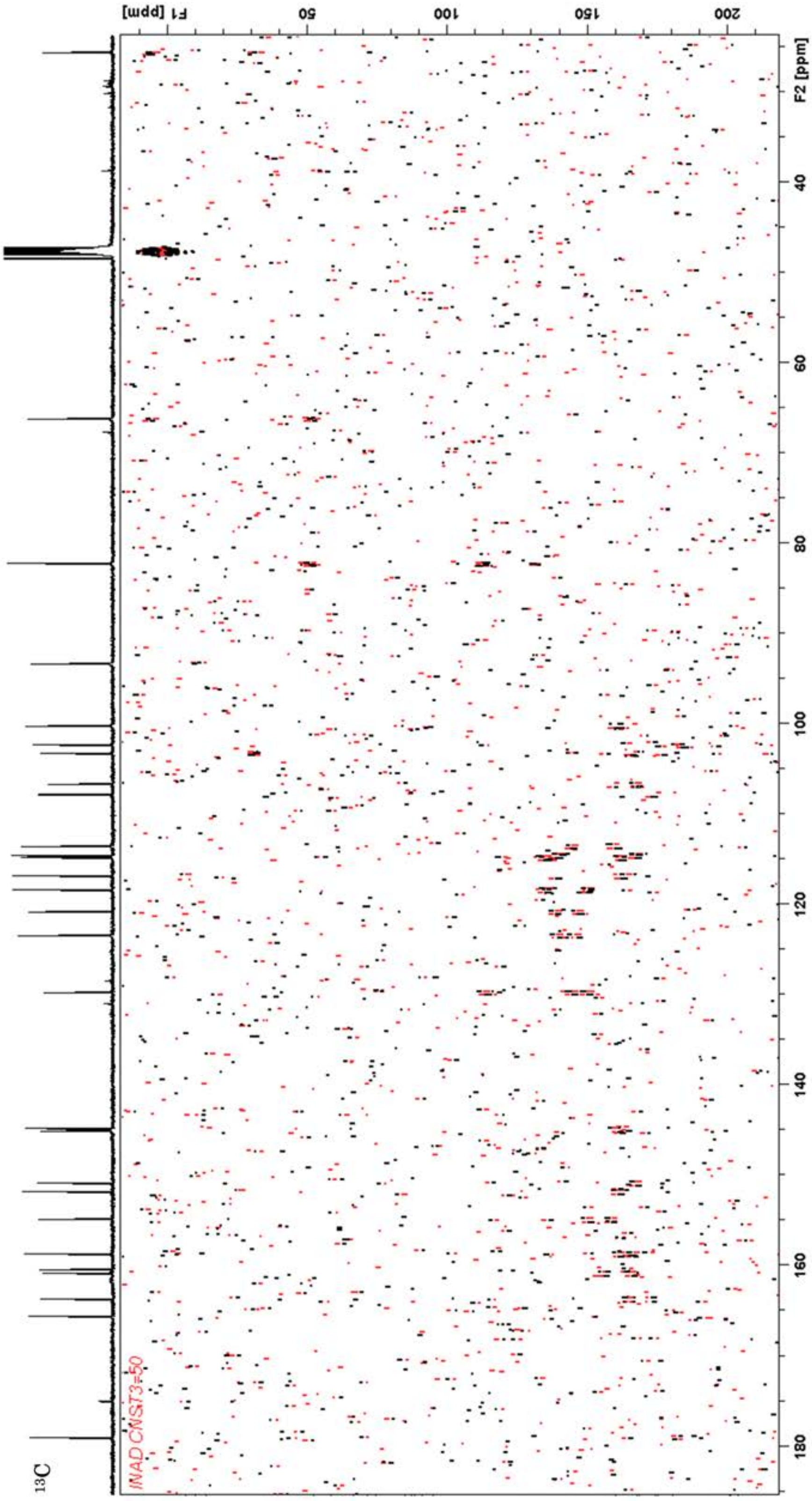

Figure S26-1: INADEQUATE of **4** (^{13}C: 150 MHz, CD_3OD, rt).

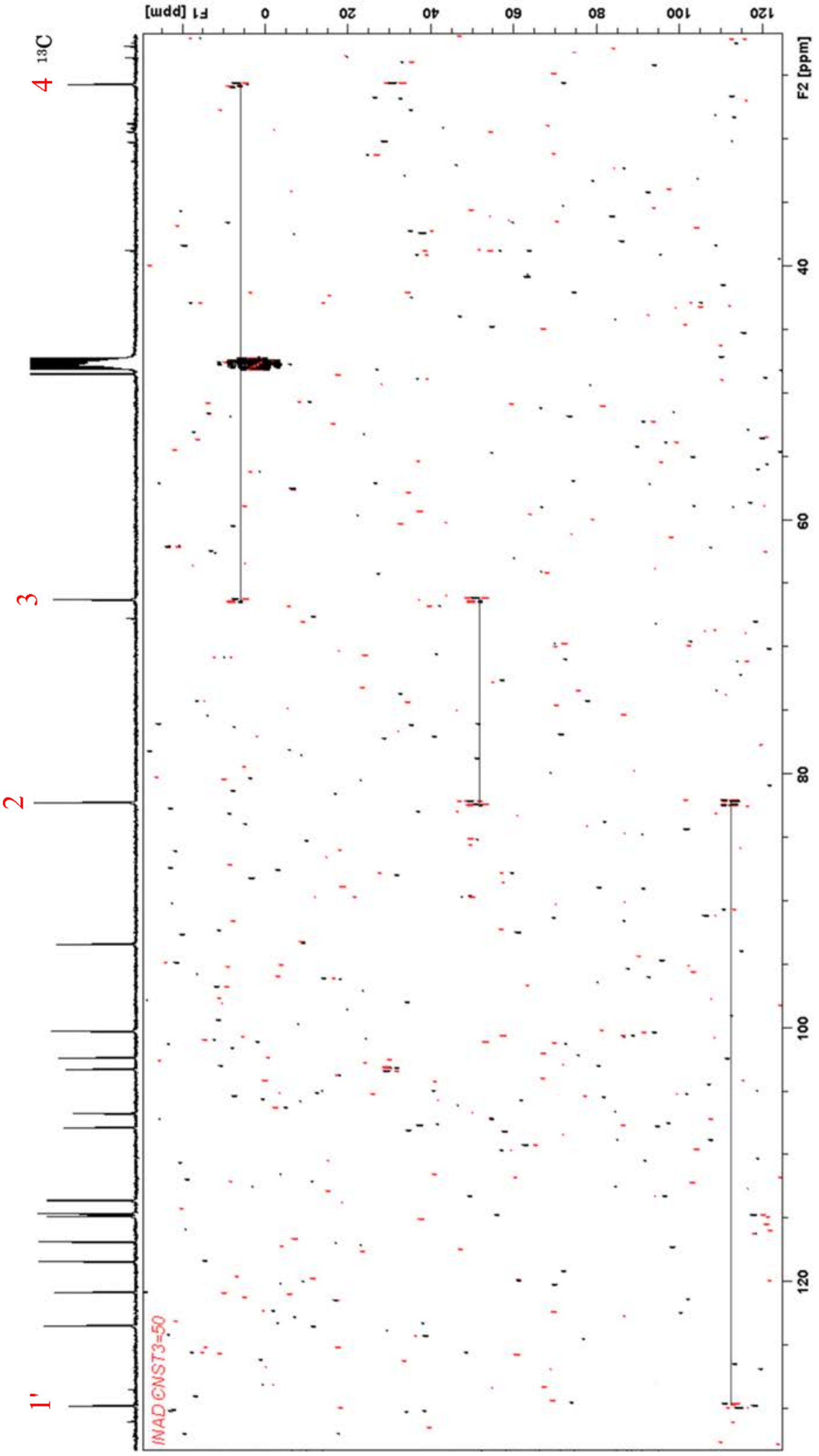

Figure S26-2: Expanded spectrum of INADEQUATE of **4** (^{13}C: 150 MHz, CD_3OD, rt).

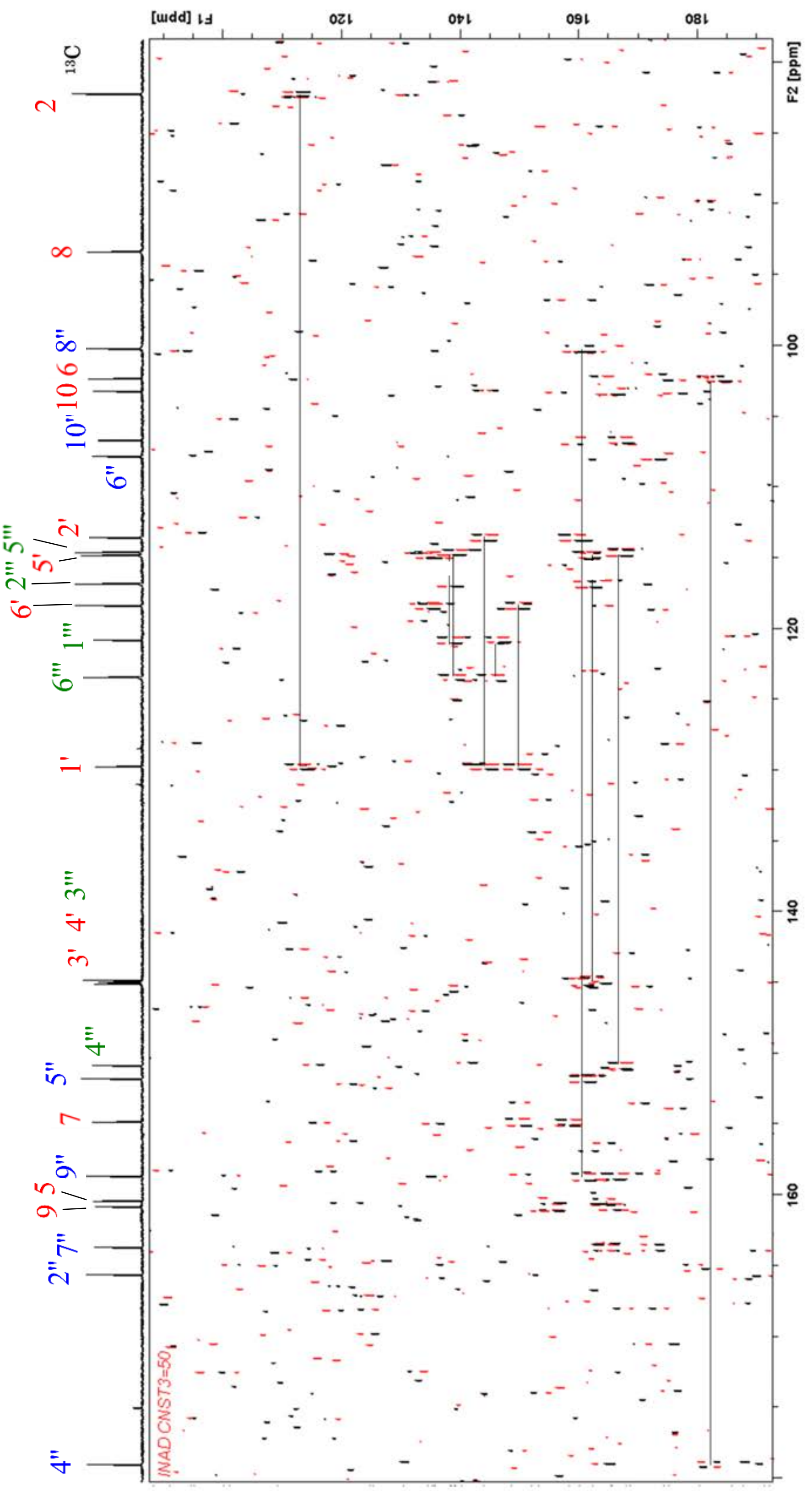

Figure S26-3: Expanded spectrum of INADEQUATE of **4** (^{13}C: 150 MHz, CD_3OD, rt).

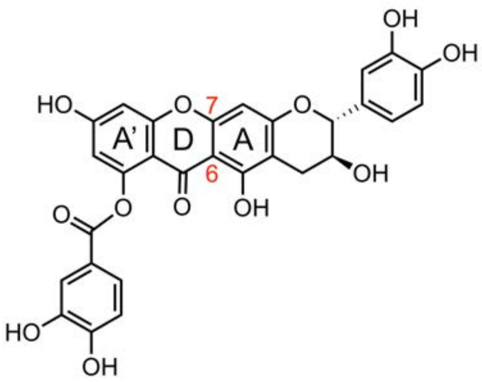

6-7 condensed pyrano-ring of **4**

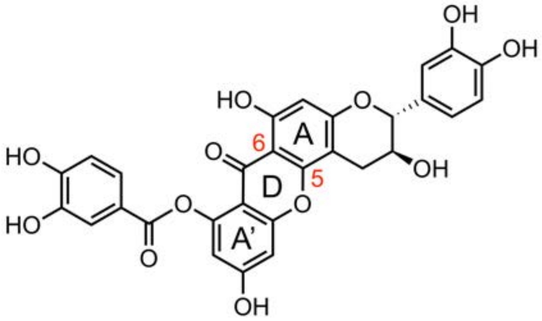

5-6 condensed pyrano-ring of **4**

Figure S27: Two candidate structure for **4**.

Photodegraded catechinopyranocyanidin B (5)

(2*S*,3*S*)-2-(3,4-dihydroxyphenyl)-3,5,9-trihydroxy-6-oxo-2,3,4,6-tetrahydropyrano[3,2-*b*]xanthen-7-yl 3,4-dihydroxybenzoate

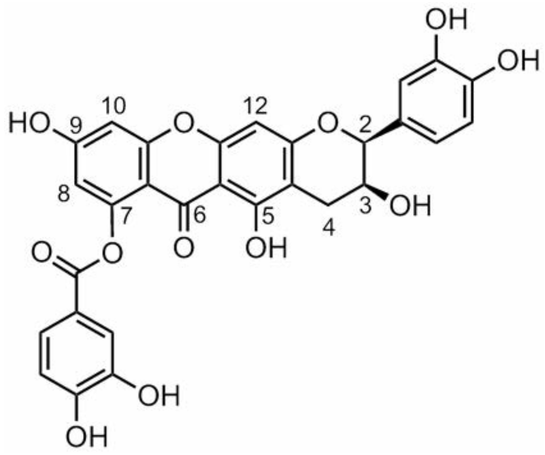

(The numbering of carbon atoms in the structure is different from that in the main text, but only for the IUPAC nomenclature)

ECD λ, (Δ*ε*): 355 nm (0.51), 316 nm (–2.65), 287 nm (–2.93), 258 nm (–3.75) (2.21 x10^{-5}M, MeOH), UV λ_{max} (*ε*): 316 nm (22,500), 258 nm (24,500), 242 nm (32,400) (2.21 x10^{-5}M, MeOH), IR (KBr) ν_{max} cm^{-1}: 3392, 3160, 1682, 1442, 1400, 1288, 1204, 1134, 839, 802, 724, HR-MS (ESI) Calcd for $C_{29}H_{21}O_{12}$ $[M+H]^+$: 561.1028, Found: 561.1025.

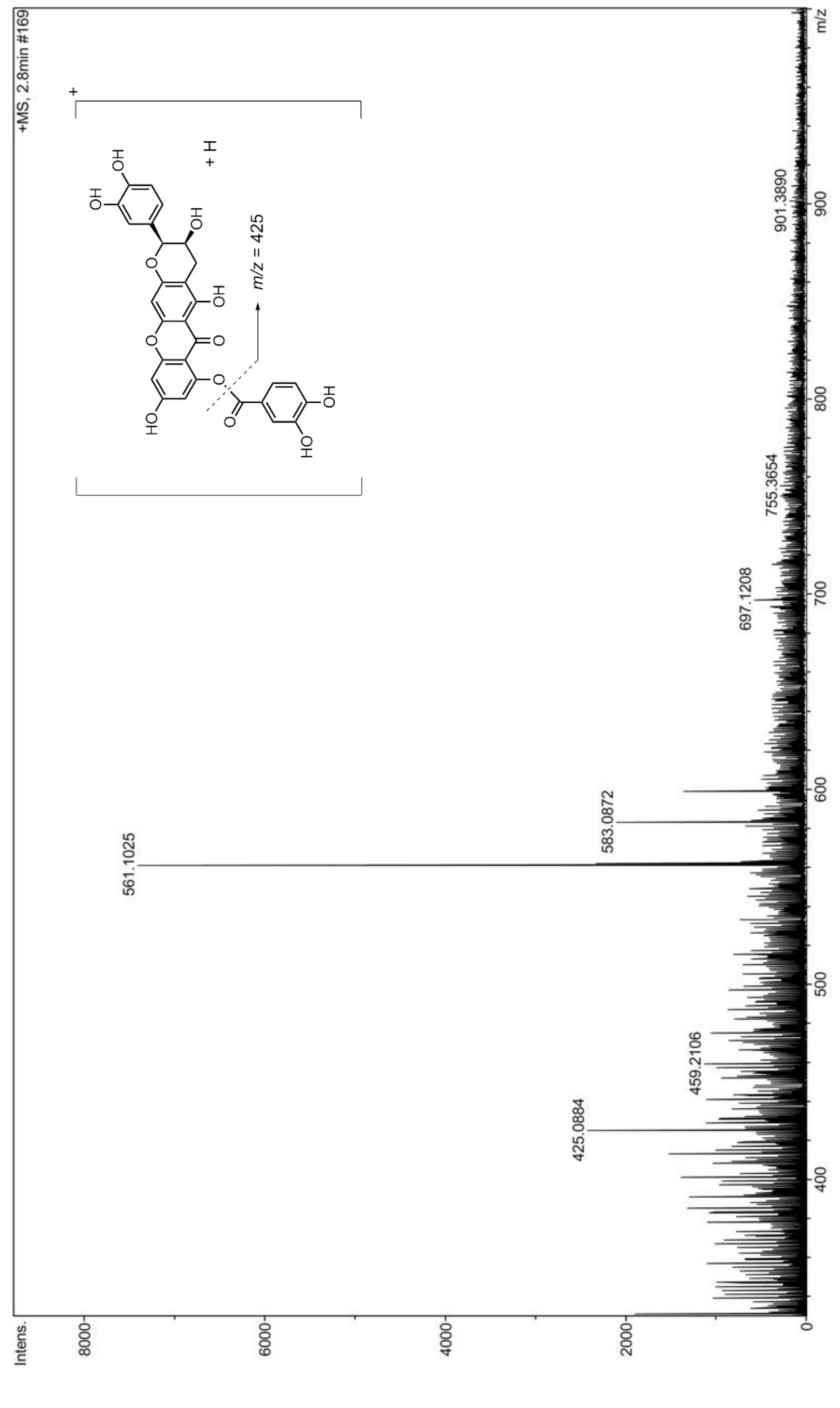

Figure S28: HR-ESI-TOF MS of **5**.

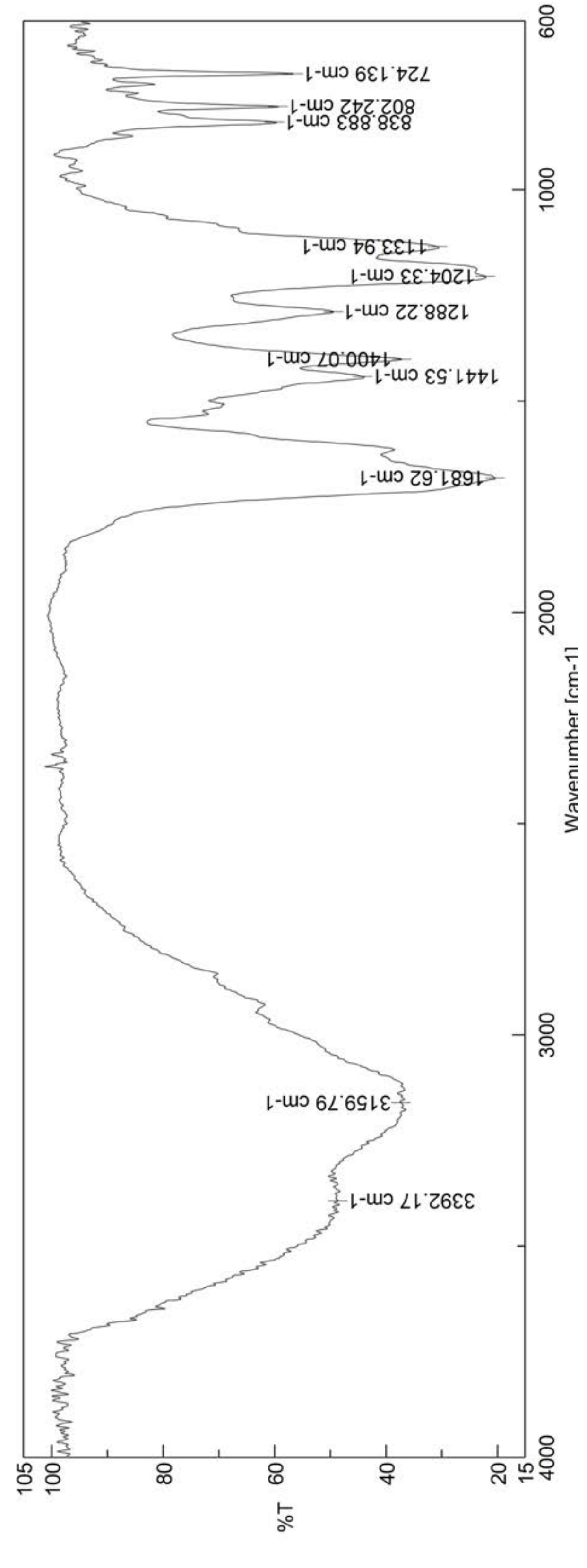

Figure S29: IR of **5**.

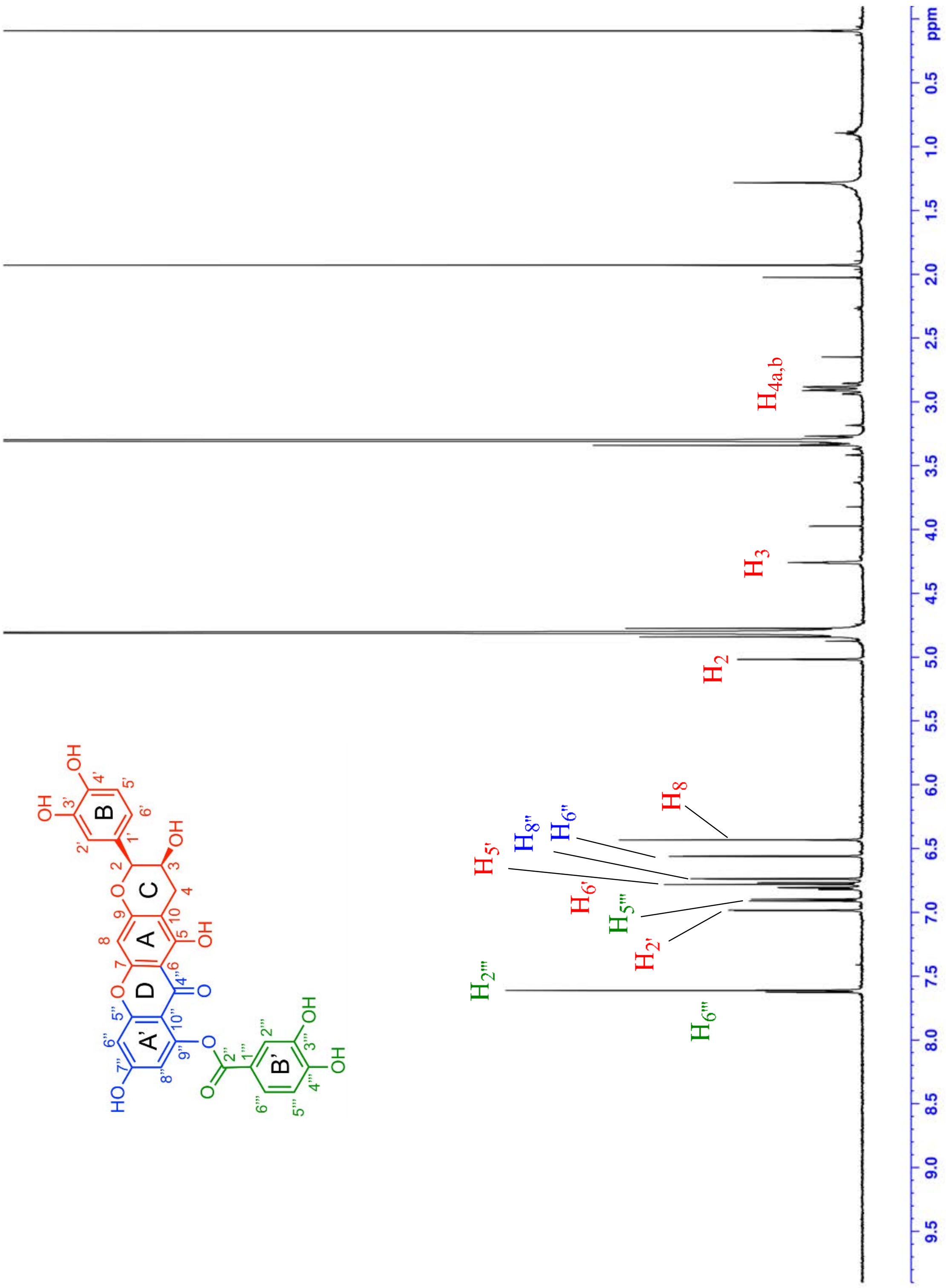

Figure S30: ^{1}H NMR of **5** (^{1}H: 600 MHz, CD_3OD, rt).

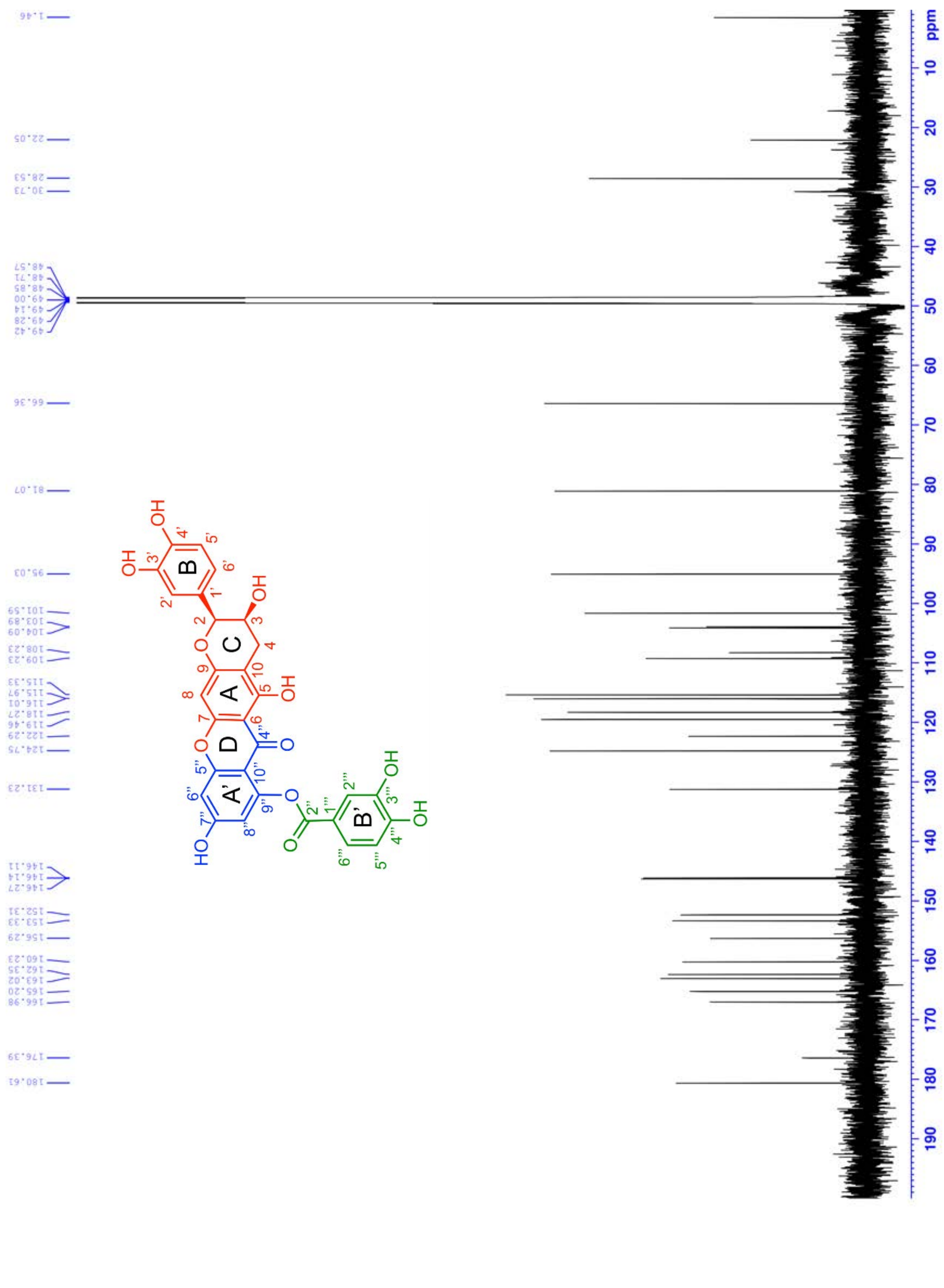

Figure S31: ^{13}C-NMR of **5** (^{13}C: 150 MHz, CD_3OD, rt).

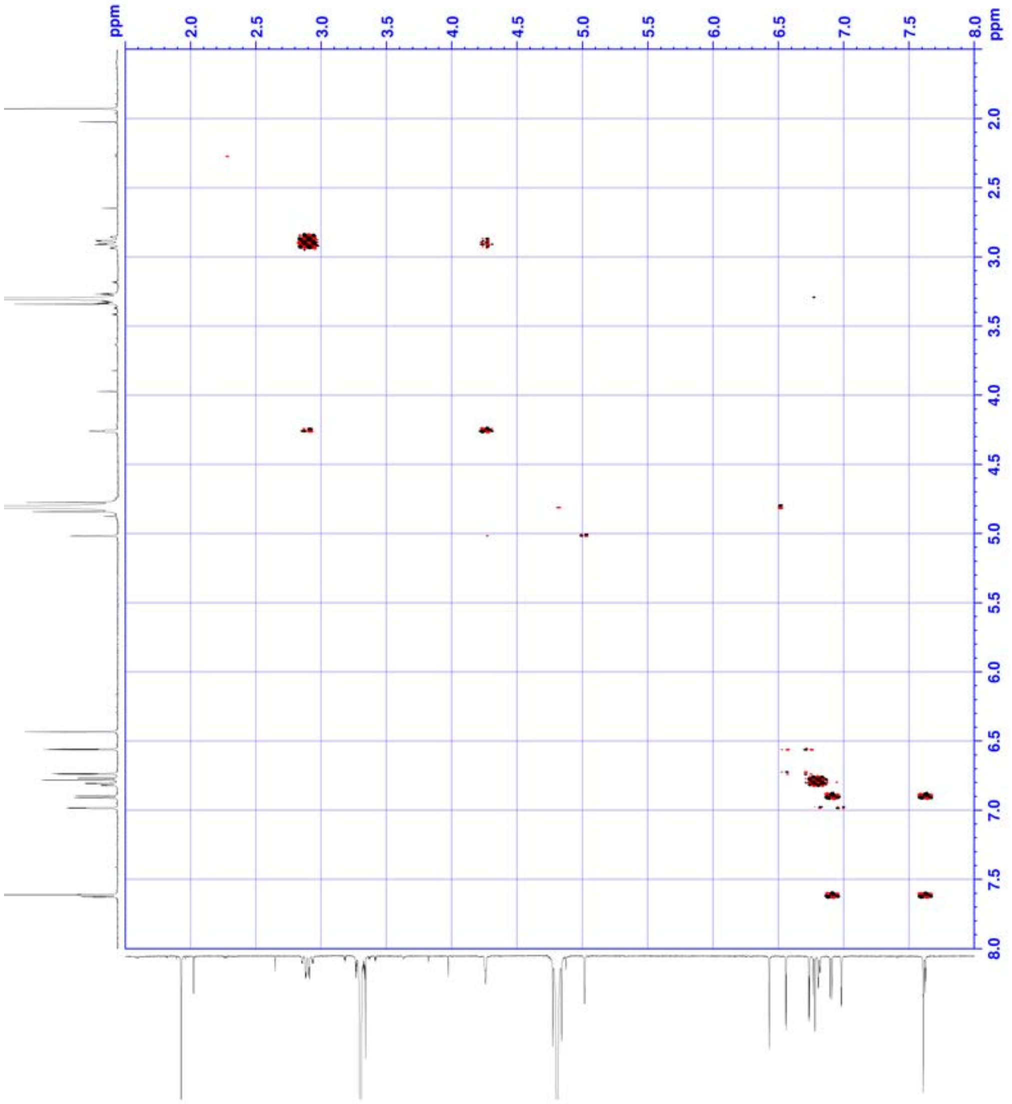

Figure S32: COSY of **5** (^{11}H: 600 MHz, CD_3OD, rt).

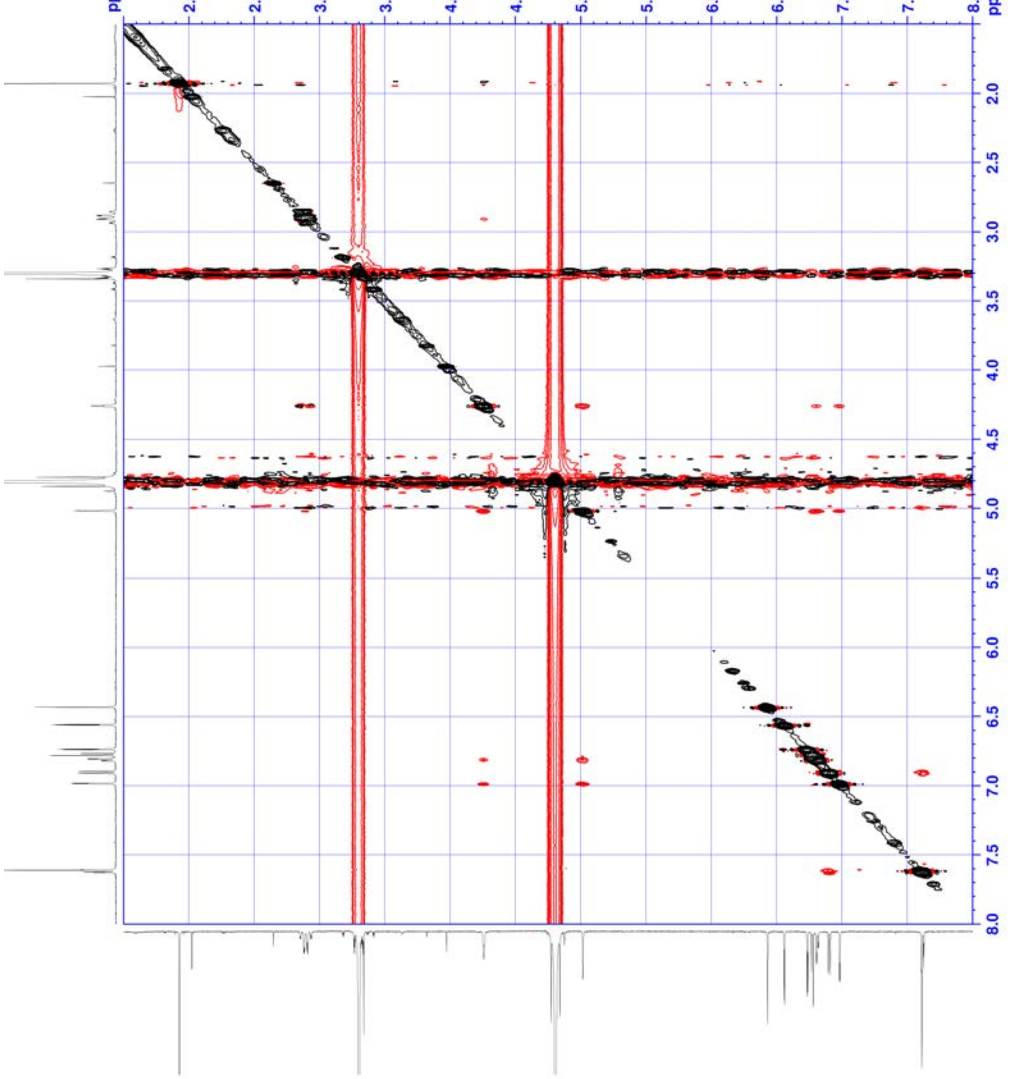

Figure S33: NOESY of **5** (^{1}H: 600 MHz, CD_3OD, rt).

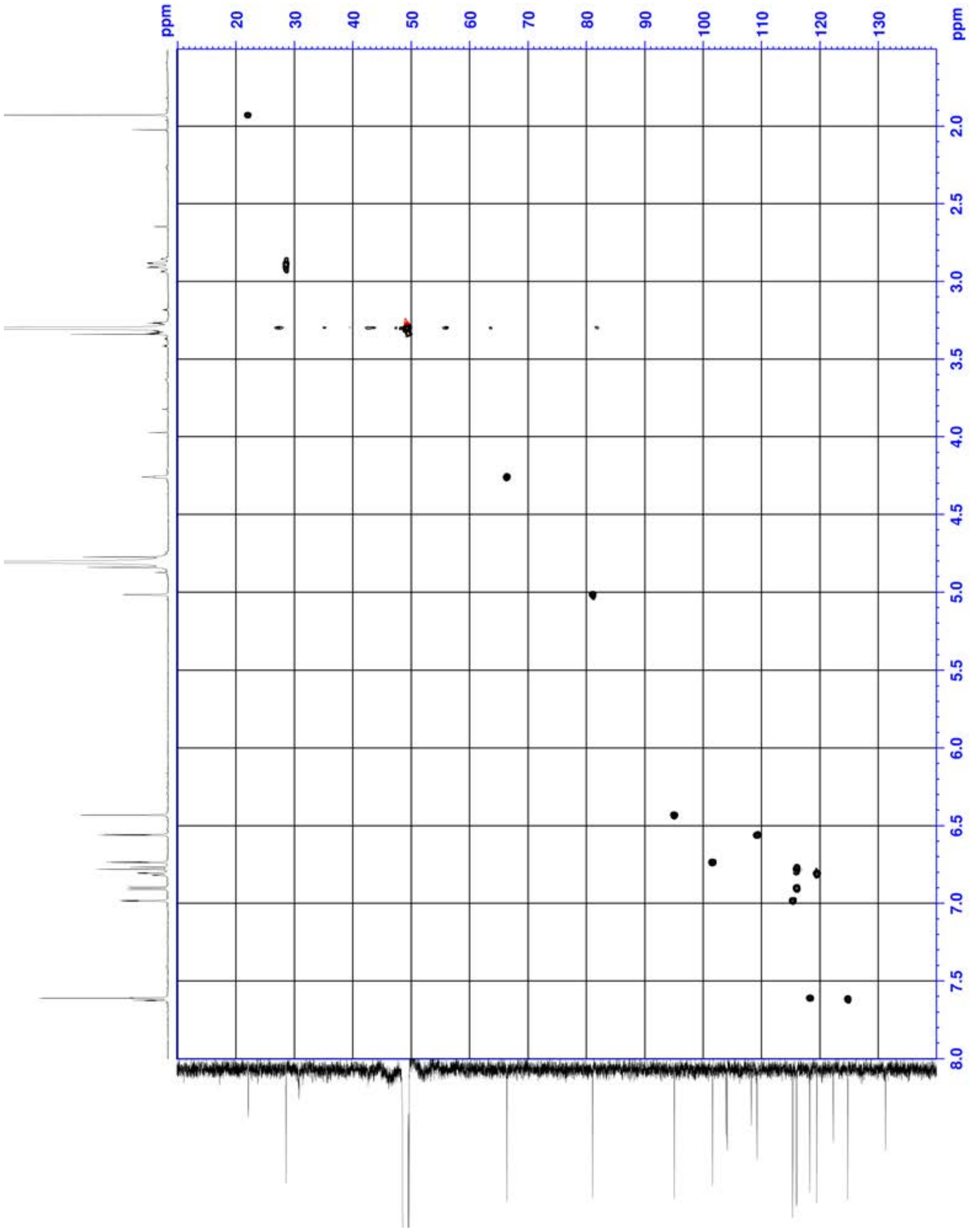

Figure S34: HSQC of **5** (^{1}H: 600 MHz, ^{13}C: 150 MHz, CD_3OD, rt).

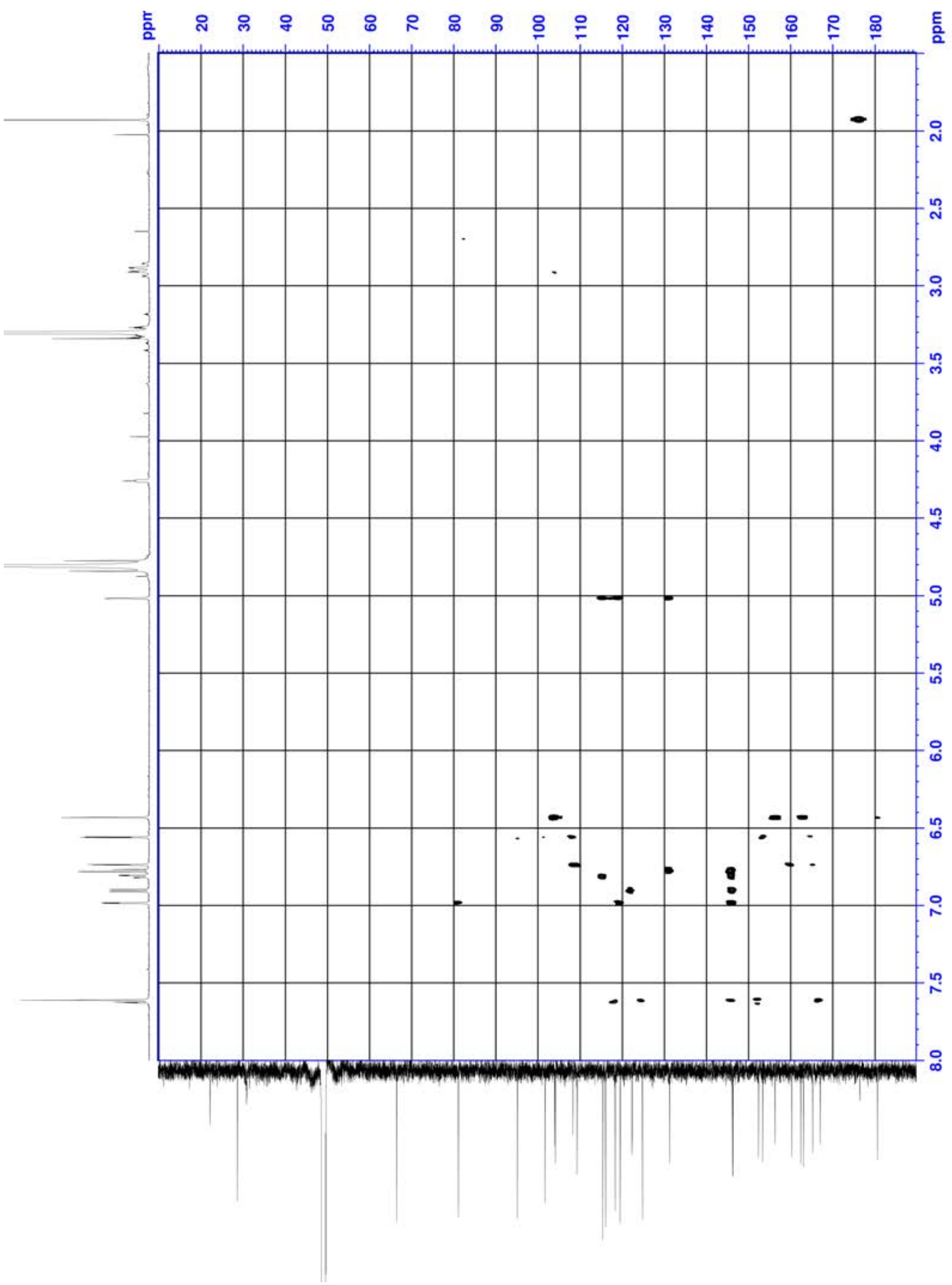

Figure S35-1: HMBC of **5** (^{1}H: 600 MHz, ^{13}C: 150 MHz, CD_3OD, rt).

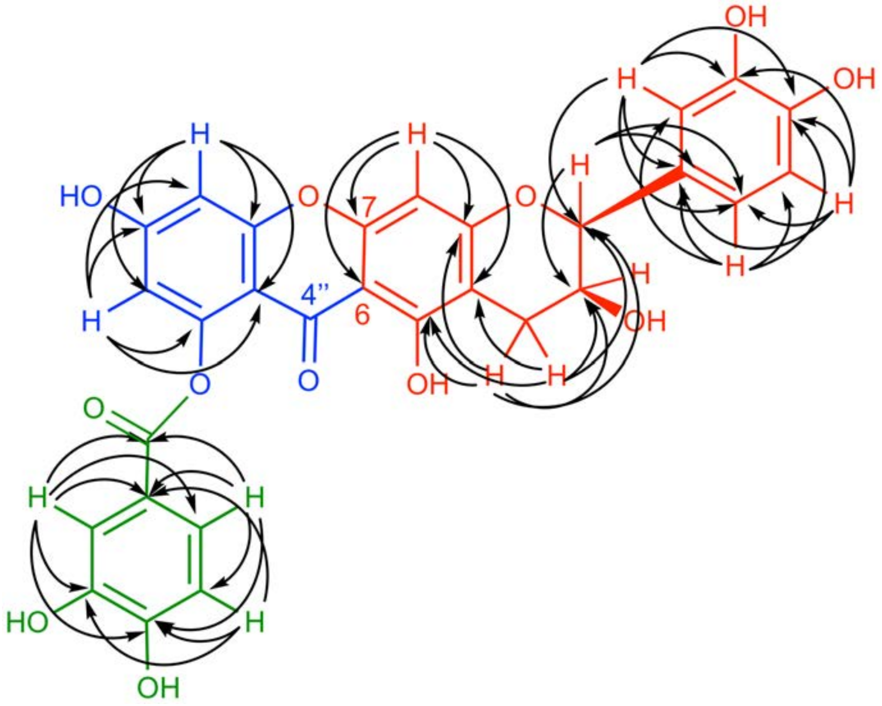

Figure S35-2: Observed HMBC correlations of **5**.

Hexamethyl-photodegraded catechinopyranocyanidin A (6)

(2*R*,3*S*)-2-(3,4-dimethoxyphenyl)-3-hydroxy-5,9-dimethoxy-6-oxo-2,3,4,6-tetrahydropyrano[3,2-*b*]xanthen-7-yl 3,4-dimethoxybenzoate

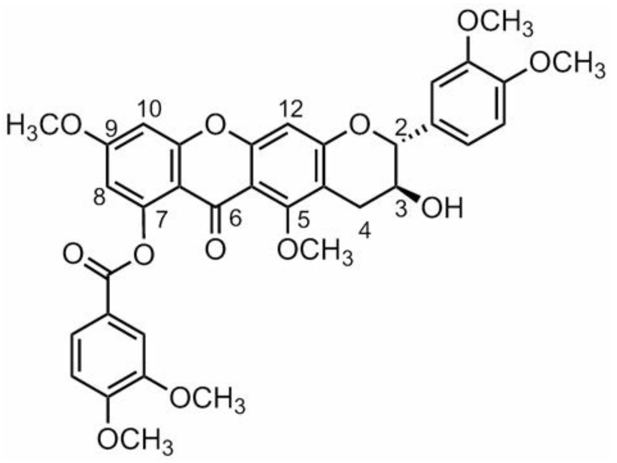

(The numbering of carbon atoms in the structure is different from that in the main text, but only for the IUPAC nomenclature)

HR-MS (ESI) Calcd for $C_{35}H_{33}O_{12}$ $[M+H]^+$: 645.1967, Found: 645.1968

Table S1. ^{1}H-NMR assignment of hexamethyl derivative of photo-degraded catechinopyranocyanidin A (**6**).

	**6** ($CDCl_3$)		
	δ (ppm)	multiplicity, J (Hz)	
2	4.79	d	8.5
3	4.12	ddd	9.0, 8.5, 5.5
4a (ax)	2.78	dd	16.0, 9.0
4b (eq)	3.32	dd	16.0, 5.5
8	6.72	s	
2'	6.99	d	2.0
5'	6.95	d	8.0
6'	6.99	dd	8.0, 2.0
6"	6.69	d	2.5
8"	6.79	d	2.5
2"'	7.79	d	2.0
5"'	7.00	d	8.5
6"'	7.96	dd	8.5, 2.0
		NOE obs. between	
5-OCH_3	3.82	H-4a, 4b	
3'-OCH_3	3.93	H-2'	
4'-OCH_3	3.94	H-5'	
7"-OCH_3	3.94	H-6", 8"	
3"'-OCH_3	4.00	H-2"'	
4"'-OCH_3	4.02	H-5"'	

^{1}H: 500 MHz, rt.

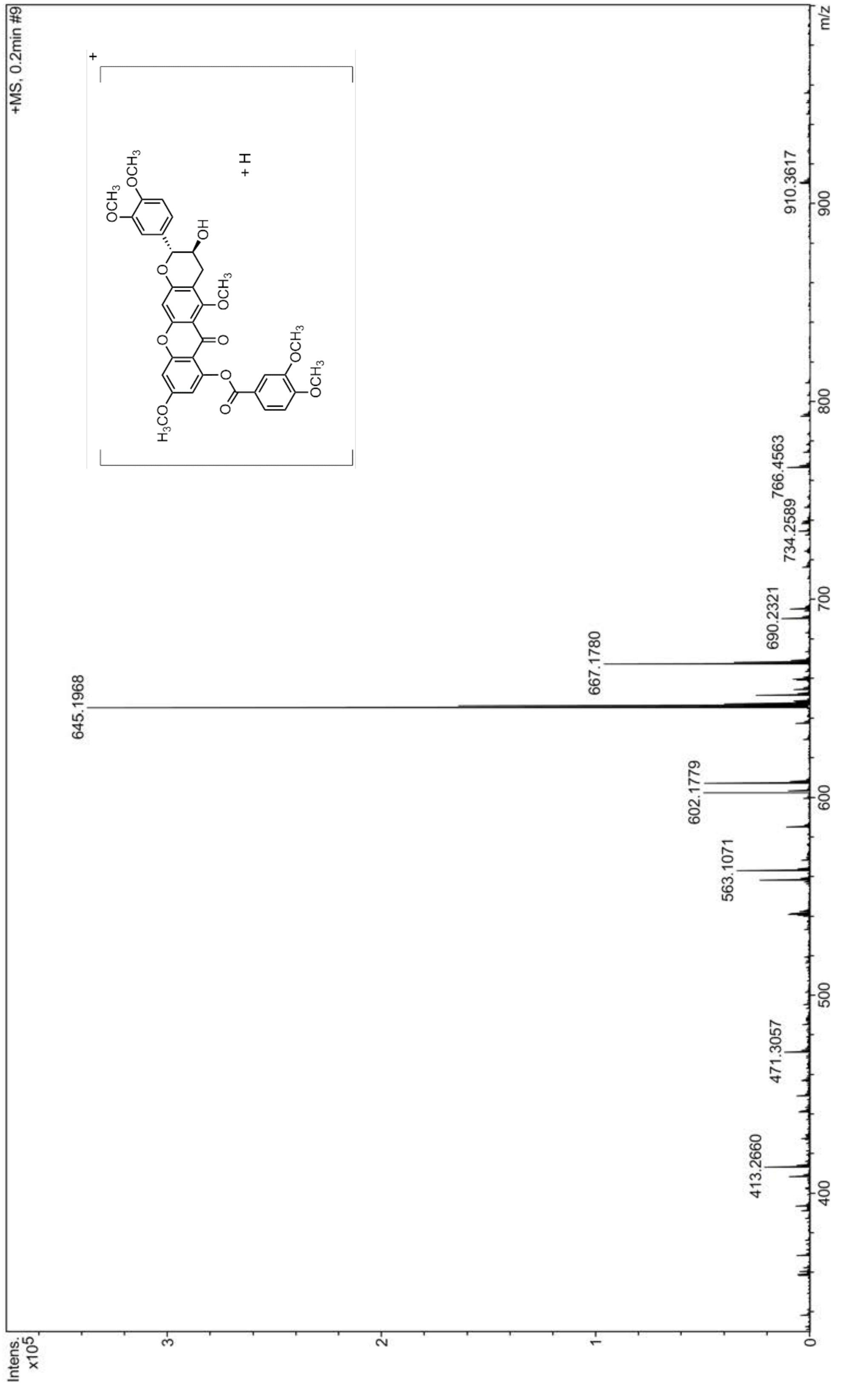

Figure S36: HR-ESI-TOF MS of **6**.

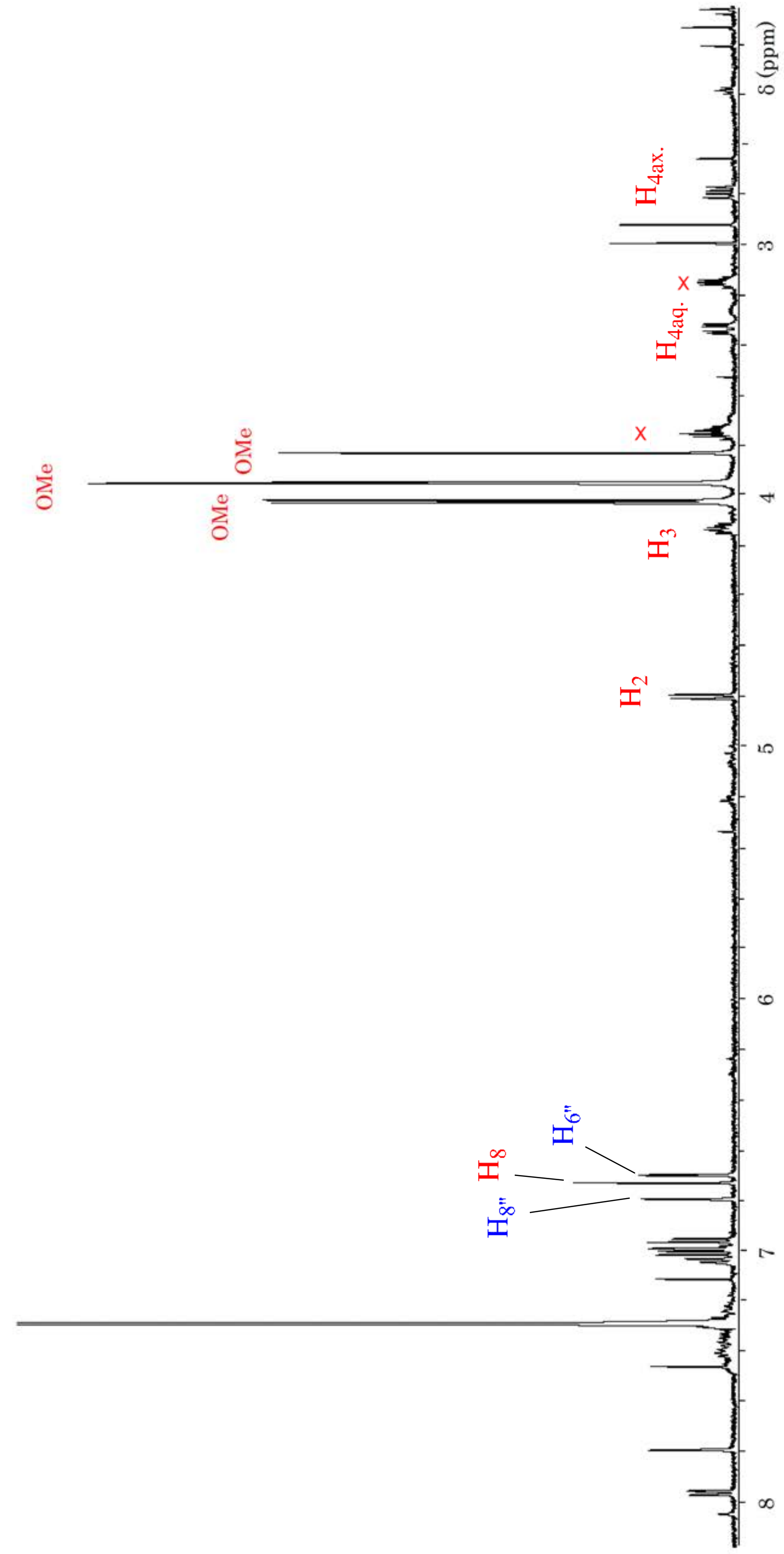

Figure S37: ^{1}H NMR spectrum of **6** (^{1}H: 600 MHz, $CDCl_3$, rt).

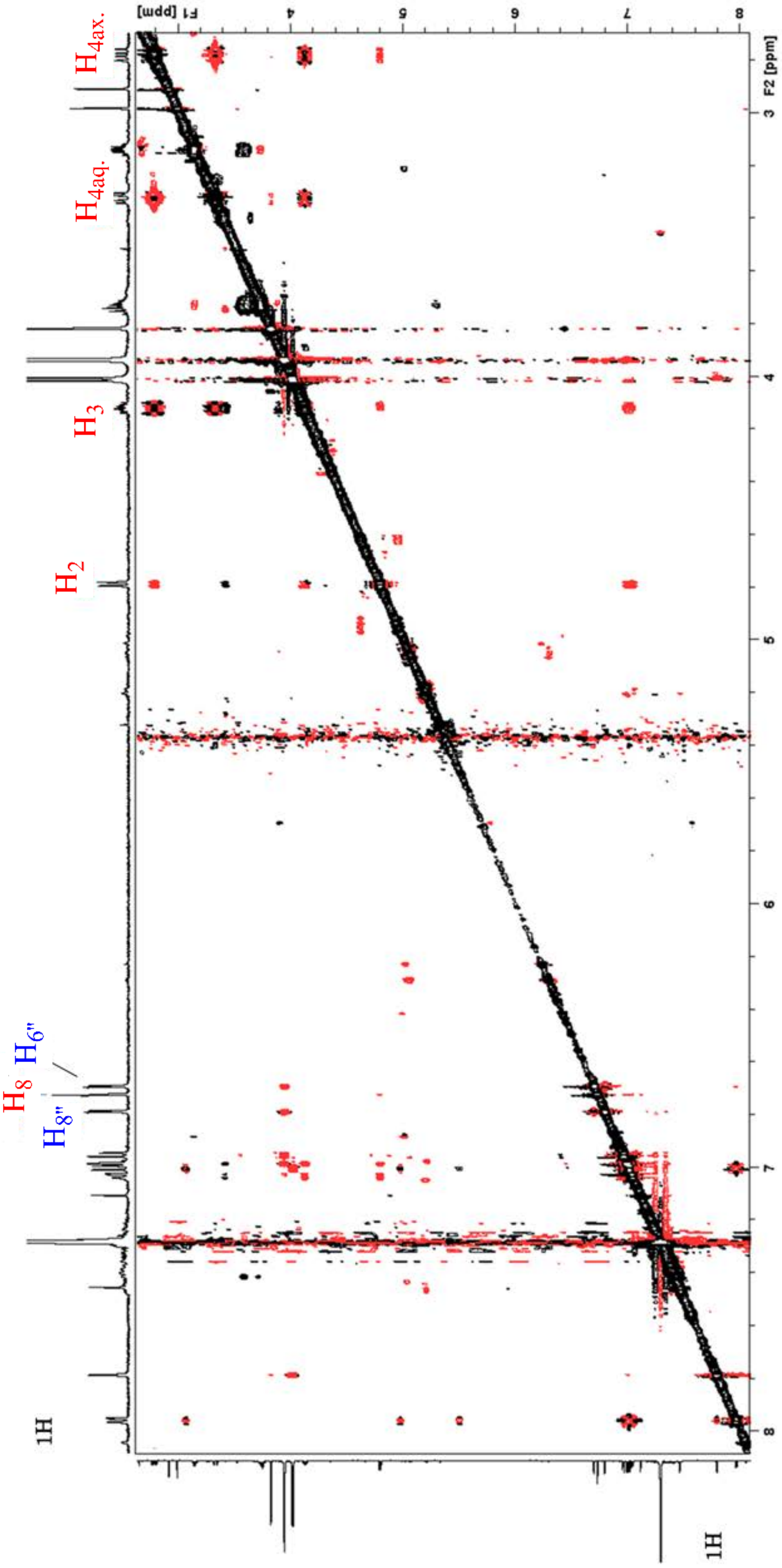

Figure S38-1: NOESY of **6** (^{1}H: 600 MHz, $CDCl_3$, rt).

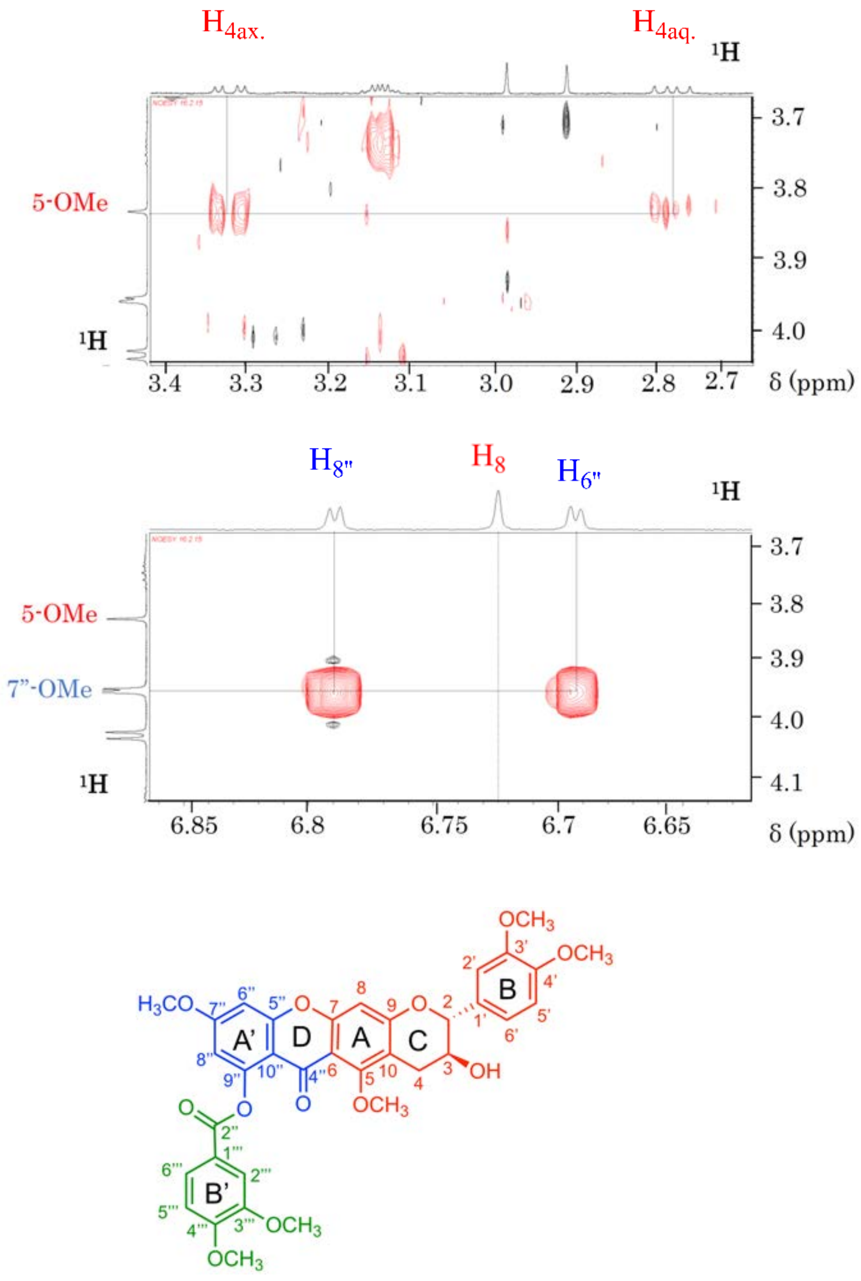

Figure S38-2: Expanded spectrum of NOESY of **6** and the observed NOEs (^{1}H: 600 MHz, $CDCl_3$, rt).

Calculation of chemical shift of ¹³C NMR of 2 and 3

Table S2. Comparison of experimental and calculated ^{13}C-NMR chemical shift of 5-keto, 7″-keto and 4‴-keto form of **2**.

Position	Experimental	Calculated		
		5-keto	7"-keto	4'''-keto
2	83.6	82.6	82.4	82.5
3	68.4	69.3	68.8	68.7
4	29.8	28.5	28.6	28.7
5	168.3	166.5	156.2	157.3
6	104.9	104.2	99.0	98.9
4	155.8	154.7	156.1	153.7
8	93.2	94.6	98.1	97.7
9	164.0	161.9	160.5	160.1
10	107.8	107.7	106.7	108.1
1'	131.3	130.3	129.8	129.5
2'	115.1	112.0	111.9	111.9
3'	146.5	143.6	143.8	143.6
4'	146.4	143.6	143.6	143.3
5'	116.2	114.0	114.2	113.9
6'	120.0	119.3	118.8	118.7
2"	150.7	155.5	156.3	154.3
3"	143.0	149.0	126.3	127.4
4"	144.5	149.0	138.4	127.3
5"	152.1	153.2	150.7	151.4
6"	99.7	96.8	108.7	96.9
7"	164.0	159.1	179.4	159.1
8"	97.4	94.4	106.5	96.4
9"	152.8	150.7	152.5	152.1
10"	106.9	108.9	109.1	102.5
1'''	124.8	124.3	121.1	108.5
2'''	117.5	118.8	114.6	102.9
3'''	146.2	140.9	141.5	150.4
4'''	154.8	147.7	148.2	178.9
5'''	116.3	114.8	115.3	121.4
6'''	124.5	122.2	125.8	131.9
	RMS	2.98	6.06	8.16

Table S3. Comparison of experimental and calculated ^{13}C-NMR chemical shift of 5-keto, 7″-keto and 4‴-keto form of **3**.

Position	Experimental	Calculated		
		5-keto	7''-keto	4'''-keto
2	80.8	80.0	79.6	80.0
3	67.1	65.1	67.2	66.9
4	30.1	25.7	27.2	27.3
5	168.6	175.0	156.0	157.1
6	105.0	106.2	98.7	98.1
4	155.5	155.0	156.1	154.2
8	93.5	92.1	97.7	97.7
9	164.5	163.5	160.5	160.5
10	107.0	106.7	105.4	106.8
1'	131.6	127.5	128.2	131.8
2'	115.3	111.5	110.8	113.5
3'	146.0	142.2	141.9	145.9
4'	146.0	144.0	145.2	141.4
5'	115.9	114.9	116.4	112.8
6'	119.5	119.8	119.5	116.4
2''	154.5	150.6	156.1	153.7
3''	142.9	140.2	126.4	127.7
4''	144.5	148.3	138.3	130.2
5''	152.0	154.0	150.7	151.6
6''	99.63	96.8	108.7	96.8
7''	163.9	160.1	179.4	159.6
8''	97.4	94.7	106.4	96.7
9''	152.7	152.2	152.5	152.9
10''	106.8	106.2	108.9	102.4
1'''	124.8	123.1	120.8	110.3
2'''	117.5	118.4	114.3	103.1
3'''	146.1	141.0	141.1	150.2
4'''	150.7	147.6	148.5	179.8
5'''	116.3	114.9	115.5	121.6
6'''	124.5	122.6	126.0	135.6
	RMS	2.80	5.99	8.53

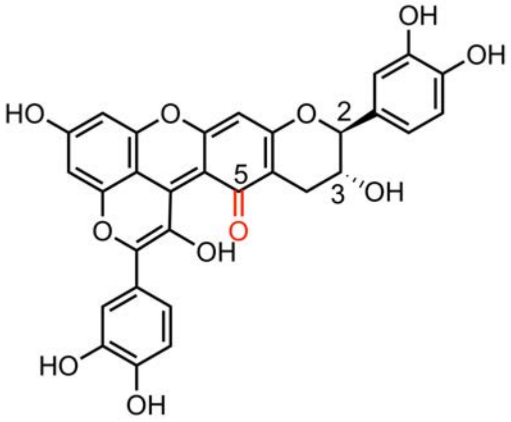

5-keto tautomer

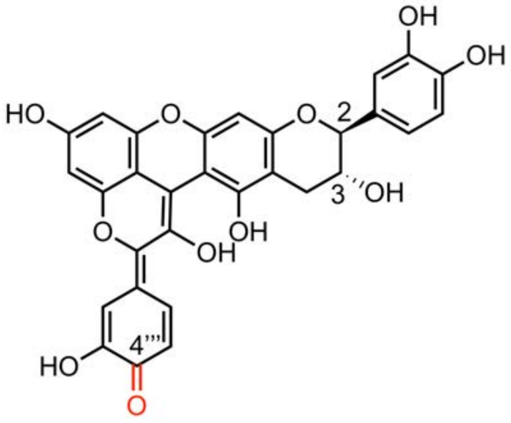

4'''-keto tautomer

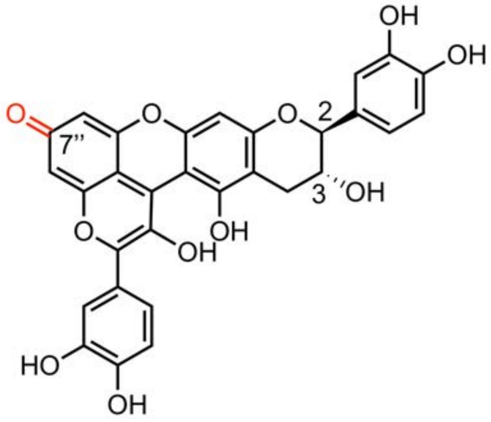

7''-keto tautomer

Figure S39: The Calculated tautomers of **2**. The tautomers of **3** were those the configuration of C2 and C3 were *cis*.

Absolute configuration of 2 and 3

To examine the absolute configurations at C2 and C3, the ECD curves were computed by using the Gaussian 16 program.[30] The number of conformers were 13 and 11 for **2** and **3**, respectively, which are energetically low-lying structures obtained in the chemical shift calculations. To examine the effects of the 3,4-dihydroxyphenyl (catechol) group at C2 and hydroxyl group at C3, the ECD curves for non-chiral model molecules which have only H atoms at C2 and C3 were also examined (Figure S40). To obtain the structures of such a model, the catechol and hydroxyl groups at C2 and C3 were replaced by H atoms, and only the positions of newly placed H atoms were optimized with the Cartesian coordinates of other atoms being fixed. These partial geometry optimizations were performed by using the B3LYP functional[S1] and def2-TZVP basis set.[S2]

At the obtained structures, the excitation energies (ΔE_{0n}) and the rotatory strengths (R_{0n}) from the ground to the n-th singlet excited states were computed by using the TD-DFT calculations, where the B3LYP functional[S1] and the def2-TZVP basis set[S2] were used. The ECD curves $\Delta\varepsilon$ were computed by multiplying the Gaussian function as [S3]

$$\Delta\varepsilon(E) = \sum_{n=1}^{15} \left\{ \frac{\Delta E_{0n}}{2.296\times10^{-39}\sqrt{\pi}\Delta} R_{0n} \exp\left[-\left(\frac{E - \Delta E_{0n}}{\Delta} \right)^2 \right] \right\}$$

where Δ is a half bandwidth at $1/e$ and was set to be 0.1 eV.

Table S4 summarizes the relative potential energy, dihedral angle of 10″−4″−6−5 (τ_6) and excitation energy and rotatory strength of the first excited state for the 13 conformers of **2**. Note that the first excited state has non-negligible magnitude of oscillator strength, $f \approx 0.57$. For comparison, the excitation energy and rotatory strength for the counterpart model molecules are also shown. As seen in Table S4, the excitation wavelength of the first excited state is little affected by difference of conformations and the catechol and hydroxyl groups at C2 and C3. This result is rationalized by considering that the excitation is attributed to the HOMO→LUMO transition, where both HOMO and LUMO are delocalized on only A-D-A′-C′-B′-ring.

Table S5 and Figure S41 show the properties of **3**, which correspond to those of **2** in Table S4 and Figure 4c, respectively. For energetically low-lying 11 conformers of **3** at the present calculation level, the second excited state has non-negligible magnitude of oscillator strength.

Thus, the excited state properties are shown for the second excited state. As seen in Table S5, the excitation wavelength is again little affected by difference of conformations and the catechol and hydroxyl groups at C2 and C3. Figure S41 shows the ECD curves $\Delta\varepsilon$, $\Delta\varepsilon_{model}$ and $\Delta\varepsilon$–$\Delta\varepsilon_{model}$ for **3**. As discussed in the main text, it is reasonable to surmise that the negative peaks for all 11 conformers at the visible region in $\Delta\varepsilon$–$\Delta\varepsilon_{model}$ is ascribed to the (*S*, *S*) configurations at C2 and C3 for **3**.

Table S4. Relative potential energy E,[a)] dihedral angle τ_6[b)] and excitation energy ΔE and rotatory strength R of the first excited state for the structures employed for **2**, and ΔE and R for the counterpart model molecule.

**2**					Model [d)]	
No.	E (kJ/mol)[a)]	τ_6 (deg)	ΔE (nm)	R (10^{-40} cgs) [c)]	ΔE (nm)	R (10^{-40} cgs) [c)]
1	1.80	−171	544	−9.48	542	−14.21
2	2.43	171	545	17.98	543	14.17
3	0.50	−171	545	−8.08	542	−14.19
4	2.11	171	542	18.36	540	14.17
5	1.56	171	541	25.69	539	15.18
6	1.54	−171	542	−8.25	540	−13.18
7	0.00	171	541	26.94	538	14.34
8	2.42	−171	545	−13.23	542	−13.75
9	1.92	171	543	24.75	541	15.19
10	2.26	171	544	16.77	541	14.77
11	2.02	171	542	17.39	540	12.81
12	0.44	171	544	26.84	541	14.74
13	0.50	−171	542	−3.95	539	−14.63

a) At the B3LYP/def2-TZVP level.

b) Dihedral angle of 10″−4″−6−5 in Figures 3a and S40.

c) The length form was employed.

d) Only the coordinates of H atoms at C2 and C3 were optimized for the model molecule. See text in Supporting Information.

Table S5. Relative potential energy E,[a)] dihedral angle τ_6[b)] and excitation energy ΔE, oscillator strength f, and rotatory strength R of the second excited state for the structures employed for **3**, and ΔE and R for the counterpart model molecule.

**3**						Model [d)]	
No.	E (kJ/mol)[a)]	τ_6 (deg)	ΔE (nm)	f	R (10^{-40} cgs) [c)]	ΔE (nm)	R (10^{-40} cgs) [c)]
1	2.03	171	483	0.60	−12.02	480	36.65
2	2.19	−171	483	0.59	−76.90	479	−31.48
3	0.61	−171	484	0.58	−88.09	480	−33.27
4	2.09	170	481	0.61	−10.14	478	36.43
5	2.17	−171	485	0.59	−76.71	482	−29.24
6	0.31	171	483	0.60	−19.17	479	37.03
7	2.01	171	485	0.60	−12.71	482	35.84
8	2.25	−171	485	0.58	−74.17	482	−29.19
9	2.43	170	483	0.60	−9.19	480	37.19
10	0.00	171	485	0.60	−19.11	481	36.01
11	0.09	−170	485	0.58	−83.43	482	−30.62

a) At the B3LYP/def2-TZVP level.

b) Dihedral angle of 10″−4″−6−5 in Figures 3b and S40.

c) The length form was employed.

d) Only the coordinates of H atoms at C2 and C3 were optimized for the model molecule. See text in Supporting Information.

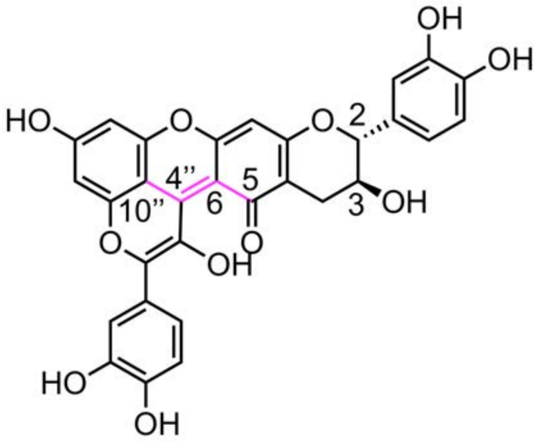

2

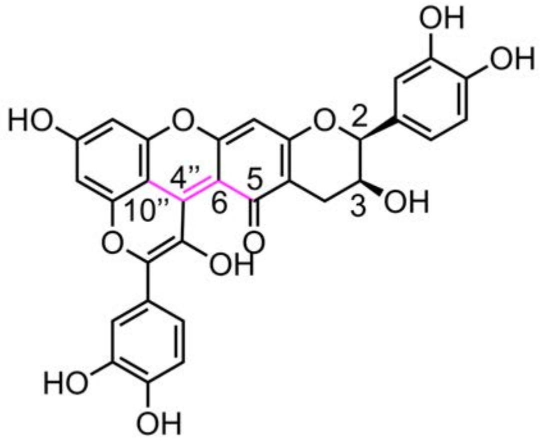

3

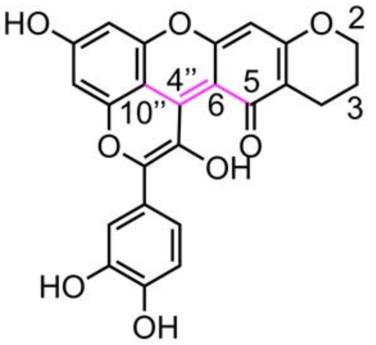

model compound

Figure S40. Structure of **2**, **3** and non-chiral model compound. Dihedral angle indicated as τ_6 is indicated with pink bonds: C10″-C4″-C6-C5.

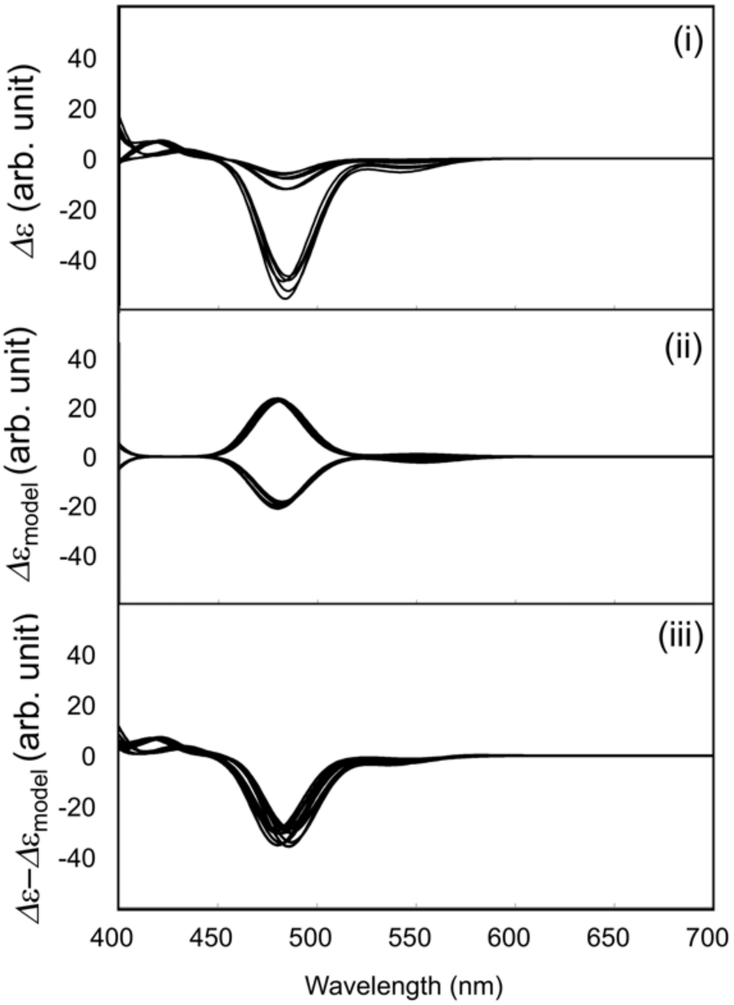

Figure S41: The computed ECD curves at the visible region for 11 conformers of **3** ($\Delta\varepsilon$, (i)), the counterpart model ($\Delta\varepsilon_{model}$, (ii)), and their difference ($\Delta\varepsilon$–$\Delta\varepsilon_{model}$, (iii)).

Purple color development of 2 and 3

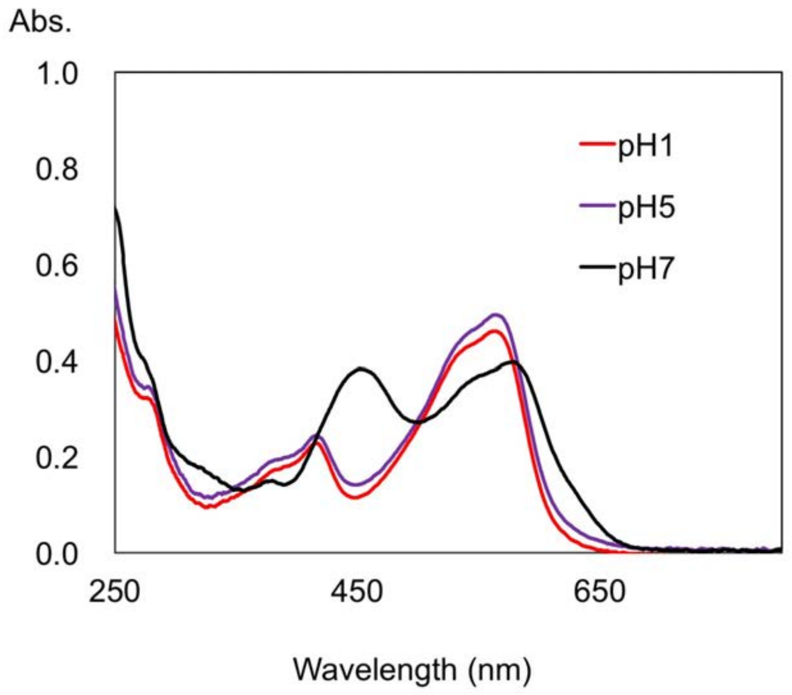

Figure S42: UV-Vis absorption spectra of catechinopyranocyanidn B (**3**) in various pH buffer solutions. **3**: 50 μM, in 100 mM KCl-HCl (pH 1.0), AcOH-AcONa (pH 5), Na_2HPO_4-KH_2PO_4 (pH 7.0)-MeOH 1:1, path length: 10 mm, rt.

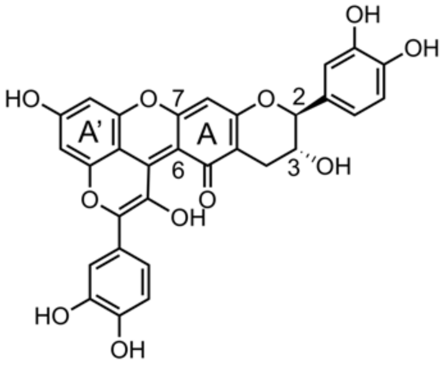

catechinopyranocyanidin A (**2**)

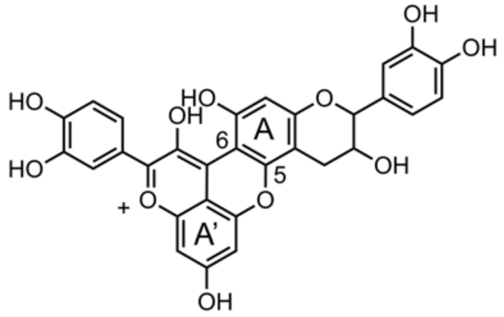

vignacyanidin from Takahama et al.

Figure S43: Comparison of structures of our elucidated catechinopyraocyanidin A (**2**) and vignacyanidin.[15]

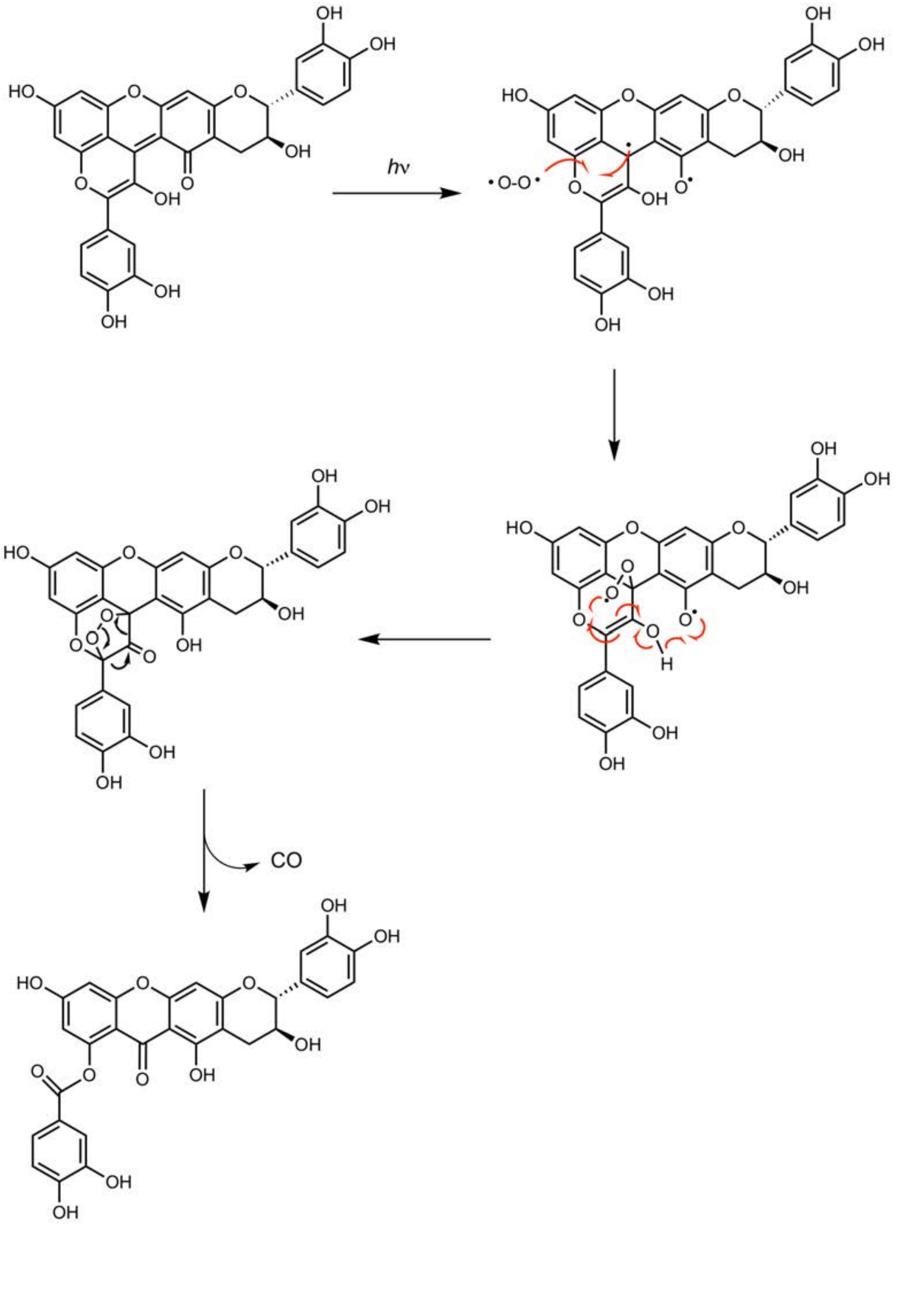

Figure S44: Proposed mechanism of photo-degradation of catechinopyranocyanidn A (**2**).
